# Recent meta-analyses neglect previous systematic reviews and meta-analyses about the same topic: a systematic examination

**DOI:** 10.1186/s12916-015-0317-4

**Published:** 2015-04-14

**Authors:** Bartosz Helfer, Aaron Prosser, Myrto T Samara, John R Geddes, Andrea Cipriani, John M Davis, Dimitris Mavridis, Georgia Salanti, Stefan Leucht

**Affiliations:** Department of Psychiatry and Psychotherapy, Technical University Munich, Klinikum rechts der Isar, Ismaningerstr 22, 81675 Munich, Germany; Complex Mental Illness Program, Centre for Addiction and Mental Health, Toronto, Canada; Department of Psychiatry, University of Oxford, Warneford Hospital, Oxford, UK; Department of Psychiatry, University of Illinois at Chicago, Chicago, IL USA; Department of Hygiene and Epidemiology, University of Ioannina School of Medicine, Ioannina, Greece; Department of Primary Education, University of Ioannina, Ioannina, Greece

**Keywords:** Meta-analysis, Methodology, PRISMA statement, Research waste, Systematic review

## Abstract

**Background:**

As the number of systematic reviews is growing rapidly, we systematically investigate whether meta-analyses published in leading medical journals present an outline of available evidence by referring to previous meta-analyses and systematic reviews.

**Methods:**

We searched PubMed for recent meta-analyses of pharmacological treatments published in high impact factor journals. Previous systematic reviews and meta-analyses were identified with electronic searches of keywords and by searching reference sections. We analyzed the number of meta-analyses and systematic reviews that were cited, described and discussed in each recent meta-analysis. Moreover, we investigated publication characteristics that potentially influence the referencing practices.

**Results:**

We identified 52 recent meta-analyses and 242 previous meta-analyses on the same topics. Of these, 66% of identified previous meta-analyses were cited, 36% described, and only 20% discussed by recent meta-analyses. The probability of citing a previous meta-analysis was positively associated with its publication in a journal with a higher impact factor (odds ratio, 1.49; 95% confidence interval, 1.06 to 2.10) and more recent publication year (odds ratio, 1.19; 95% confidence interval 1.03 to 1.37). Additionally, the probability of a previous study being described by the recent meta-analysis was inversely associated with the concordance of results (odds ratio, 0.38; 95% confidence interval, 0.17 to 0.88), and the probability of being discussed was increased for previous studies that employed meta-analytic methods (odds ratio, 32.36; 95% confidence interval, 2.00 to 522.85).

**Conclusions:**

Meta-analyses on pharmacological treatments do not consistently refer to and discuss findings of previous meta-analyses on the same topic. Such neglect can lead to research waste and be confusing for readers. Journals should make the discussion of related meta-analyses mandatory.

**Electronic supplementary material:**

The online version of this article (doi:10.1186/s12916-015-0317-4) contains supplementary material, which is available to authorized users.

## Background

Systematic reviews and meta-analyses represent a high level of evidence and are invaluable to health professionals in synthesizing the results of medical research [1]. The number of systematic reviews is growing rapidly - in 2010 approximately 11 such studies were published per day, which corresponds to the number of randomized controlled trials (RCTs) published three decades ago [2]. Because of this exponential growth in publication rates, many meta-analysis authors may not discuss the results of previous meta-analyses and systematic reviews on the same topic - in a manner analogous to authors of RCTs not referring to a substantial portion of other relevant RCTs [3] or systematic reviews [4,5]. This can be very confusing for readers and cause waste in research resources [6], including waste in study planning [7], design, and conduct [8] as well as leading to unnecessary duplications [9] and incomplete reporting [10,11].

To grasp the importance of such neglect, imagine clinicians seeking a treatment solution for a patient’s specific medical problem. They find two similar meta-analyses with discordant results. If the newer article does not refer to the older, the readers are given no explanation about the possible reasons for this discrepancy. Which article should they trust more? Their level of uncertainty is higher than before reading the authoritative articles and an evidence-based decision regarding their patients’ treatment even more difficult. Not referring to important related research is also against the principles of evidence-based medicine, because meta-analysts agree that all available evidence should be systematically searched and reviewed in an unbiased manner [12].

Moreover, as for all types of research, the question a meta-analysis is trying to answer should be relevant [7-9]. If the question has been already answered in a previous meta-analysis, the authors should clearly justify why they decided to perform a similar analysis again. Is it a replication, an update, or maybe just an unnecessary duplication?

To provide patients, clinicians, and policymakers with the most useful information about a clinical question, meta-analyses should not neglect previous systematic reviews about the same topic. This will not only help to provide a more complete understanding of the clinical problem, but also to avoid research waste and biased results.

We report a systematic investigation of whether recent meta-analyses published in the leading medical journals cite, describe, and discuss previous meta-analyses and systematic reviews on the same topic. We also analyze factors that are likely to be associated with this phenomenon.

## Methods

First, we identified a sample of recent meta-analyses, then for each included recent article we performed a separate systematic search to find similar previous meta-analyses and systematic reviews. Our goal was to estimate what proportion of the previous meta-analyses and systematic reviews was cited, described, and discussed by the recent meta-analyses. We also investigated potential predictors of citing, describing, and discussing. We initially published a protocol at our institutional website [13].

### Selection of the recent meta-analyses

We searched PubMed, combining the names of the six general medical journals with the highest impact factors according to Journal Citation Records, 2013 edition (New England Journal of Medicine, The Lancet, JAMA: The Journal of the American Medical Association, Annals of Internal Medicine, PLOS Medicine, British Medical Journal) and ‘meta-analysis’ as publication type (see Additional file 1). To produce a more homogenous sample we only included meta-analyses on pharmacological treatments. The original search was completed in March 2013. We aimed to include at least 50 published meta-analyses. We expanded the search to January 2012 to meet this criterion.

We then systematically assessed citation habits of these recent meta-analyses towards previous meta-analyses and systematic reviews on the same topic.

### Selection of the previous meta-analyses and systematic reviews

For each recent meta-analysis we searched PubMed for previous meta-analyses and systematic reviews on the same topic (unlike recent articles, previous studies also included systematic reviews without meta-analysis), combining the keywords provided by the recent articles with ‘meta-analysis’ or ‘systematic review’ as publication type (see Additional file 2). The keywords were based on the characteristics of the participating population and the intervention(s) used. The reference lists of all included studies were also screened. We compared PICO questions between the recent and the previous articles in order to make sure that they focus on a similar group of participants (P) and use similar interventions (I), comparators (C), and outcomes (O) [14]. Previous articles which had any of the PICO questions completely different from the corresponding questions in the recent article were excluded. Additionally, for included previous articles we calculated a ‘similarity score,’ such as for each PICO question one or zero points were given, depending whether all of the four PICO questions were identical to the corresponding questions from the recent article (one point per question was given if that was the case) or whether the questions were only overlapping. That was the case when the criteria for each PICO question were only partially similar, for example when multiple outcomes were used and only some of them were employed by the previous study (in such a case zero points were given, but the study was not excluded). For more details and examples of this similarity score see Additional file 3. We also excluded articles published more than 10 years or less than 1 year from the time of publication of the recent meta-analysis, unless they were cited in the recent meta-analysis. This criterion ensured that we did not analyse outdated material and it also does justice to the fact that the publication process can take a long time.

### Statistical analysis of predictors

Our primary question was to estimate what proportion of the previous meta-analyses and systematic reviews was cited (that is, whether a reference to the previous article was provided by the recent study), described (that is, whether any information about the results of the previous article was given), and discussed (that is, whether the results from the previous article were related to the results or conclusions from the recent study) by the recent meta-analysis. Table 1 provides specific examples for each definition.

**Table 1:** **Cited versus described versus discussed: definitions and examples**

	**Definition**	**Example**
**Cited**	A reference to the recent article was provided by the previous study	A recent meta-analysis on probiotics [15] cited a previous meta-analysis [16] by putting the reference (number 91) at the end of this sentence: ‘The objective of this study was to evaluate broadly the available evidence on probiotic interventions for the prevention and treatment of AAD, building on previous nonsystematic overviews and systematic reviews on selected applications [1,2,8,11,89-91]*.*’
**Described**	Additional information about the results of the previous article was given by the recent study	A recent meta-analysis on dual blockade of the renin-angiotensin system [17] described the results from a previous meta-analysis [18] by saying, ‘One meta-analysis reported ‘encouraging’ evidence that dual therapy reduced proteinuria by an incremental 20-25% compared with monotherapy*.*’
**Discussed**	Results from the previous article were related to the results or conclusions from the recent study	A recent meta-analysis on oral anticoagulants [19] discussed the results from a previous systematic review [20] by saying, ‘Loke and Kwok published an adjusted indirect comparison of rivaroxaban and dabigatran based on studies on acute venous thromboembolism prophylaxis in orthopaedics, with low molecular weight heparin as the common comparator. Rivaroxaban was found to be superior to dabigatran, but there was a trend towards increased bleeding. Differences in therapeutic regimen and selection of patients could explain these discrepancies with our results.’

We also investigated potential predictors of citing, describing, and discussing previous meta-analyses and systematic reviews by recent meta-analyses using mixed-effects logistic regression analysis in R.

Recent article-specific predictors included: journal title, medical discipline, journal impact factor (based on Journal Citation Records, 2013 edition), and quality of the systematic review as measured with the AMSTAR score (a measurement tool for the assessment of the methodological quality of systematic reviews) [21].

Previous article-specific predictors included: level of similarity of the review question (based on comparison of PICO questions between recent and previous article), journal impact factor, publication year, article type (systematic review using meta-analytic methods versus not), and concordance of results between recent and previous articles (similar results versus different results). Results were judged as ‘similar’ when the direction of the effect was the same, irrespective of the effect size. In general, concordance of results was based on the major findings of the study (primary outcome, if possible) and, if necessary, particular results and conclusions were compared, including strength of evidence. In case of previous systematic reviews without meta-analysis, concordance was based on the main message of the paper, that is, the authors’ summary and/or conclusions (illustrating examples are presented in Additional file 4). To avoid confusion we emphasize that the term ‘similarity’ refers to a comparison of the recent and previous articles in terms of the review questions, whereas the term ‘concordance’ refers to a comparison of recent and previous article results.

In a sensitivity analysis we excluded previous articles published before 2010 to check whether the general pattern of results changed in the newer papers.

BH piloted the analysis on a sample of 10 studies, selecting and extracting all the data. AP independently extracted a random sample of 25%. An inter-rater reliability analysis using the Kappa coefficient was performed to determine the consistency among raters [22]. Conflicts were resolved by discussion between BH and AP; if necessary, SL was involved. Results of the regression analyses are presented as odds ratios (OR) and associated 95% confidence intervals (CI).

## Results

We identified 52 recent meta-analyses and 242 previous meta-analyses and systematic reviews (including 24 previous systematic reviews without meta-analysis), covering a wide range of drugs and medical specialties. Table 2 shows summary characteristics of included studies, whereas Additional files 5 and 6 provide detailed information on the individual meta-analyses and systematic reviews.

**Table 2:** **Summary characteristic of included studies**

**Journal**	**Recent**		**Previous**	
Annals of Internal Medicine	9	17%	54	22%
British Medical Journal	24	46%	117	48%
JAMA	7	13%	32	13%
New England Journal of Medicine	1	2%	9	4%
PLOS Medicine	5	10%	12	5%
The Lancet	6	12%	18	7%
Total:	52		242	
Mean AMSTAR score for recent meta-analyses	8.5 (SD 1.5)			
Mean impact factor for previous studies	7.7 (SD 8.8)			
Previous SRs with meta-analysis	218			
Previous SRs without meta-analysis	24			
Number of previous studies for a recent meta-analysis	0 to 28 (mean 4.65; SD 4.65)			
Mean length of time from publication year previous to recent study	3.6 years (SD 2.5)			
Mean similarity score between previous and recent studies	1.8 points (SD 1.1, range 0 to 4)			

‘Recent’ refers to the initially identified meta-analyses and ‘previous’ refers to the previous meta-analyses or systematic reviews (SRs) on the same topic. More detailed information on all the individual studies is presented in Additional files 5 and 6. SD, standard deviation.

Out of 52 recent meta-analyses there were only four without previously published meta-analyses or systematic reviews. These four articles were excluded from the regression analysis. For the remaining 48 articles there were, on average, five (range 1 to 28, SD 4.6) previous meta-analyses or systematic reviews per paper.

**Figure 1:**
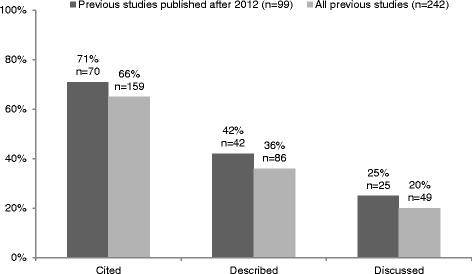
Results of the primary analysis. Percentage of previous meta-analyses and systematic reviews that were cited, described, and discussed by the recent meta-analyses.

Out of 242 previous meta-analyses and systematic reviews, approximately two-thirds were cited (159 out of 242, 66%), one-third described (86 out of 242, 36%), and only one-fifth discussed in the recent meta-analyses (49 out of 242, 20%). This pattern of results did not change when the previous articles published before 2010 (that is, older than two years) were excluded (see Figure 1).

Citing a previous meta-analysis or systematic review by a recent meta-analysis was positively associated with publication of the previous article in a journal with a higher impact factor (OR, 1.49; 95% CI, 1.06 to 2.10) and more recent publication year (OR, 1.19; 95% CI, 1.03 to 1.37). Similar results were found for describing (higher impact factor: OR, 1.83; 95% CI, 1.27 to 2.62; more recent publication year: OR, 1.29; 95% CI, 1.08 to 1.55) as well as for discussing (higher impact factor: OR, 1.72; 95% CI, 1.16 to 2.55; more recent publication year: OR, 1.55; 95% CI, 1.17 to 2.06). Additionally, the probability of describing the previous article was inversely associated with the concordance of results (OR, 0.38; 95% CI, 0.17 to 0.88) and the probability of being discussed was increased for previous articles that employed meta-analytic methods (OR, 32.36; 95% CI, 2.00 to 522.85). The AMSTAR score of the recent meta-analysis as well as the similarity score were not significantly associated with any of the outcomes (see Table 3) and the nominal variables journal title and medical discipline were excluded from the regression analysis.

**Table 3:** **Results of the regression analysis**

**Study characteristic**	**OR (95% CI)**	***P*** **-value**
***Cited***		
**Impact factor**	**1.49 (1.06 to 2.10)**	**0.021**
**Publication year**	**1.19 (1.03 to 1.37)**	**0.018**
Concordance of results	0.92 (0.46 to 1.86)	0.814
Meta-analytic method used	1.41 (0.45 to 4.46)	0.549
Similarity of the review question	0.99 (0.94 to 1.05)	0.789
AMSTAR score	1.10 (0.79 to 1.53)	0.569
***Described***		
**Impact factor**	**1.83 (1.27 to 2.62)**	**0.001**
**Publication year**	**1.29 (1.08 to 1.55)**	**0.006**
**Concordance of results**	**0.38 (0.17 to 0.88)**	**0.023**
Meta-analytic method used	3.42 (0.82 to 14.34)	0.092
Similarity of the review question	1.01 (0.94 to 1.08)	0.757
AMSTAR score	1.00 (0.99 to 1.00)	0.136
***Discussed***		
**Impact factor**	**1.72 (1.16 to 2.55)**	**0.007**
**Publication year**	**1.55 (1.17 to 2.06)**	**0.002**
Concordance of results	0.40 (0.14 to 1.12)	0.081
**Meta-analytic method used**	**32.36 (2.00 to 522.85)**	**0.014**
Similarity of the review question	0.92 (0.83 to 1.02)	0.116
AMSTAR score	0.76 (0.43 to 1.34)	0.345

Associations between characteristics of the previous studies and odds to be cited, described, and discussed by the recent meta-analyses (as well as the AMSTAR score of the recent articles). Statistically significant results (*P* ≤ 0.05) are highlighted in bold. CI, confidence interval; OR, odds ratio.

The inter-rater reliability for the independent raters was found to be Kappa = 0.664 (*P* < 0.001; 95% CI, 0.607 to 0.721).

## Discussion

We found that in recent meta-analyses on pharmacological interventions published in leading medical journals, the proportion citing, describing, or discussing previous meta-analyses and systematic reviews on the same topic was low. Specifically, we found that only two-thirds of previous meta-analyses and systematic reviews were cited, one-third described, and only one in five of the previous articles’ results was discussed in light of the recent meta-analysis’ findings.

For individual RCTs it has been pointed out that most new trials are not interpreted, planned, and designed in the context of existing systematic reviews and other relevant evidence [6,23]. Our findings suggest that this statement applies also to otherwise methodologically sound meta-analyses. A fundamental principle of meta-analyses and systematic reviews is that all relevant clinical trials should be considered. We believe that they should also outline previous meta-analyses and systematic reviews about the same topic. Understanding the existing literature is central to any new project. In case of a meta-analysis, not referring to the results of previous meta-analyses and systematic reviews is especially problematic because it is likely to lead to confusion and disinformation among clinicians, patients, and policymakers, which is exactly the opposite of what any effort aiming at synthesizing scientific findings should be.

One could argue that citing 65% of previous relevant meta-analyses and systematic reviews is not a bad result, but we believe that simply putting a reference to another review is not enough. Systematic reviewers should place their results in the context of previous reviews, that is, provide a meaningful comment, comparison, or explanation of existing differences.

Moreover, this neglect is an example of inadequate study planning, suggesting that many authors do not perform the necessary literature search before initiating their own project [7,24]. This might very well be one of the reasons behind unnecessary duplication of effort in health sciences [8]. As trenchantly expressed by Terry and colleagues, ‘The issue of knowing what research is currently being undertaken … is a black hole in the public health landscape’ [25]. Especially worrisome is the fact that the authors who refer to similar previous papers rarely justify why their own project was undertaken, given a similar work was recently performed. In our sample we found 10 recent meta-analyses referring to a very similar previous work (‘similarity score’ four out of four). Only six of them justified why the same analysis was performed again (most common reason being that the previous paper needs to be updated or that some discordant results require clarification). None of them mentioned rigorous replication as a reason, suggesting that an ‘efficient culture for replication of research’ [8] has yet to emerge in health sciences.

### Predictors

We found that this neglect to refer to previously published systematic reviews and meta-analyses was predicted by a number of variables. According to our model, previous meta-analyses and systematic reviews were more likely to be cited, described, or discussed by a recent meta-analyses if recently published in a journal with a high impact factor, if results were different, and if meta-analytic methods were used.

### Publication year

More-recent meta-analyses are simply more up to date and usually include more RCTs. However, this does not necessarily mean that an older meta-analysis should be neglected - depending, among other factors, on how many new studies have been published since, an older meta-analysis can still serve as a valuable source of information that should be included in the literature review. Importantly, when we excluded all the previous articles published before 2010, the general pattern of our main result did not change (see Figure 1), showing that neglecting to refer to and discuss previously published systematic reviews and meta-analyses persisted even for the most recent material.

### Impact factor

Our results show that for an increase of one unit in impact factor the odds to get cited by a recent meta-analysis increased by 49%. Although criticized, impact factor is an important criterion for readers to assess the importance of scientific literature [26]. However, authors of systematic reviews should be especially careful not to miss important insights published outside high-impact-factor journals and select evidence on grounds of methodological validity rather than simply high visibility [27].

### Different results

We hypothesize that omitting similar findings from previous papers may constitute an (un)conscious strategy performed by authors to artificially create ‘novelty value’ to win an advantage during the peer-review and publication process. This is because journals demand novel, ground-breaking results to qualify for acceptance [28] - revealing that another article, using similar methodology, has obtained the same results likely decreases the novelty of the submitted paper. Such acts distort readers’ understanding of the true landscape of the medical evidence.

### Meta-analytic methods

Although it is generally acknowledged that meta-analysis can be an important and reliable source of information [29], we would like to emphasize that the methodology itself cannot be a synonym of scientific quality and authors should be aware of both strengths and weaknesses of this method [30].

### Limitations

Our analysis has limitations. We decided to focus only on clinical journals with the highest impact factors, because they usually publish papers of high scientific quality [31]. Nevertheless, our sample may not be considered representative for all medical meta-analyses. Because we wanted to be systematic in our approach, we included New England Journal of Medicine, although it does not publish many systematic reviews. We also restricted ourselves to pharmacological interventions. Therefore, our results do not necessarily generalize to other forms of treatment or other journals although we do not see any obvious reasons why the situation there should differ. We only used PubMed to identify the previous articles, so we might have missed some relevant meta-analyses or systematic reviews about a given topic. However, because we always included all previous meta-analyses and systematic reviews cited by the recent article (that is, all previous systematic reviews and meta-analyses that were on the reference list of a given recent article), our results represent a rather conservative estimate of the proportion of the previous meta-analyses and systematic reviews that were cited, described, and discussed by the recent meta-analyses. Selection by a single reviewer and 25% double extraction was also a limitation of our study, but the level of agreement between reviewers was good according to the Kappa coefficient [22]. Moreover, this is not a review where exact accuracy is essential - our primary result is very robust and our conclusions would not change even if the number of discussed and described papers should increase by a factor of two. Finally, our detailed description of the results in the Additional files allows verification and replication (see Additional file 7 for a list of references to all included meta-analyses and systematic reviews).

As we are not aware of any other research that could have guided our selection of predictors, we chose them based on our own expertise. Because of that, some of the measures we used have not been previously validated (similarity score of the review question, concordance of results), but we made sure they were as simple as possible and well-operationalized (including *a priori* definitions wherever possible). Moreover, the similarity score was based on the PICO questions that are considered essential in defining which studies to include and exclude [14] and constitute a well-recognized procedure [32].

## Conclusions and policy implications

Upcoming systematic reviews and meta-analyses should include an outline of previous systematic work about the same topic. Such an outline should be recommended by evidence-based medicine guidelines and officially implemented by the editorial policies. Currently, the Preferred Reporting Items for Systematic Reviews and Meta-Analyses (PRISMA) Statement recommends that authors of systematic reviews explain in the introduction how their work adds to what is already known and explain whether is it a new review or an update (see item 3: Rationale) [33]. This is not sufficient. In this respect the Consolidated Standards of Reporting Trials (CONSORT) Statement seems more demanding, recommending that each new trial includes a reference to a systematic review of previous similar trials or a note of the absence of such trials (see item 2a: Scientific background and explanation of rationale) [34]. We feel that there is no reason why systematic reviews should not follow an analogous procedure. The Cochrane Collaboration already acknowledged this problem and includes an obligatory section ‘Agreements and disagreements with other studies or reviews’ in its software Review Manager [35].

To reduce unnecessary duplication of research effort and adequately determine whether there is a need to undertake a new project, all systematic reviews and meta-analyses should be prospectively registered [8] using international registries of protocols, like PROSPERO [36].

Limiting the failure to refer to what is already known would make systematic reviews and meta-analyses a more useful, transparent, and valuable source of information for clinicians, researchers, policymakers, and patients. This simple step towards clarity and informativeness would enhance evidence-based practice as well as reduce waste in research resources [6-8,10,37] and reduce human suffering [38].

## Additional files

Additional file 1:
**PRISMA flow diagram for recent meta-analyses.**
Additional file 2:
**PRISMA flow diagrams for previous systematic reviews and meta-analyses.**
Additional file 3:
**Similarity score of the review question between recent and previous review: definitions and examples.**
Additional file 4:
**Concordance of results: example of similar and different results.**
Additional file 5:
**Summary characteristics of recent and previous studies.**
Additional file 6:
**Detailed characteristics of recent meta-analyses.**
Additional file 7:
**References to all included meta-analyses and systematic reviews.**

## Abbreviations

AMSTAR: A measurement tool for the assessment of the methodological quality of systematic reviews
CI: confidence interval
CONSORT: Consolidated Standards of Reporting Trials
OR: odds ratio
PICO: participants, intervention, comparator, outcome
PRISMA: Preferred Reporting Items for Systematic Reviews and Meta-Analyses
PROSPERO: international prospective register of systematic reviews
RCT: randomized controlled trial
