# Supplementary material for: Recent meta-analyses neglect previous systematic reviews and meta-analyses about the same topic: a systematic examination
Source: BMC Med. 2015 Apr 14;13:82. doi: 10.1186/s12916-015-0317-4 (PMC4411715; doi:10.1186/s12916-015-0317-4)

Additional file 1

PRISMA flow diagram for recent meta-analyses.

PubMed search term: "BMJ"[Journal] OR "JAMA"[Journal] OR "Plos med"[Journal] OR "Ann Intern Med"[Journal] OR "N Engl J Med"[Journal] OR "Lancet"[Journal] AND ("2012/01/01"[PDAT] : "2013/03/01"[PDAT]) AND (Review[ptyp] OR systematic[sb])

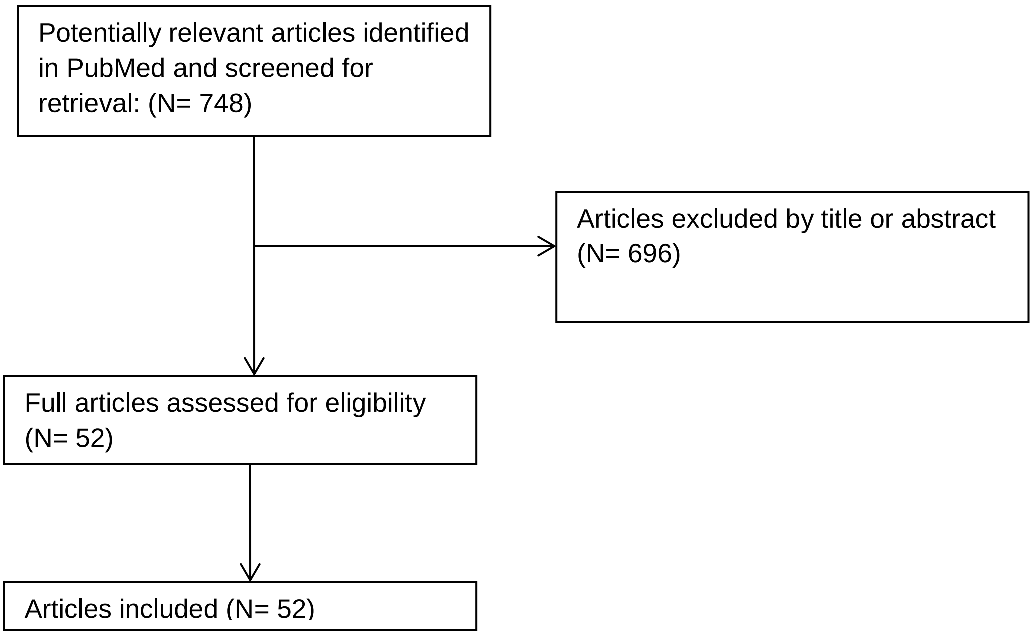

Supplement: Additional file 1: — PRISMA flow diagram for recent meta-analyses. [file 12916_2015_317_MOESM1_ESM.pdf]
