# Supplementary material for: Recent meta-analyses neglect previous systematic reviews and meta-analyses about the same topic: a systematic examination
Source: BMC Med. 2015 Apr 14;13:82. doi: 10.1186/s12916-015-0317-4 (PMC4411715; doi:10.1186/s12916-015-0317-4)

Additional file 2

PRISMA flow diagrams for previous systematic reviews and meta-analyses.

Makani[1] PubMed search term: "ACE inhibitors"[All Fields] OR "angiotensin receptor blockers"[All Fields]) OR "direct renin inhibitors"[All Fields] AND ((Meta-Analysis[ptyp] OR systematic[sb]) AND ("2003/01/28"[PDAT] : "2012/01/28"[PDAT])

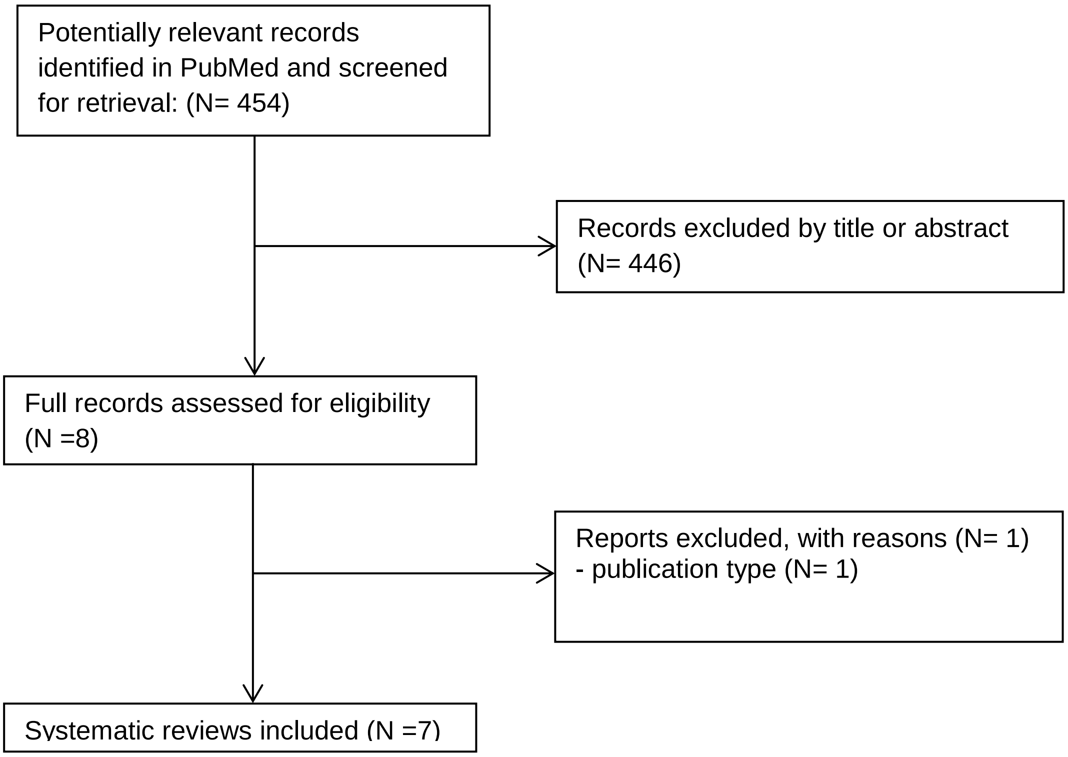

Stafford[9] PubMed search term: "("psychotic disorders"[MeSH Terms] OR ("psychotic"[All Fields] AND "disorders"[All Fields]) OR "psychotic disorders"[All Fields] OR "psychosis"[All Fields]) AND (high[All Fields] AND ("risk"[MeSH Terms] OR "risk"[All Fields])) AND ((systematic[sb] OR Meta-Analysis[ptyp]) AND ("2003/01/18"[PDAT] : "2012/01/18"[PDAT]))

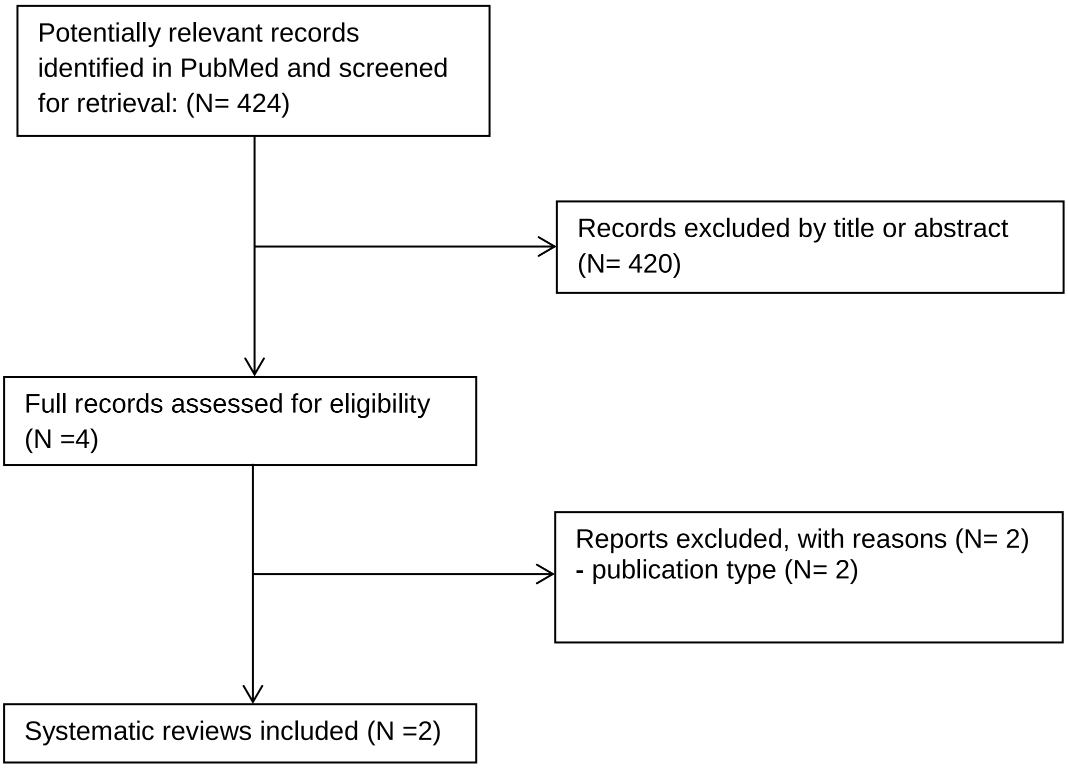

Chatterjee[12] PubMed search term: ("adrenergic beta-antagonists"[Pharmacological Action] OR "adrenergic beta-antagonists"[MeSH Terms] OR ("adrenergic"[All Fields] AND "beta-antagonists"[All Fields]) OR "adrenergic beta-antagonists"[All Fields] OR ("beta"[All Fields] AND "blockers"[All Fields]) OR "beta blockers"[All Fields]) AND ("heart failure"[MeSH Terms] OR ("heart"[All Fields] AND "failure"[All Fields]) OR "heart failure"[All Fields])) AND (systematic[sb] OR Meta-Analysis[ptyp]) AND ("2003/01/16"[PDAT] : "2012/01/16"[PDAT])

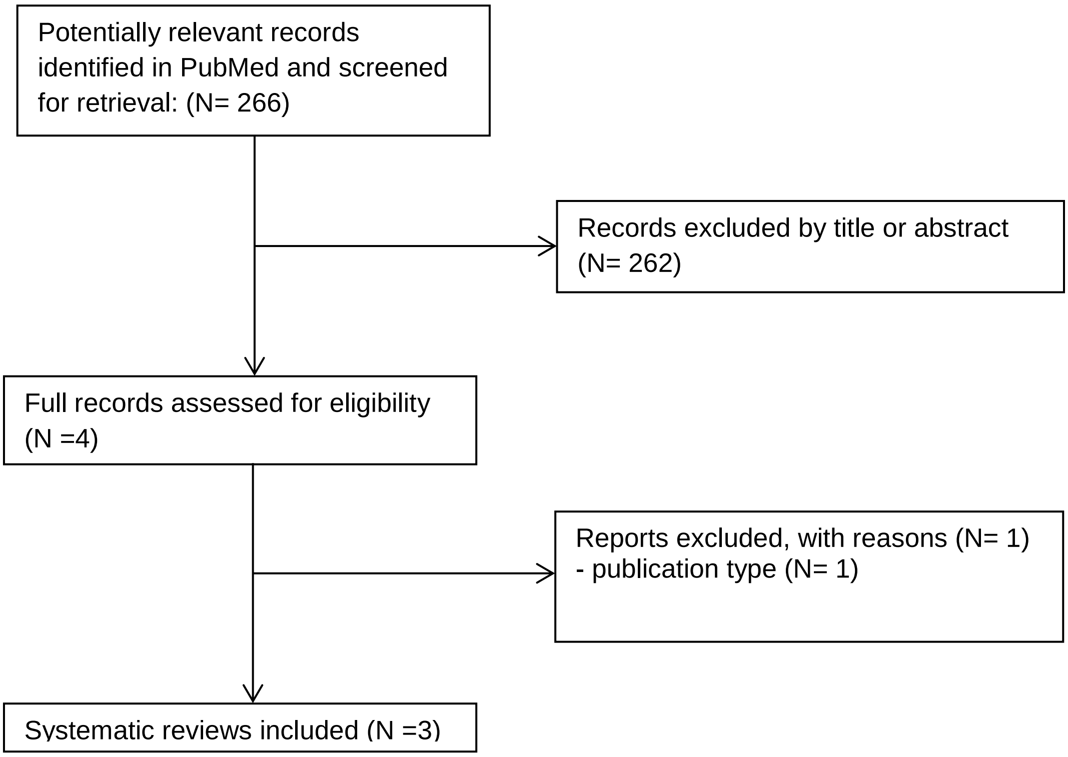

Haase[16] PubMed search term: ((("hydroxyethyl starch derivatives"[MeSH Terms] OR ("hydroxyethyl"[All Fields] AND "starch"[All Fields] AND "derivatives"[All Fields]) OR "hydroxyethyl starch derivatives"[All Fields] OR ("hydroxyethyl"[All Fields] AND "starch"[All Fields]) OR "hydroxyethyl starch"[All Fields]) OR ("crystalloid solutions"[Supplementary Concept] OR "crystalloid solutions"[All Fields] OR "crystalloid"[All Fields])) OR ("albumins"[MeSH Terms] OR "albumins"[All Fields] OR "albumin"[All Fields])) AND ("sepsis"[MeSH Terms] OR "sepsis"[All Fields]) AND ((systematic[sb] OR Meta-Analysis[ptyp]) AND ("2003/02/15"[PDAT] : "2012/02/15 "[PDAT]))

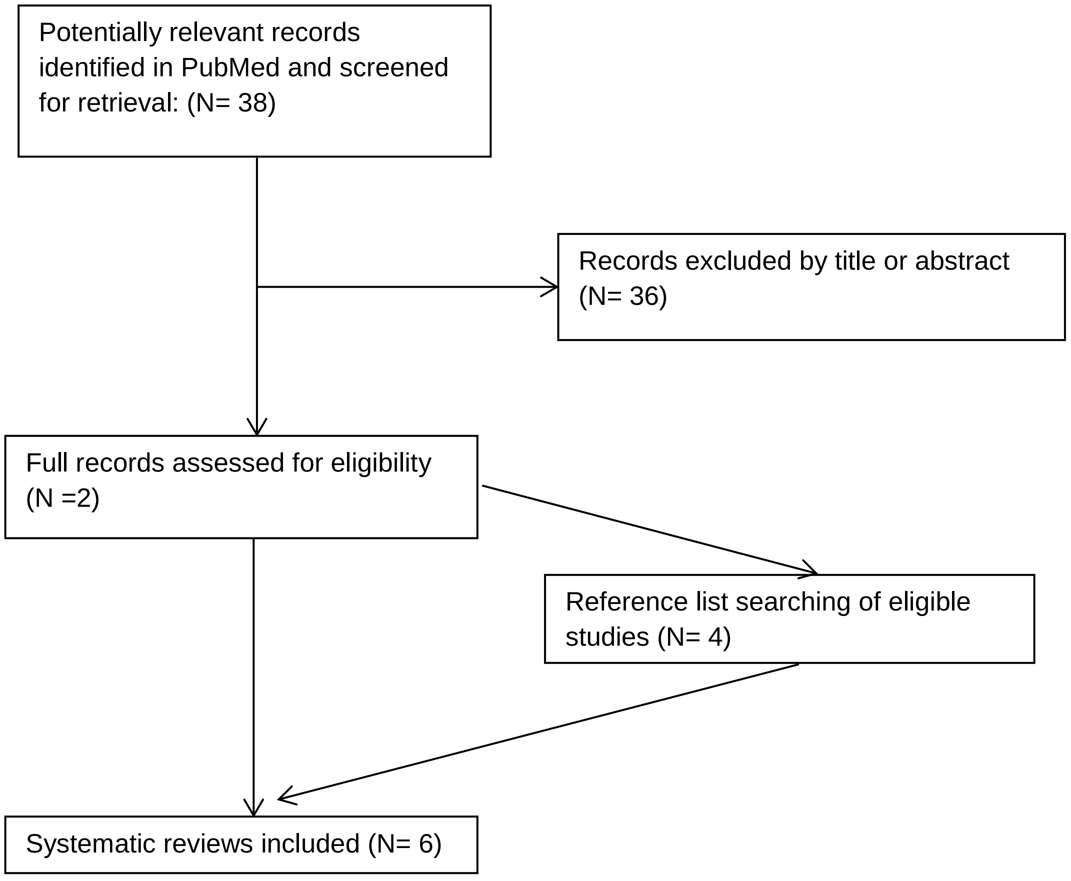

BellemainAppaix[23] PubMed search term: ("clopidogrel"[Supplementary Concept] OR "clopidogrel"[All Fields]) AND ("percutaneous coronary intervention"[MeSH Terms] OR ("percutaneous"[All Fields] AND "coronary"[All Fields] AND "intervention"[All Fields]) OR "percutaneous coronary intervention"[All Fields]) AND ((systematic[sb] OR Meta-Analysis[ptyp]) AND ("2002/12/19"[PDAT] : "2011/12/19"[PDAT]))

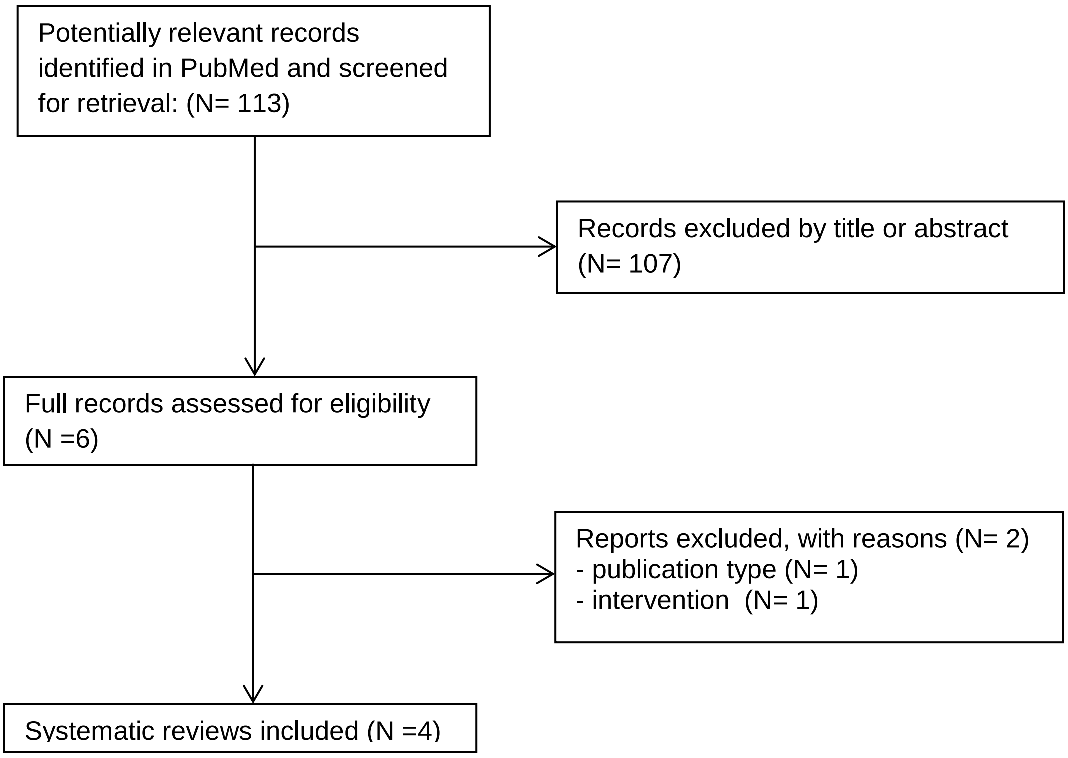

Johnston[28] PubMed search term: ("probiotics"[MeSH Terms] OR "probiotics"[All Fields] OR "probiotic"[All Fields]) AND ("clostridium difficile"[MeSH Terms] OR ("clostridium"[All Fields] AND "difficile"[All Fields]) OR "clostridium difficile"[All Fields]) AND ((systematic[sb] OR Meta-Analysis[ptyp]) AND ("2002/11/13"[PDAT] : "2011/11/13"[PDAT]))

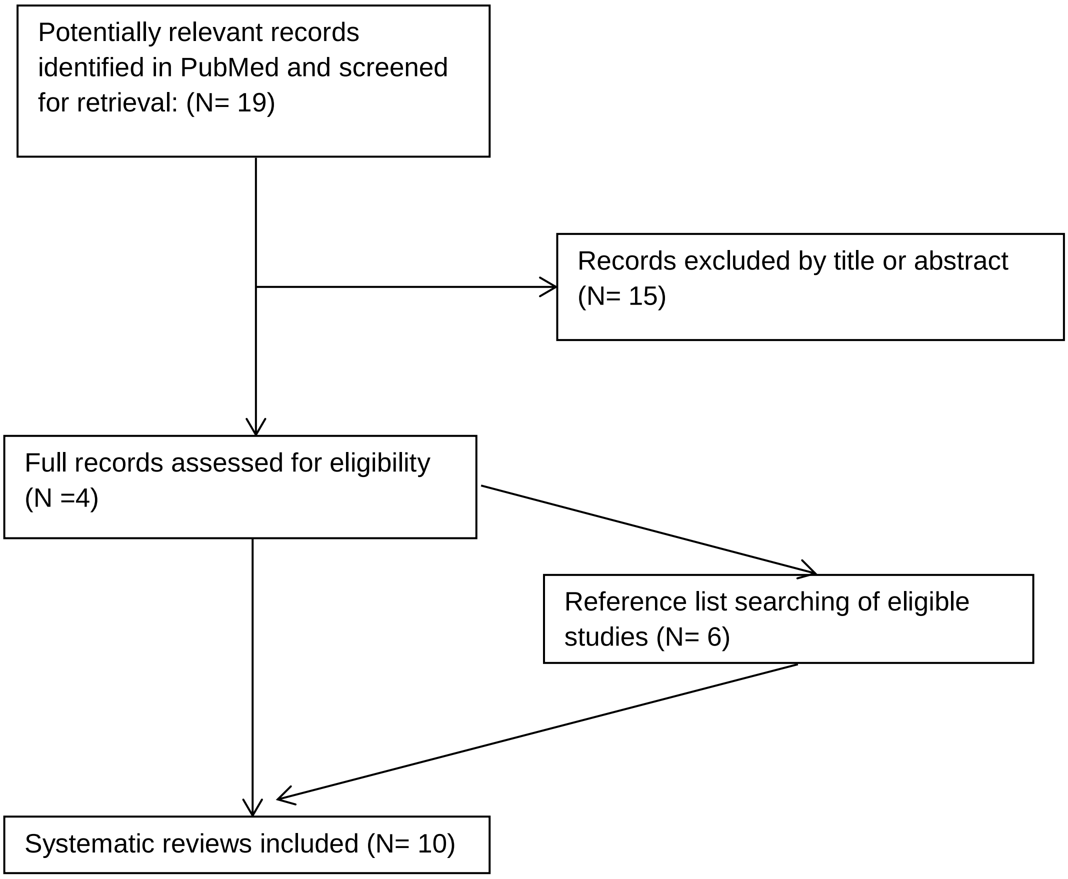

Pinto[39] PubMed search term: ("sciatica"[MeSH Terms] OR "sciatica"[All Fields]) AND (epidural[All Fields] AND ("adrenal cortex hormones"[Pharmacological Action] OR "adrenal cortex hormones"[MeSH Terms] OR ("adrenal"[All Fields] AND "cortex"[All Fields] AND "hormones"[All Fields]) OR "adrenal cortex hormones"[All Fields] OR "corticosteroids"[All Fields])) AND ((systematic[sb] OR Meta-Analysis[ptyp]) AND ("2002/11/13"[PDAT] : "2011/11/13"[PDAT]))

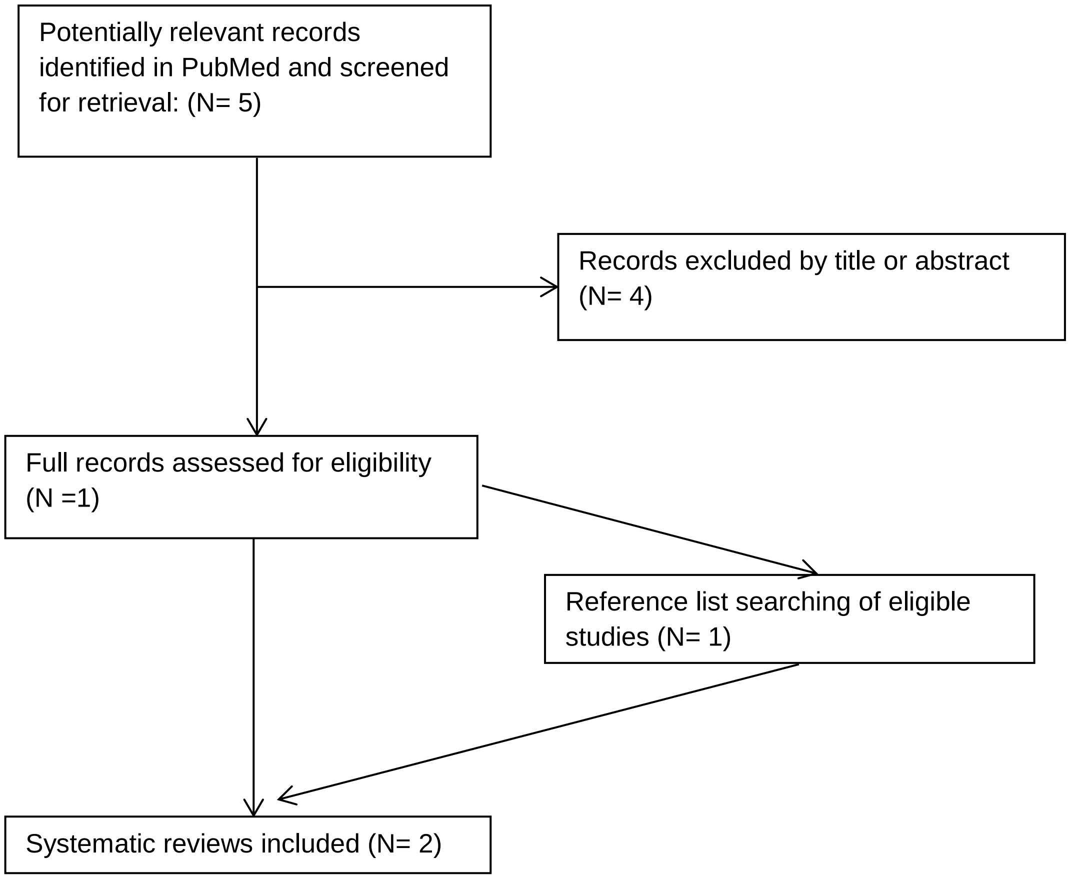

Huedo-Medina[42] PubMed search term: ("hypnotics and sedatives"[Pharmacological Action] OR "hypnotics and sedatives"[MeSH Terms] OR ("hypnotics"[All Fields] AND "sedatives"[All Fields]) OR "hypnotics and sedatives"[All Fields] OR "hypnotic"[All Fields]) AND ("sleep initiation and maintenance disorders"[MeSH Terms] OR ("sleep"[All Fields] AND "initiation"[All Fields] AND "maintenance"[All Fields] AND "disorders"[All Fields]) OR "sleep initiation and maintenance disorders"[All Fields] OR "insomnia"[All Fields]) AND ((systematic[sb] OR Meta-Analysis[ptyp]) AND ("2002/12/17"[PDAT] : "2011/12/17"[PDAT]))

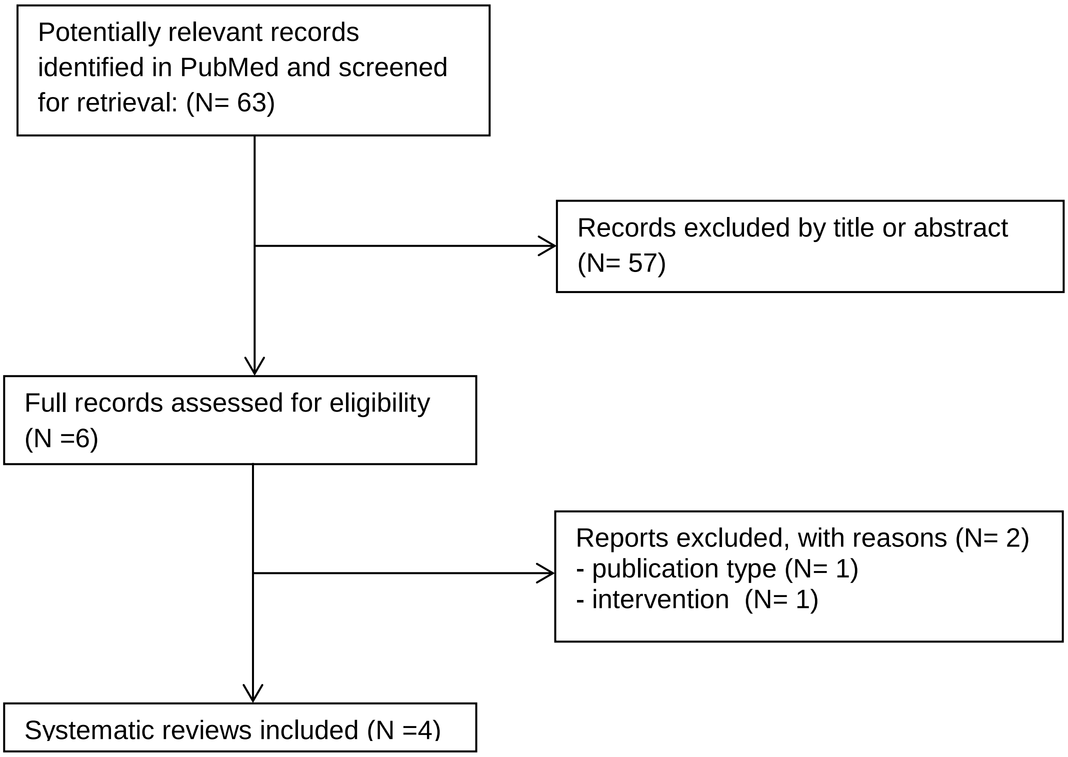

Fox[47] PubMed search term: (((("rivaroxaban"[Supplementary Concept] OR "rivaroxaban"[All Fields]) OR ("dabigatran"[Supplementary Concept] OR "dabigatran"[All Fields])) OR ("ximelagatran"[Supplementary Concept] OR "ximelagatran"[All Fields])) OR ("apixaban"[Supplementary Concept] OR "apixaban"[All Fields])) AND ("venous thromboembolism"[MeSH Terms] OR ("venous"[All Fields] AND "thromboembolism"[All Fields]) OR "venous thromboembolism"[All Fields]) AND ((systematic[sb] OR Meta-Analysis[ptyp]) AND ("2002/11/13"[PDAT] : "2011/11/13"[PDAT]))

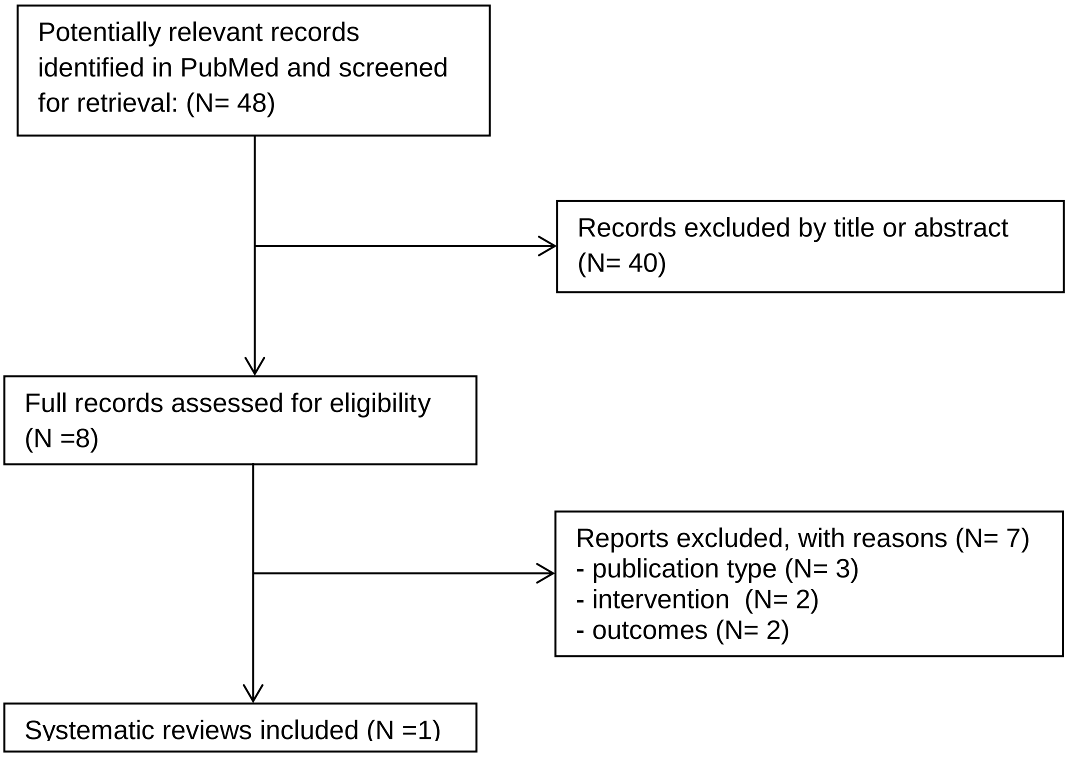

Low[49] PubMed search term: ("acetazolamide"[MeSH Terms] OR "acetazolamide"[All Fields]) AND ("altitude sickness"[MeSH Terms] OR ("altitude"[All Fields] AND "sickness"[All Fields]) OR "altitude sickness"[All Fields] OR ("mountain"[All Fields] AND "sickness"[All Fields]) OR "mountain sickness"[All Fields]) AND ((systematic[sb] OR Meta-Analysis[ptyp]) AND ("2002/10/18"[PDAT] : "2011/10/18"[PDAT]))

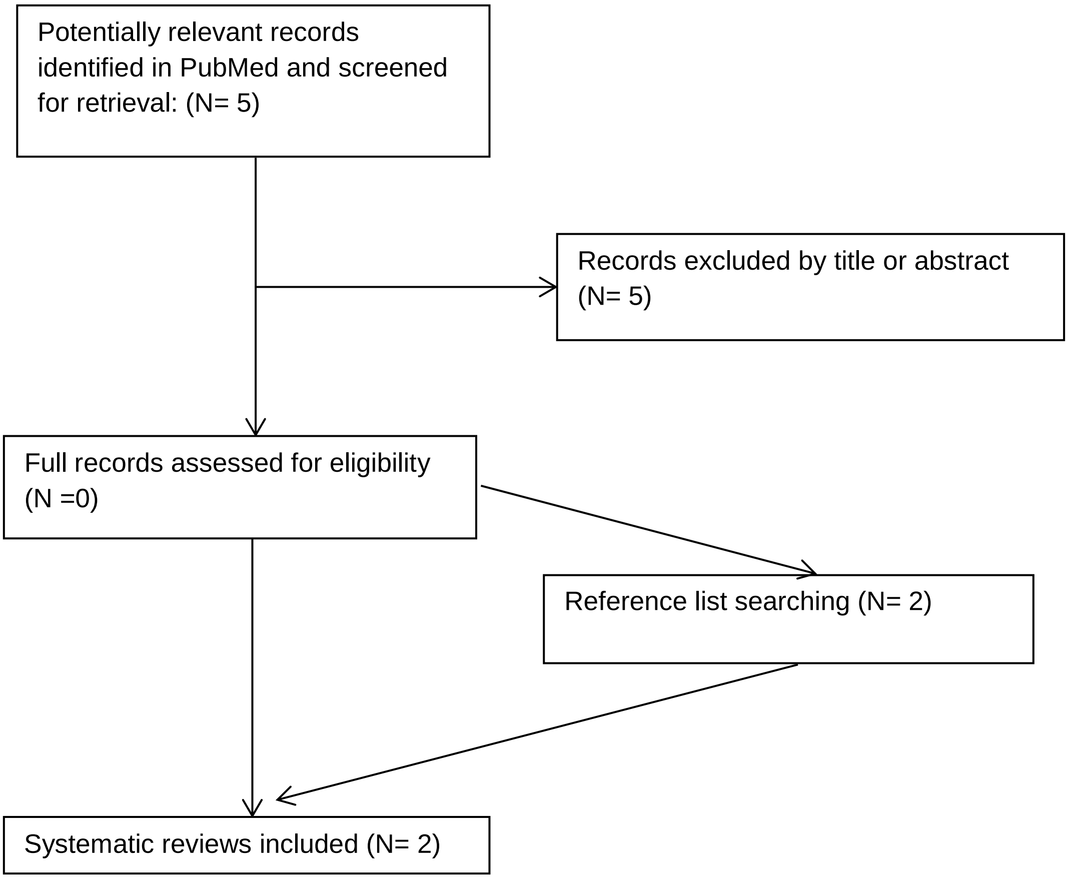

Zarychanski[52] PubMed search term: ("hydroxyethyl starch derivatives"[MeSH Terms] OR ("hydroxyethyl"[All Fields] AND "starch"[All Fields] AND "derivatives"[All Fields]) OR "hydroxyethyl starch derivatives"[All Fields] OR ("hydroxyethyl"[All Fields] AND "starch"[All Fields]) OR "hydroxyethyl starch"[All Fields]) AND ("critical illness"[MeSH Terms] OR ("critical"[All Fields] AND "illness"[All Fields]) OR "critical illness"[All Fields] OR ("critically"[All Fields] AND "ill"[All Fields]) OR "critically ill"[All Fields]) AND ((systematic[sb] OR Meta-Analysis[ptyp]) AND ("2003/02/20"[PDAT] : "2012/02/20"[PDAT]))

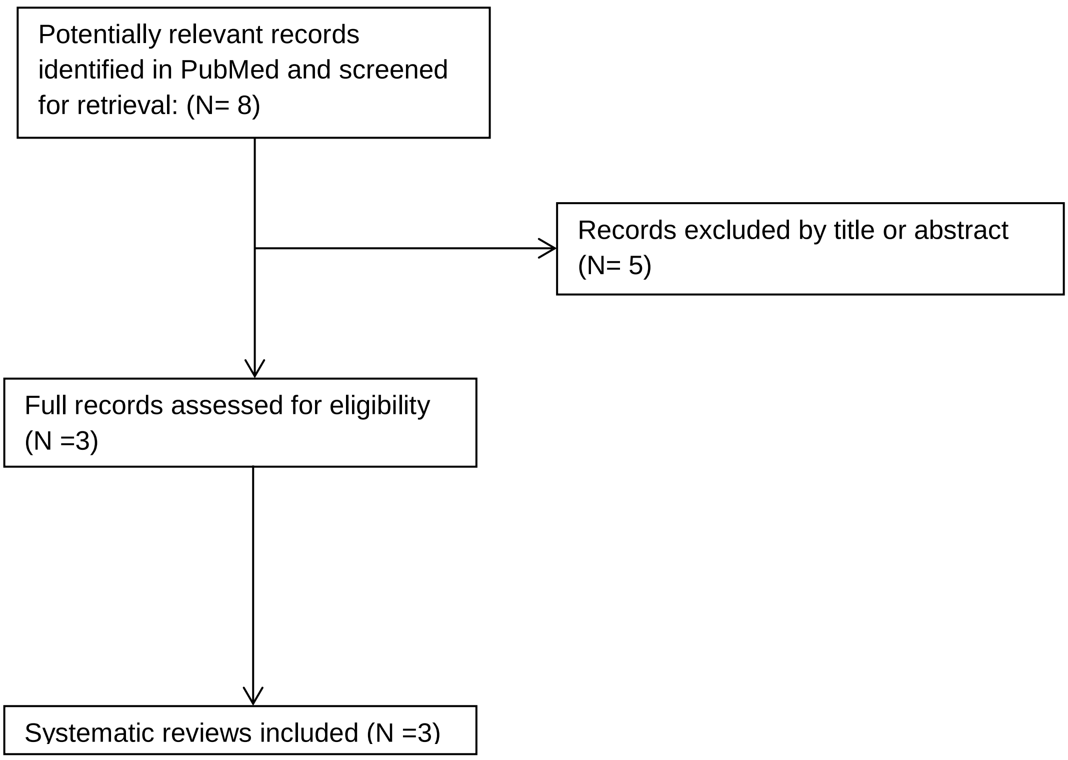

Bischoff-Ferrari[54] PubMed search term: ("vitamin d"[MeSH Terms] OR "vitamin d"[All Fields] OR "ergocalciferols"[MeSH Terms] OR "ergocalciferols"[All Fields]) AND (("fractures, bone"[MeSH Terms] OR ("fractures"[All Fields] AND "bone"[All Fields]) OR "bone fractures"[All Fields] OR "fracture"[All Fields]) AND ("prevention and control"[Subheading] OR ("prevention"[All Fields] AND "control"[All Fields]) OR "prevention and control"[All Fields] OR "prevention"[All Fields])) AND ((systematic[sb] OR Meta-Analysis[ptyp]) AND ("2002/07/05"[PDAT] : "2011/07/05"[PDAT]))

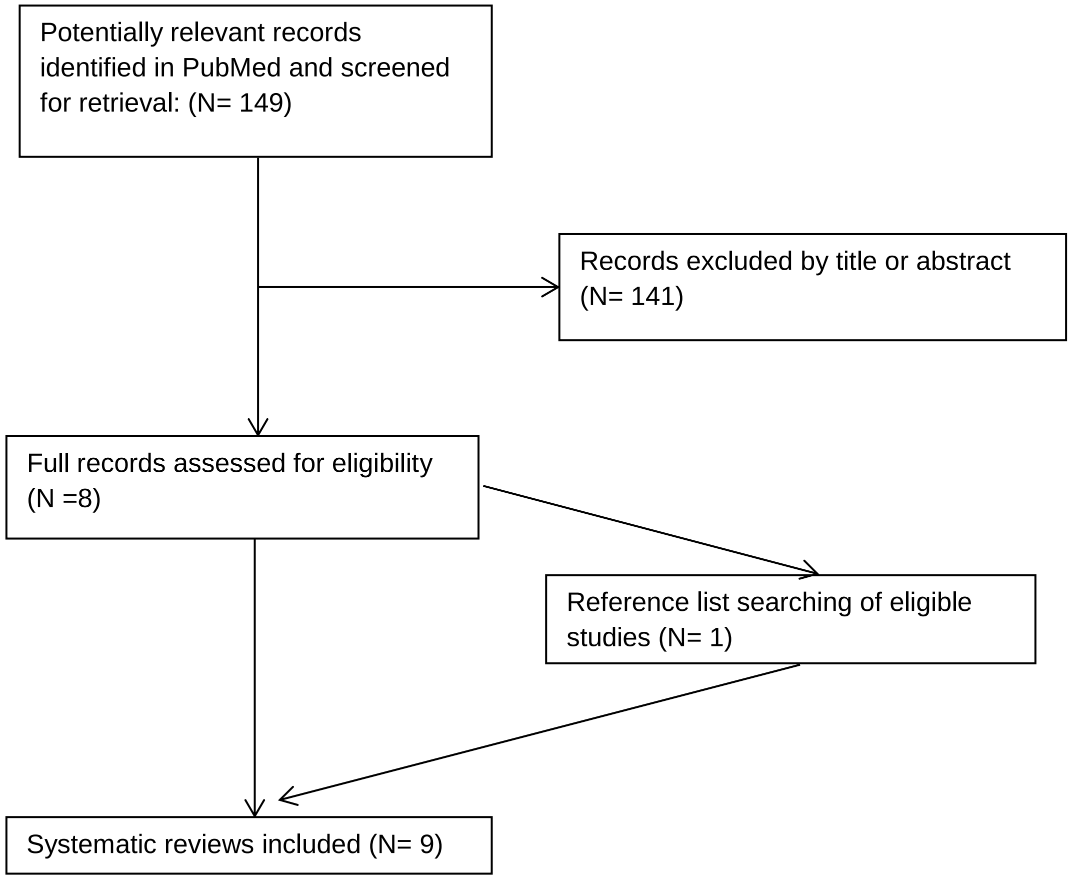

Haas[64] PubMed search term: ("tocolysis"[MeSH Terms] OR "tocolysis"[All Fields] OR ("tocolytic"[All Fields] AND "therapy"[All Fields]) OR "tocolytic therapy"[All Fields]) AND ("premature birth"[MeSH Terms] OR ("premature"[All Fields] AND "birth"[All Fields]) OR "premature birth"[All Fields] OR ("preterm"[All Fields] AND "delivery"[All Fields]) OR "preterm delivery"[All Fields]) AND ((systematic[sb] OR Meta-Analysis[ptyp]) AND ("2002/10/09"[PDAT] : "2011/10/09"[PDAT]))

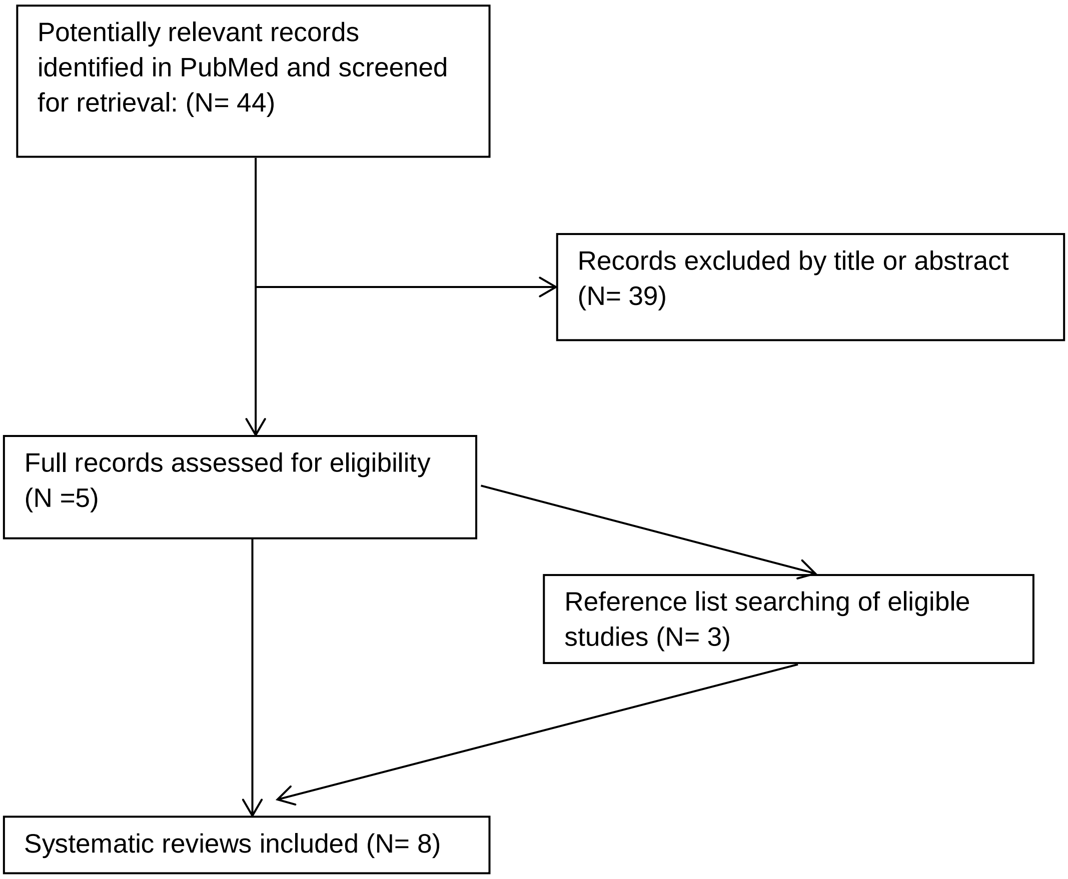

MacArthur[76] PubMed search term: (opiate[All Fields] AND ("therapy"[Subheading] OR "therapy"[All Fields] OR "treatment"[All Fields] OR "therapeutics"[MeSH Terms] OR "therapeutics"[All Fields])) OR (("analgesics, opioid"[Pharmacological Action] OR "analgesics, opioid"[MeSH Terms] OR ("analgesics"[All Fields] AND "opioid"[All Fields]) OR "opioid analgesics"[All Fields] OR "opioid"[All Fields]) AND ("therapy"[Subheading] OR "therapy"[All Fields] OR "treatment"[All Fields] OR "therapeutics"[MeSH Terms] OR "therapeutics"[All Fields])) AND ("hiv"[MeSH Terms] OR "hiv"[All Fields]) AND ((systematic[sb] OR Meta-Analysis[ptyp]) AND ("2002/10/04"[PDAT] : "2011/10/04"[PDAT]))

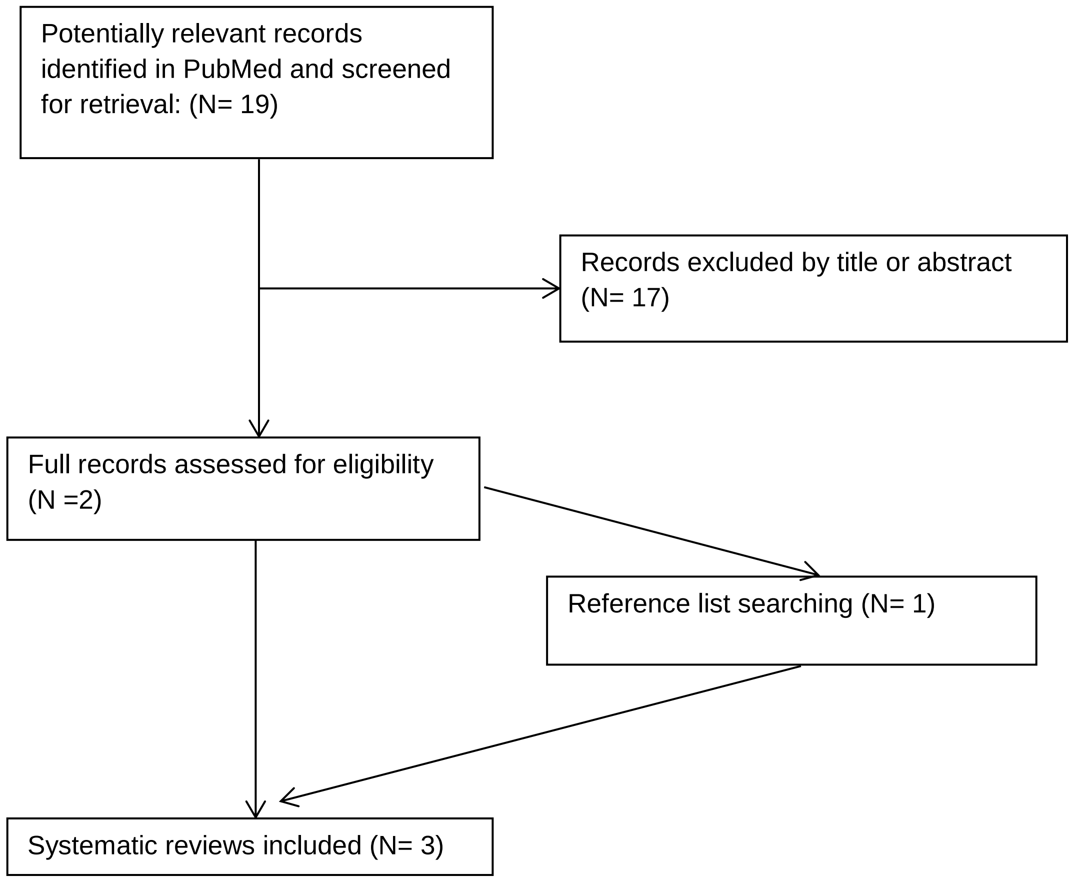

Hartling[80] PubMed search term: ("antipsychotic agents"[Pharmacological Action] OR "antipsychotic agents"[MeSH Terms] OR ("antipsychotic"[All Fields] AND "agents"[All Fields]) OR "antipsychotic agents"[All Fields] OR "antipsychotics"[All Fields]) AND ("schizophrenia"[MeSH Terms] OR "schizophrenia"[All Fields]) AND ((systematic[sb] OR Meta-Analysis[ptyp]) AND ("2002/06/14"[PDAT] : "2011/06/14"[PDAT]))

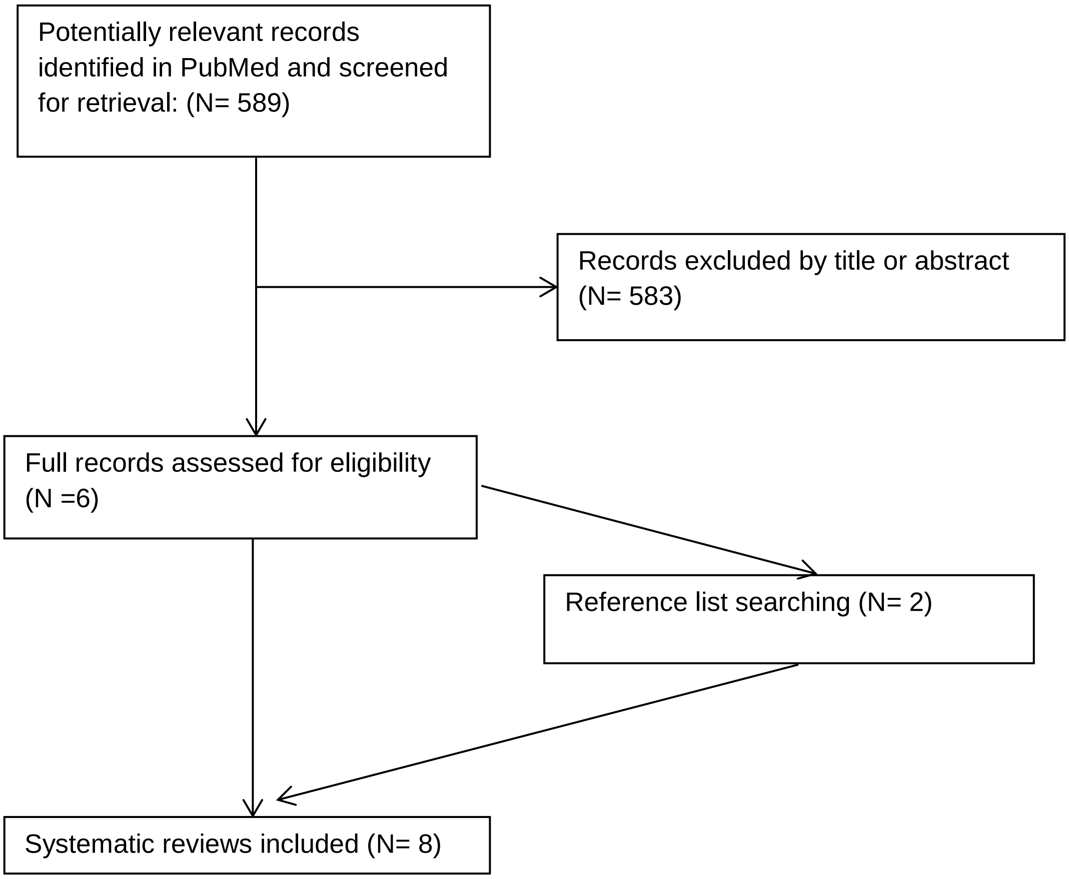

Hutton[89] PubMed search term: ("antifibrinolytic agents"[Pharmacological Action] OR "antifibrinolytic agents"[MeSH Terms] OR ("antifibrinolytic"[All Fields] AND "agents"[All Fields]) OR "antifibrinolytic agents"[All Fields] OR "antifibrinolytics"[All Fields]) AND ("thoracic surgery"[MeSH Terms] OR ("thoracic"[All Fields] AND "surgery"[All Fields]) OR "thoracic surgery"[All Fields] OR ("cardiac"[All Fields] AND "surgery"[All Fields]) OR "cardiac surgery"[All Fields] OR "cardiac surgical procedures"[MeSH Terms] OR ("cardiac"[All Fields] AND "surgical"[All Fields] AND "procedures"[All Fields]) OR "cardiac surgical procedures"[All Fields] OR ("cardiac"[All Fields] AND "surgery"[All Fields])) AND ((systematic[sb] OR Meta-Analysis[ptyp]) AND ("2002/09/11"[PDAT] : "2011/09/11"[PDAT]))

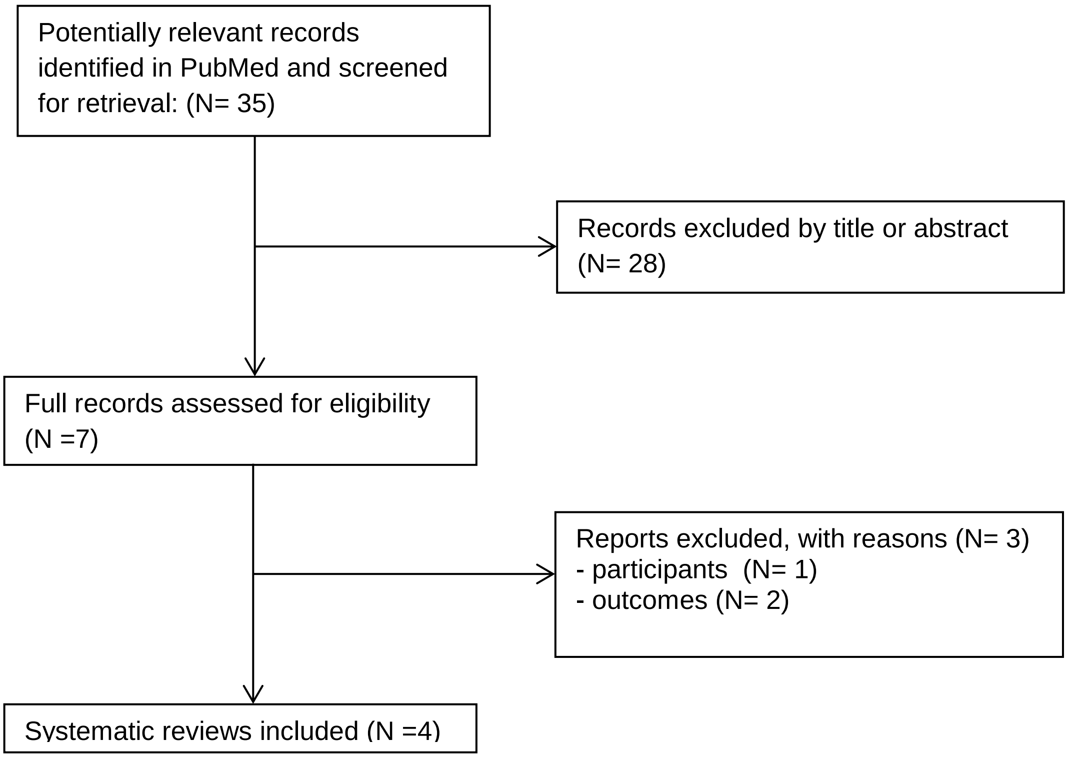

Lopez-Olivo[94] PubMed search term: ("neoplasms"[MeSH Terms] OR "neoplasms"[All Fields] OR "malignancies"[All Fields]) AND ("arthritis, rheumatoid"[MeSH Terms] OR ("arthritis"[All Fields] AND "rheumatoid"[All Fields]) OR "rheumatoid arthritis"[All Fields] OR ("rheumatoid"[All Fields] AND "arthritis"[All Fields])) AND ((systematic[sb] OR Meta-Analysis[ptyp]) AND ("2002/09/05"[PDAT] : "2011/09/05"[PDAT]))

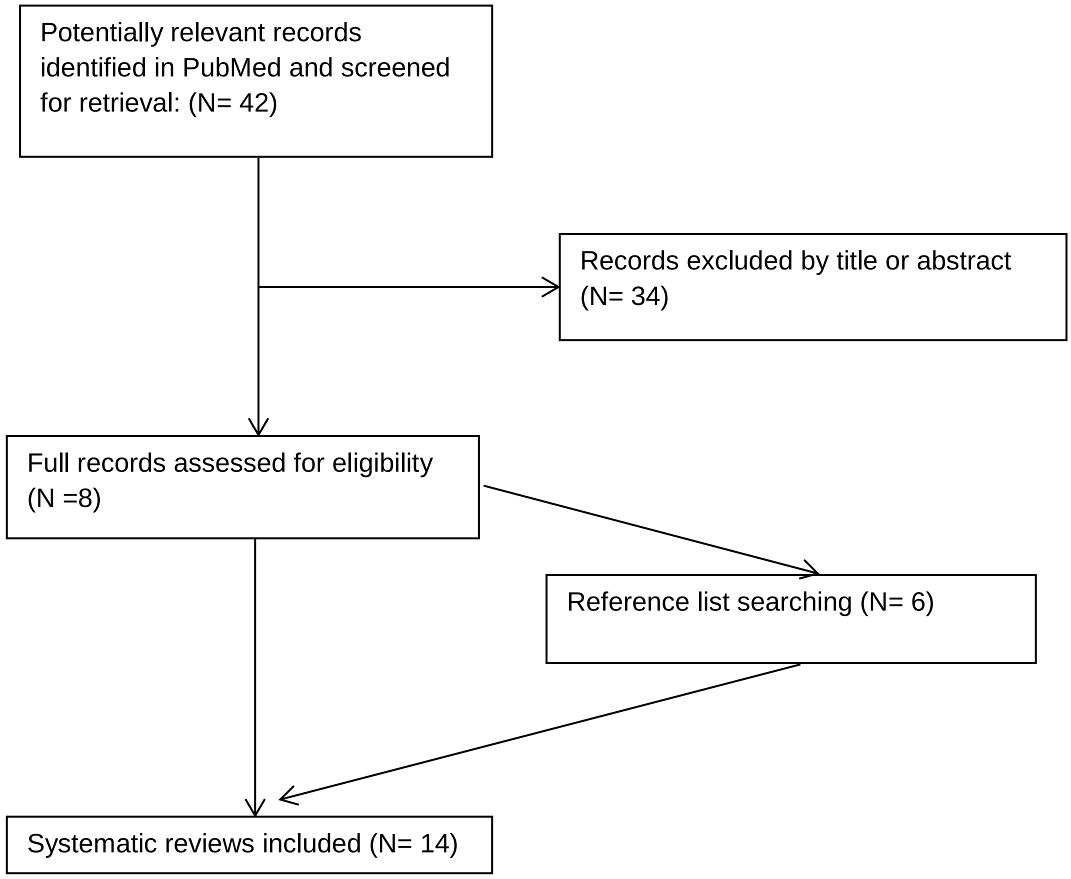

Rahimi[109] PubMed search term: (venous thromboemboli[All Fields] OR venous thromboembolic[All Fields] OR venous thromboembolism[All Fields] OR venous thromboembolisms[All Fields] OR venous thromboembolus[All Fields]) AND ("hydroxymethylglutaryl-coa reductase inhibitors"[Pharmacological Action] OR "hydroxymethylglutaryl-coa reductase inhibitors"[MeSH Terms] OR ("hydroxymethylglutaryl-coa"[All Fields] AND "reductase"[All Fields] AND "inhibitors"[All Fields]) OR "hydroxymethylglutaryl-coa reductase inhibitors"[All Fields] OR "statins"[All Fields]) AND ((systematic[sb] OR Meta-Analysis[ptyp]) AND ("2002/09/18"[PDAT] : "2011/09/18"[PDAT]))

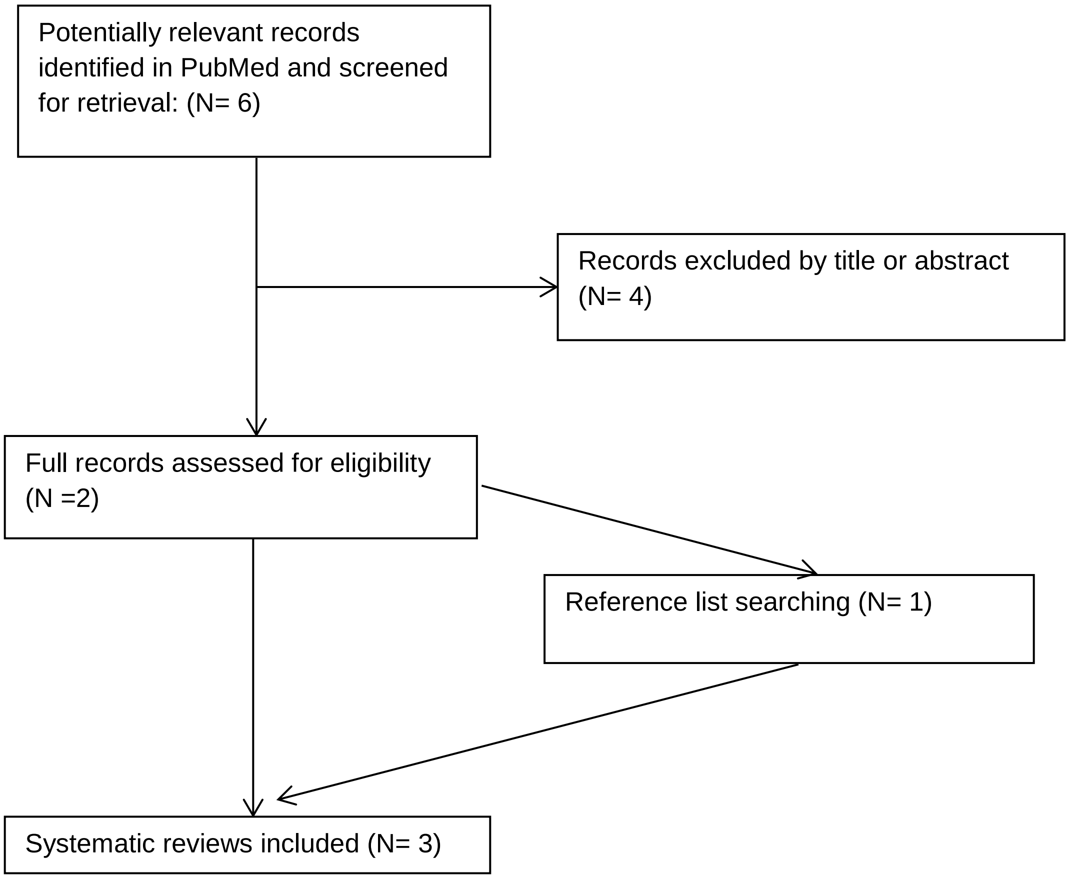

Plante[113] PubMed search term: ("steroids"[MeSH Terms] OR "steroids"[All Fields]) AND ("tonsillectomy"[MeSH Terms] OR "tonsillectomy"[All Fields]) AND ((systematic[sb] OR Meta-Analysis[ptyp]) AND ("2002/09/18"[PDAT] : "2011/09/18"[PDAT]))

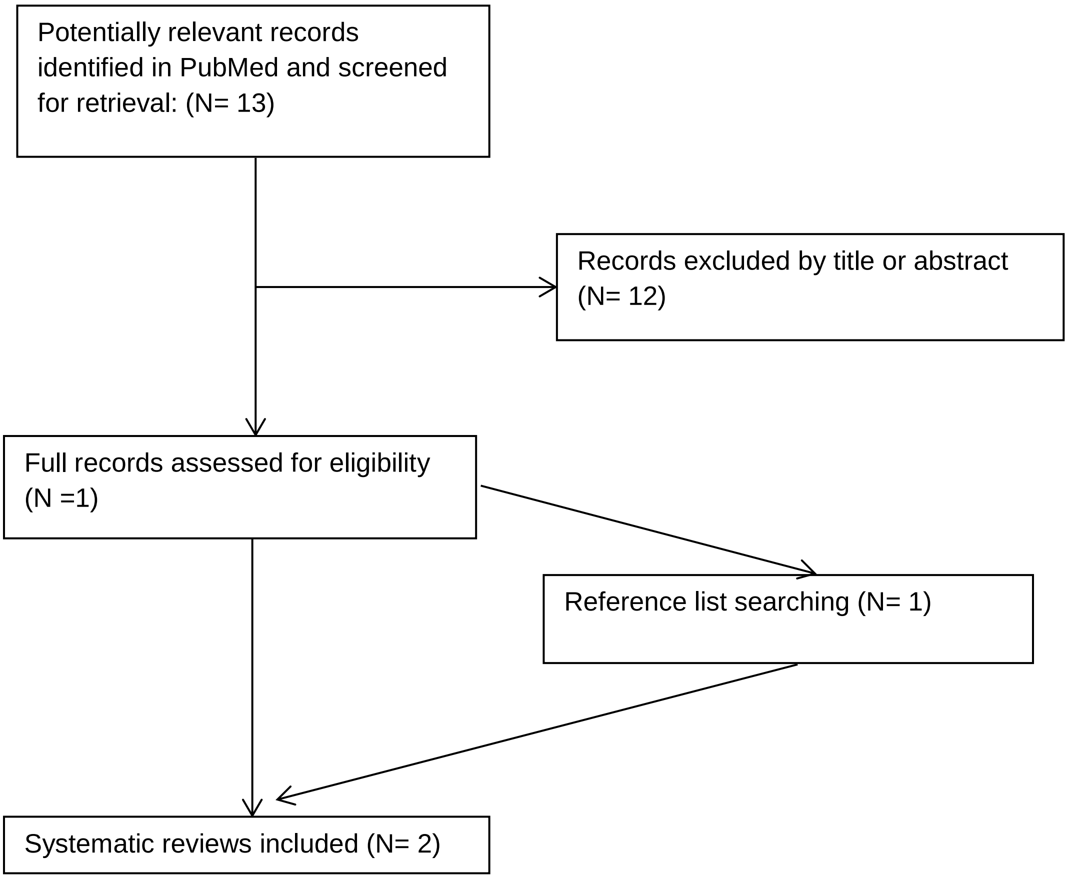

Preiss[116] PubMed search term: ("hydroxymethylglutaryl-coa reductase inhibitors"[Pharmacological Action] OR "hydroxymethylglutaryl-coa reductase inhibitors"[MeSH Terms] OR ("hydroxymethylglutaryl-coa"[All Fields] AND "reductase"[All Fields] AND "inhibitors"[All Fields]) OR "hydroxymethylglutaryl-coa reductase inhibitors"[All Fields] OR "statins"[All Fields]) AND ("fibric acids"[MeSH Terms] OR ("fibric"[All Fields] AND "acids"[All Fields]) OR "fibric acids"[All Fields] OR "fibrates"[All Fields]) AND ("pancreatitis"[MeSH Terms] OR "pancreatitis"[All Fields]) AND ((systematic[sb] OR Meta-Analysis[ptyp]) AND ("2002/08/22"[PDAT] : "2011/08/22"[PDAT]))

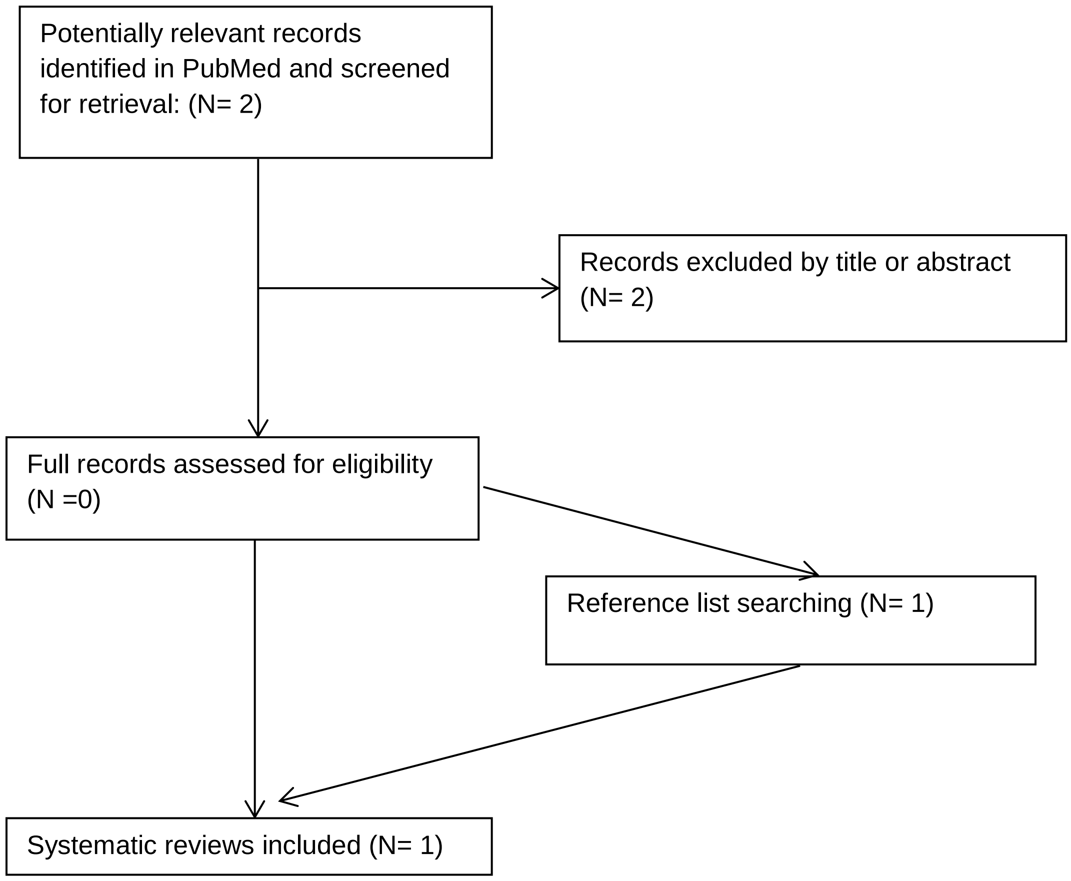

Palmer[118] PubMed search term: ("hydroxymethylglutaryl-coa reductase inhibitors"[Pharmacological Action] OR "hydroxymethylglutaryl-coa reductase inhibitors"[MeSH Terms] OR ("hydroxymethylglutaryl-coa"[All Fields] AND "reductase"[All Fields] AND "inhibitors"[All Fields]) OR "hydroxymethylglutaryl-coa reductase inhibitors"[All Fields] OR "statins"[All Fields]) AND (chronic[All Fields] AND ("kidney diseases"[MeSH Terms] OR ("kidney"[All Fields] AND "diseases"[All Fields]) OR "kidney diseases"[All Fields] OR ("kidney"[All Fields] AND "disease"[All Fields]) OR "kidney disease"[All Fields])) AND ((systematic[sb] OR Meta-Analysis[ptyp]) AND ("2002/08/22"[PDAT] : "2011/08/22"[PDAT]))

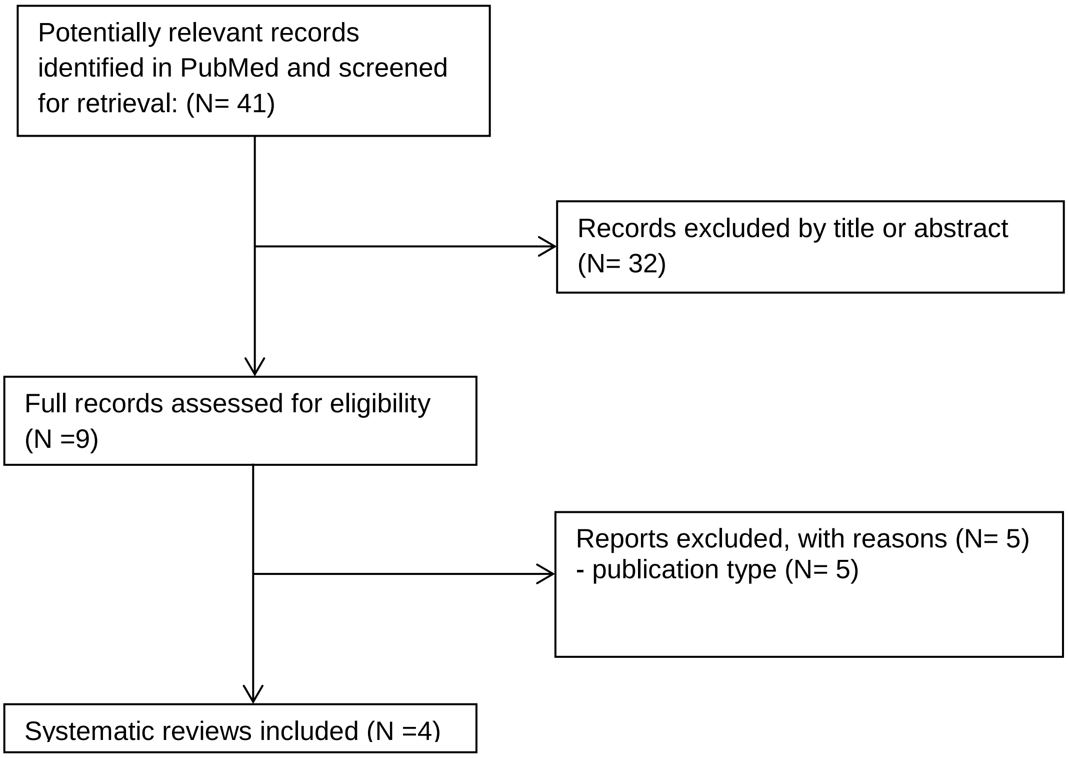

Upadhyay[123] PubMed search term: ("hydroxymethylglutaryl-coa reductase inhibitors"[Pharmacological Action] OR "hydroxymethylglutaryl-coa reductase inhibitors"[MeSH Terms] OR ("hydroxymethylglutaryl-coa"[All Fields] AND "reductase"[All Fields] AND "inhibitors"[All Fields]) OR "hydroxymethylglutaryl-coa reductase inhibitors"[All Fields] OR "statins"[All Fields]) AND (chronic[All Fields] AND ("kidney diseases"[MeSH Terms] OR ("kidney"[All Fields] AND "diseases"[All Fields]) OR "kidney diseases"[All Fields] OR ("kidney"[All Fields] AND "disease"[All Fields]) OR "kidney disease"[All Fields])) AND ((systematic[sb] OR Meta-Analysis[ptyp]) AND ("2002/08/21"[PDAT] : "2011/08/21"[PDAT]))

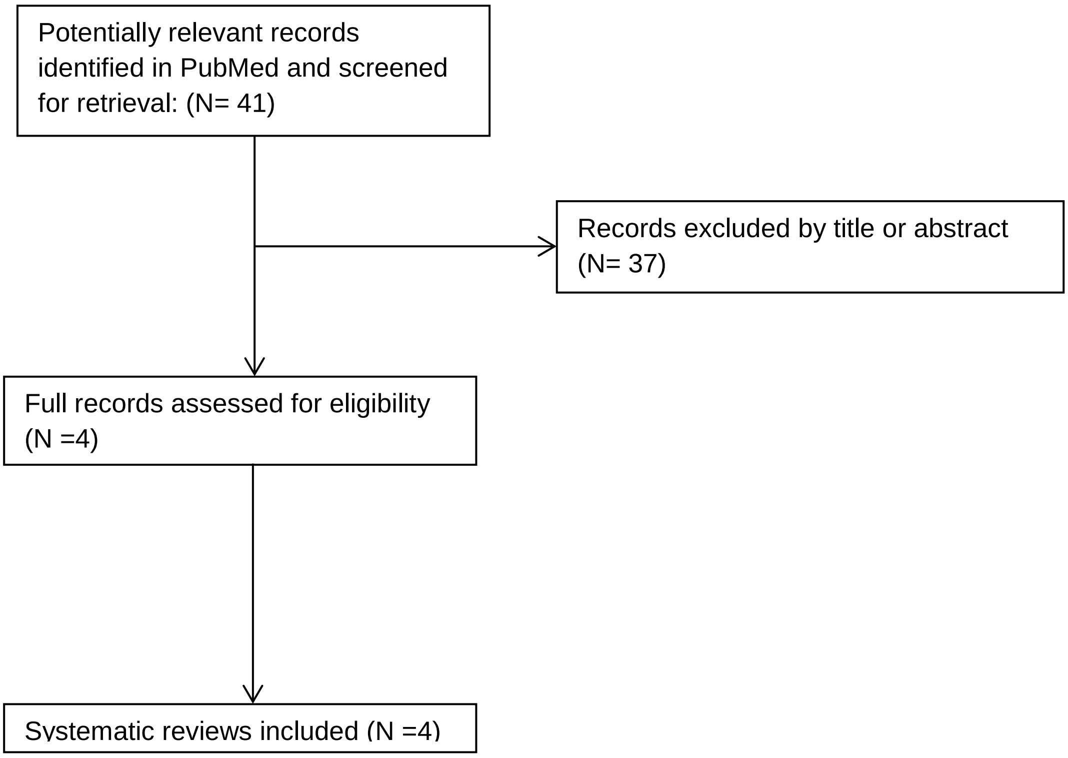

Mihaylova[124] PubMed search term: (("cholesterol, ldl"[MeSH Terms] OR ("cholesterol"[All Fields] AND "ldl"[All Fields]) OR "ldl cholesterol"[All Fields] OR ("ldl"[All Fields] AND "cholesterol"[All Fields])) AND ("hydroxymethylglutaryl-coa reductase inhibitors"[Pharmacological Action] OR "hydroxymethylglutaryl-coa reductase inhibitors"[MeSH Terms] OR ("hydroxymethylglutaryl-coa"[All Fields] AND "reductase"[All Fields] AND "inhibitors"[All Fields]) OR "hydroxymethylglutaryl-coa reductase inhibitors"[All Fields] OR "statins"[All Fields])) AND ("vascular diseases"[MeSH Terms] OR ("vascular"[All Fields] AND "diseases"[All Fields]) OR "vascular diseases"[All Fields] OR ("vascular"[All Fields] AND "disease"[All Fields]) OR "vascular disease"[All Fields]) AND ((systematic[sb] OR Meta-Analysis[ptyp]) AND ("2002/08/11"[PDAT] : "2011/08/11"[PDAT]))

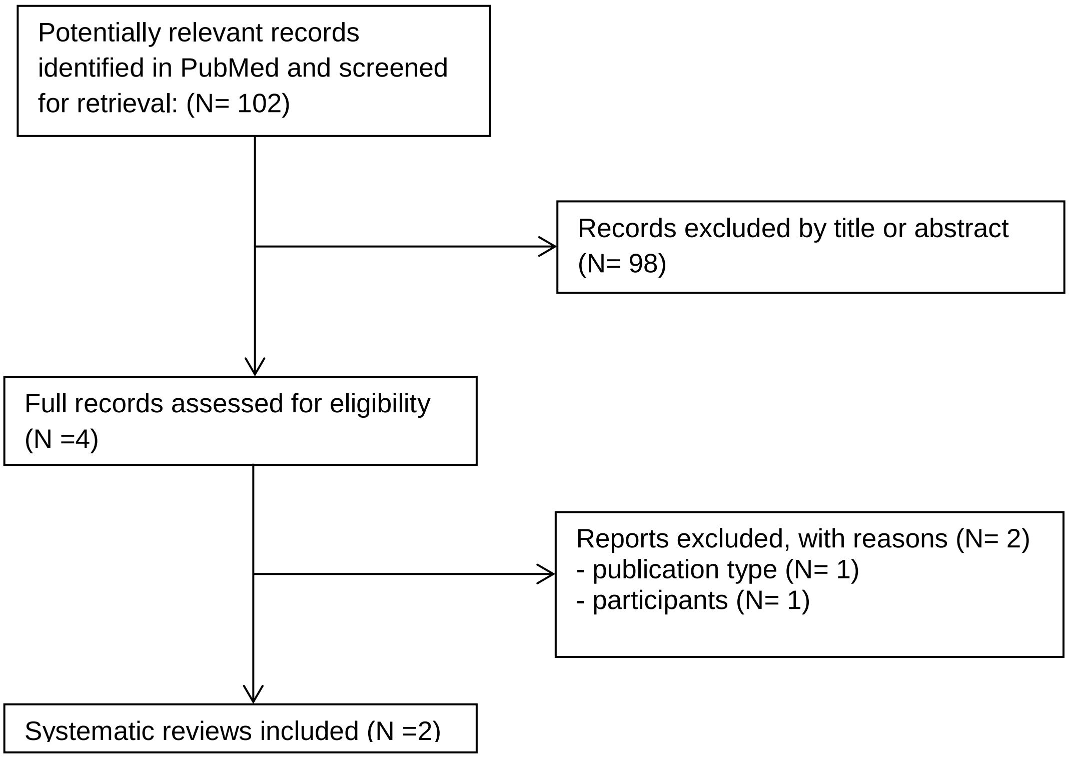

Bangalore[127] PubMed search term: (("drug-eluting stents"[MeSH Terms] OR ("drug-eluting"[All Fields] AND "stents"[All Fields]) OR "drug-eluting stents"[All Fields] OR ("drug"[All Fields] AND "eluting"[All Fields] AND "stents"[All Fields]) OR "drug eluting stents"[All Fields]) AND (bare[All Fields] AND ("metals"[MeSH Terms] OR "metals"[All Fields] OR "metal"[All Fields]) AND ("stents"[MeSH Terms] OR "stents"[All Fields]))) AND ("diabetes mellitus"[MeSH Terms] OR ("diabetes"[All Fields] AND "mellitus"[All Fields]) OR "diabetes mellitus"[All Fields] OR "diabetes"[All Fields] OR "diabetes insipidus"[MeSH Terms] OR ("diabetes"[All Fields] AND "insipidus"[All Fields]) OR "diabetes insipidus"[All Fields]) AND ((systematic[sb] OR Meta-Analysis[ptyp]) AND ("2002/08/10"[PDAT] : "2011/08/10"[PDAT]))

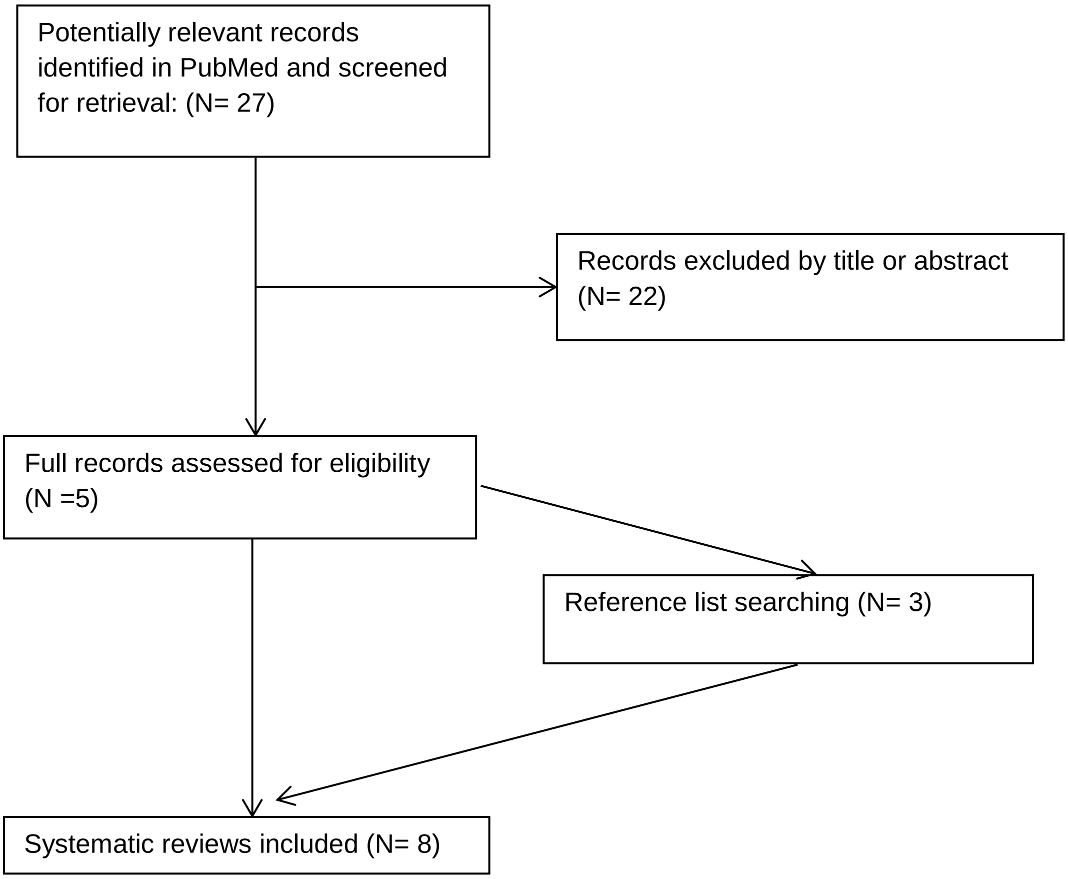

Mantha[136] PubMed search term: (venous thromboemboli[All Fields] OR venous thromboembolic[All Fields] OR venous thromboembolism[All Fields] OR venous thromboembolisms[All Fields]) AND (("progestins"[Pharmacological Action] OR "progestins"[MeSH Terms] OR "progestins"[All Fields] OR "progestin"[All Fields]) AND ("contraception"[MeSH Terms] OR "contraception"[All Fields])) AND ((systematic[sb] OR Meta-Analysis[ptyp]) AND ("2002/08/07"[PDAT] : "2011/08/07"[PDAT]))

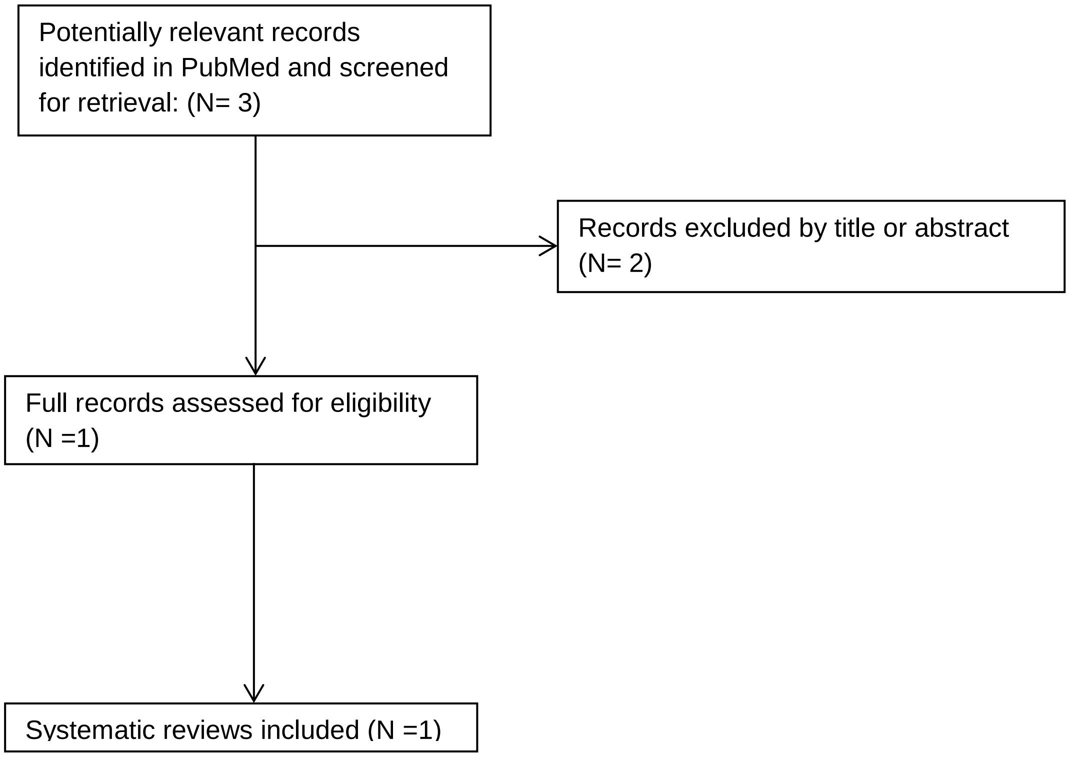

Rutjes[138] PubMed search term: ("viscosupplementation"[MeSH Terms] OR "viscosupplementation"[All Fields]) AND ("osteoarthritis"[MeSH Terms] OR "osteoarthritis"[All Fields]) AND ((systematic[sb] OR Meta-Analysis[ptyp]) AND ("2002/08/07"[PDAT] : "2011/08/07"[PDAT]))

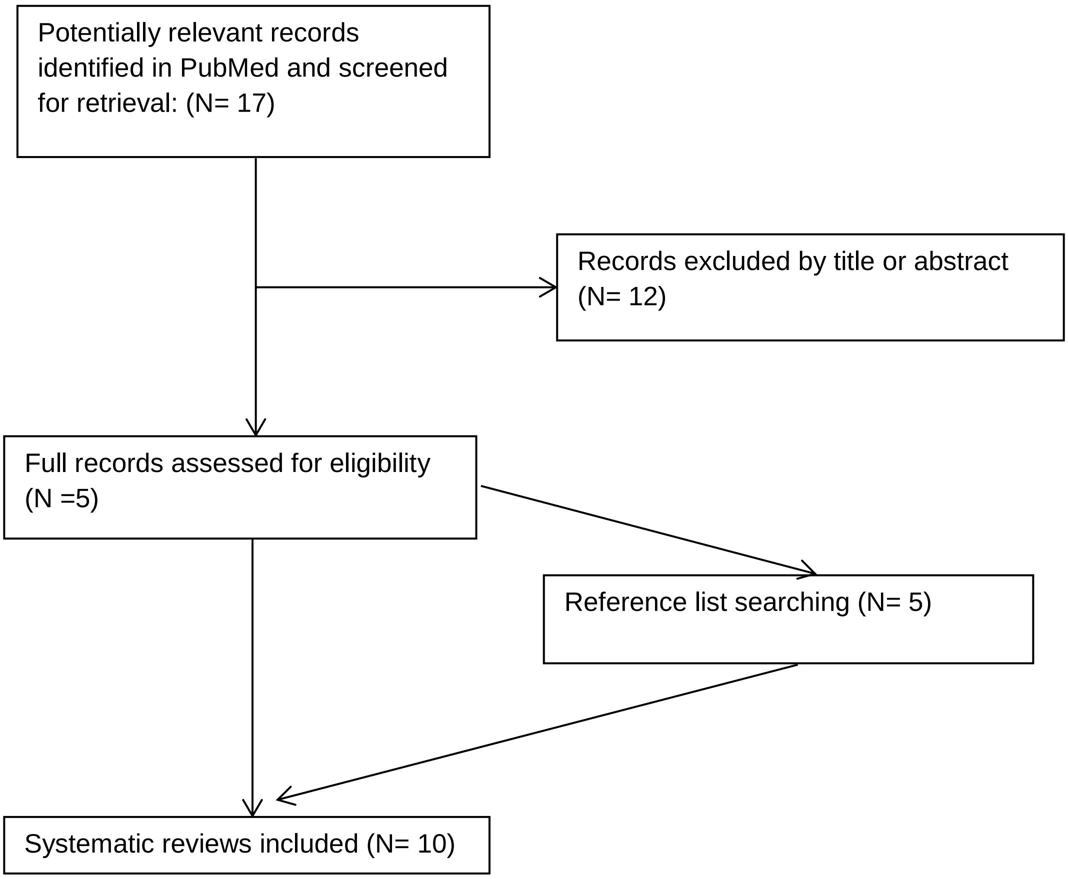

Ahuja[149] PubMed search term: ("tuberculosis, multidrug-resistant"[MeSH Terms] OR ("tuberculosis"[All Fields] AND "multidrug-resistant"[All Fields]) OR "multidrug-resistant tuberculosis"[All Fields] OR ("multidrug"[All Fields] AND "resistant"[All Fields] AND "tuberculosis"[All Fields]) OR "multidrug resistant tuberculosis"[All Fields]) AND ((systematic[sb] OR Meta-Analysis[ptyp]) AND ("2002/08/28"[PDAT] : "2011/08/28"[PDAT]))

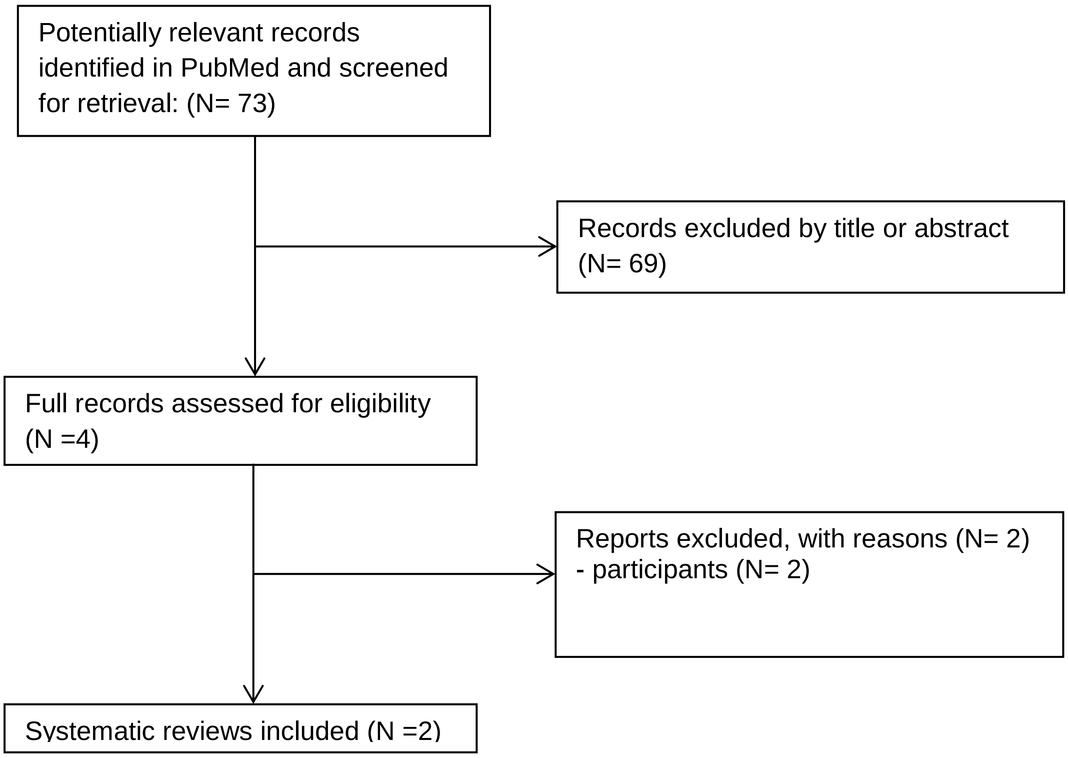

Lv[152] PubMed search term: (("blood pressure"[MeSH Terms] OR ("blood"[All Fields] AND "pressure"[All Fields]) OR "blood pressure"[All Fields] OR "blood pressure determination"[MeSH Terms] OR ("blood"[All Fields] AND "pressure"[All Fields] AND "determination"[All Fields]) OR "blood pressure determination"[All Fields] OR ("blood"[All Fields] AND "pressure"[All Fields]) OR "blood pressure"[All Fields] OR "arterial pressure"[MeSH Terms] OR ("arterial"[All Fields] AND "pressure"[All Fields]) OR "arterial pressure"[All Fields] OR ("blood"[All Fields] AND "pressure"[All Fields])) AND lowering[All Fields]) AND ((systematic[sb] OR Meta-Analysis[ptyp]) AND ("2002/08/21"[PDAT] : "2011/08/21"[PDAT]))

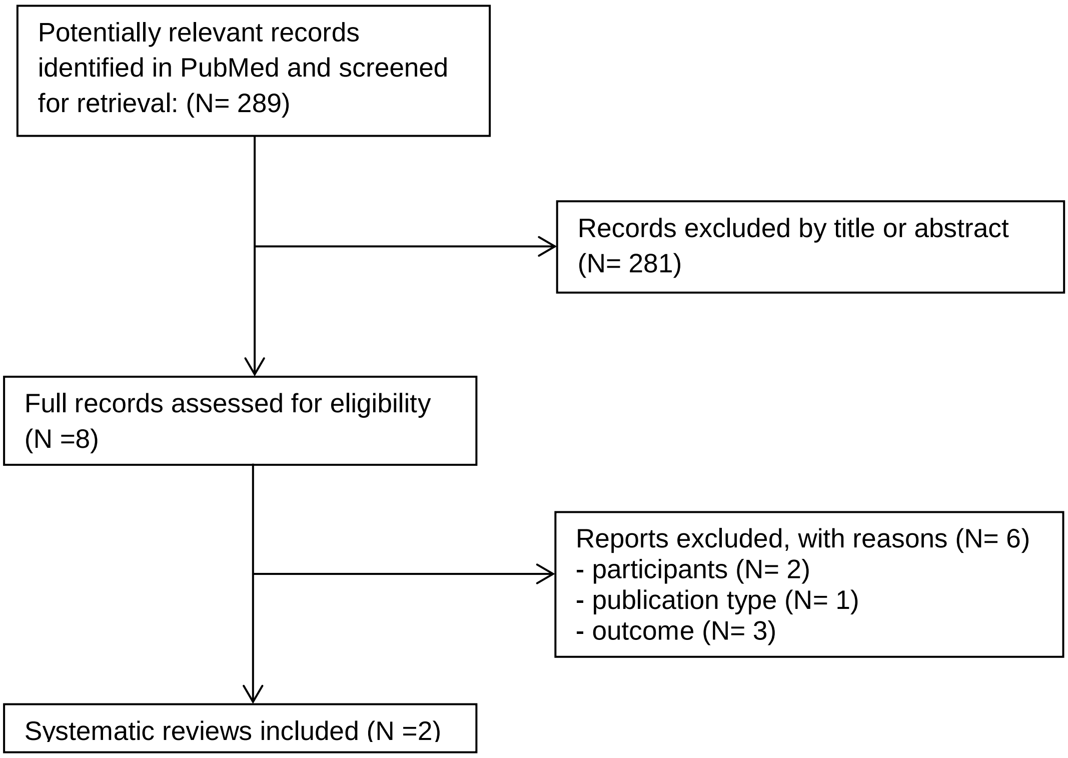

Karagiannis[155] PubMed search term: ("dipeptidyl peptidase 4"[MeSH Terms] OR "dipeptidyl peptidase 4"[All Fields]) AND ("diabetes mellitus"[MeSH Terms] OR ("diabetes"[All Fields] AND "mellitus"[All Fields]) OR "diabetes mellitus"[All Fields] OR "diabetes"[All Fields] OR "diabetes insipidus"[MeSH Terms] OR ("diabetes"[All Fields] AND "insipidus"[All Fields]) OR "diabetes insipidus"[All Fields]) AND ((systematic[sb] OR Meta-Analysis[ptyp]) AND ("2002/02/12"[PDAT] : "2011/02/12"[PDAT]))

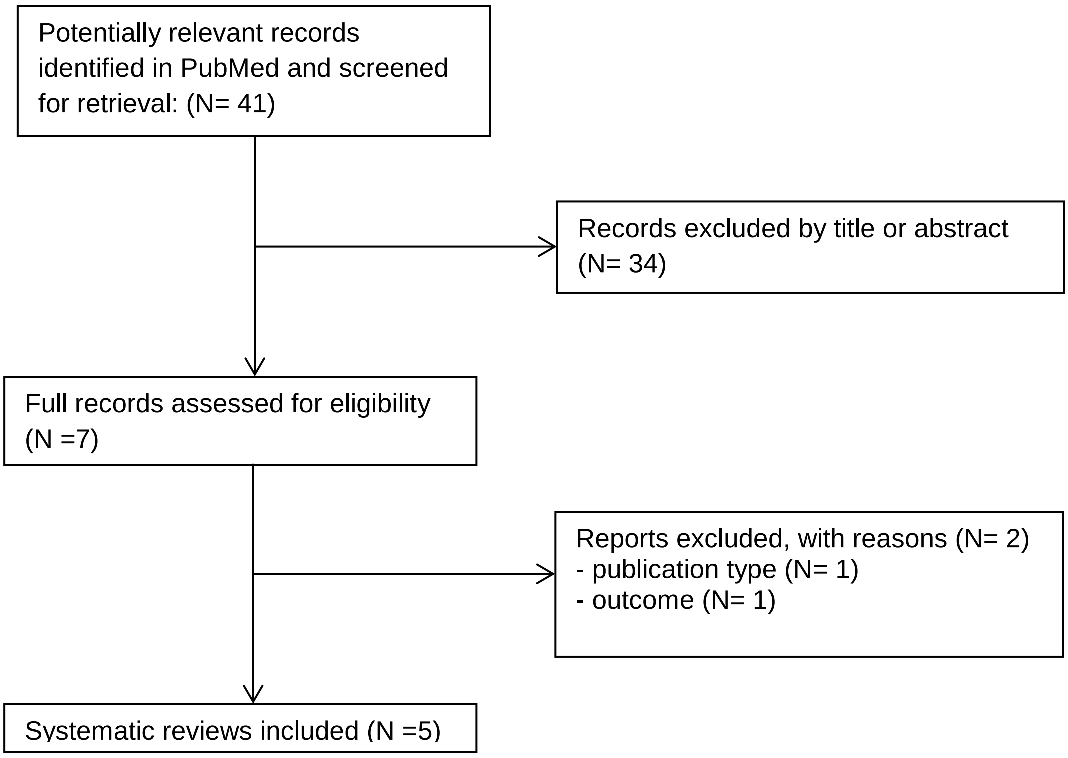

Caldeira[161] PubMed search term: ("pneumonia"[MeSH Terms] OR "pneumonia"[All Fields]) AND ("angiotensins"[MeSH Terms] OR "angiotensins"[All Fields] OR "angiotensin"[All Fields]) AND ((systematic[sb] OR Meta-Analysis[ptyp]) AND ("2002/07/11"[PDAT] : "2011/07/11"[PDAT]))

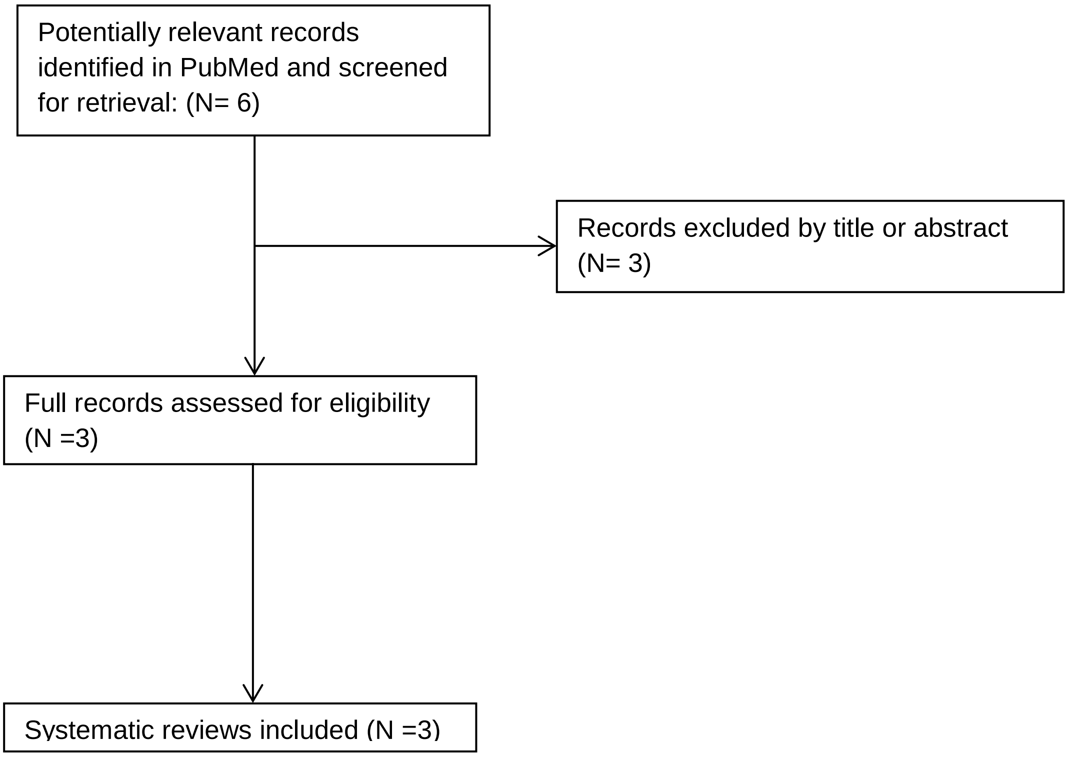

Suthar[165] PubMed search term: (antiretroviral[All Fields] AND ("tuberculosis"[MeSH Terms] OR "tuberculosis"[All Fields])) AND ("hiv"[MeSH Terms] OR "hiv"[All Fields]) AND ((systematic[sb] OR Meta-Analysis[ptyp]) AND ("2002/07/24"[PDAT] : "2011/07/24"[PDAT]))

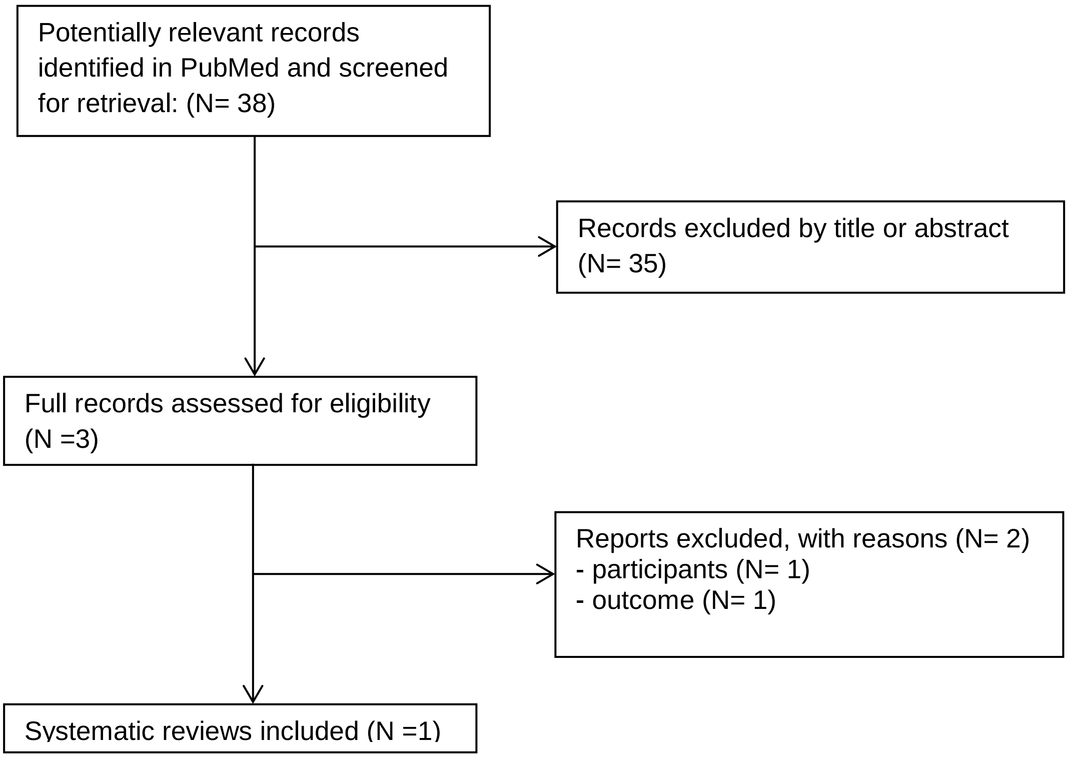

Wardlaw[167] PubMed search term: ("tissue plasminogen activator"[MeSH Terms] OR ("tissue"[All Fields] AND "plasminogen"[All Fields] AND "activator"[All Fields]) OR "tissue plasminogen activator"[All Fields] OR ("recombinant"[All Fields] AND "tissue"[All Fields] AND "plasminogen"[All Fields] AND "activator"[All Fields]) OR "recombinant tissue plasminogen activator"[All Fields]) AND ("stroke"[MeSH Terms] OR "stroke"[All Fields]) AND ((systematic[sb] OR Meta-Analysis[ptyp]) AND ("2002/06/23"[PDAT] : "2011/06/23"[PDAT]))

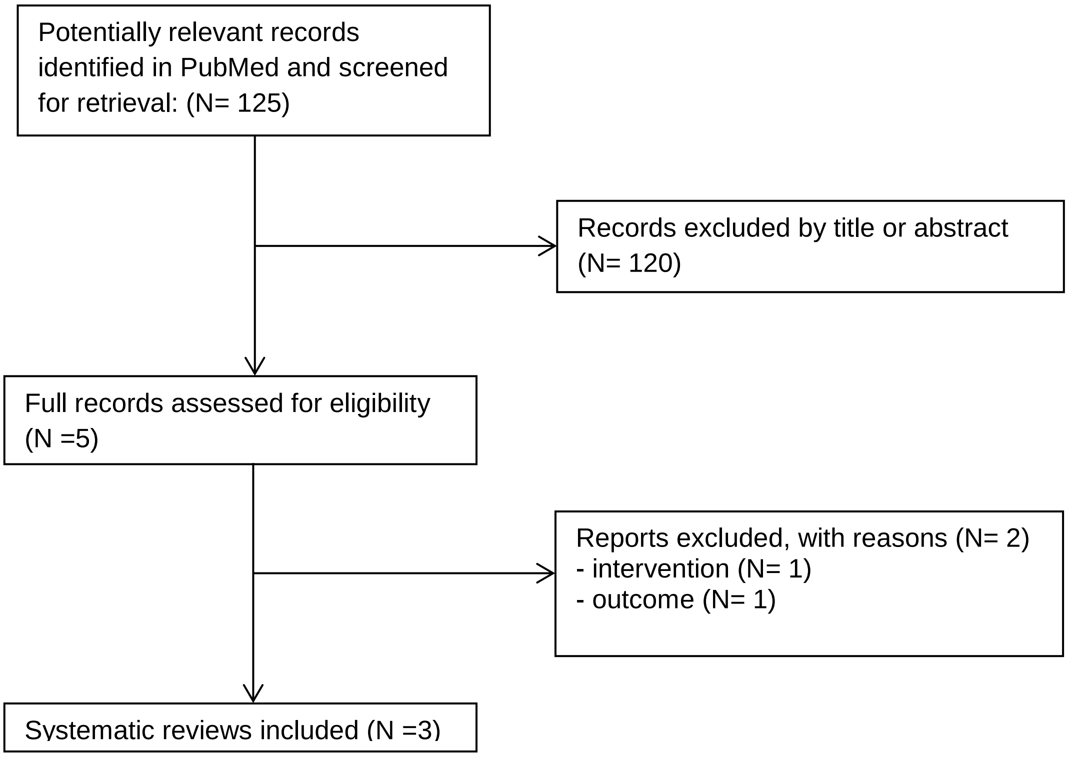

Gómez -Outes[171] PubMed search term: (((("dabigatran"[Supplementary Concept] OR "dabigatran"[All Fields]) OR ("rivaroxaban"[Supplementary Concept] OR "rivaroxaban"[All Fields])) OR ("apixaban"[Supplementary Concept] OR "apixaban"[All Fields])) AND ("arthroplasty, replacement, hip"[MeSH Terms] OR ("arthroplasty"[All Fields] AND "replacement"[All Fields] AND "hip"[All Fields]) OR "hip replacement arthroplasty"[All Fields] OR ("hip"[All Fields] AND "replacement"[All Fields]) OR "hip replacement"[All Fields])) OR ("arthroplasty, replacement, knee"[MeSH Terms] OR ("arthroplasty"[All Fields] AND "replacement"[All Fields] AND "knee"[All Fields]) OR "knee replacement arthroplasty"[All Fields] OR ("knee"[All Fields] AND "replacement"[All Fields]) OR "knee replacement"[All Fields]) AND (venous thromboemboli[All Fields] OR venous thromboembolic[All Fields] OR venous thromboembolism[All Fields] OR venous thromboembolisms[All Fields] OR venous thromboembolus[All Fields]) AND ((systematic[sb] OR Meta-Analysis[ptyp]) AND ("2002/06/14"[PDAT] : "2011/06/14"[PDAT]))

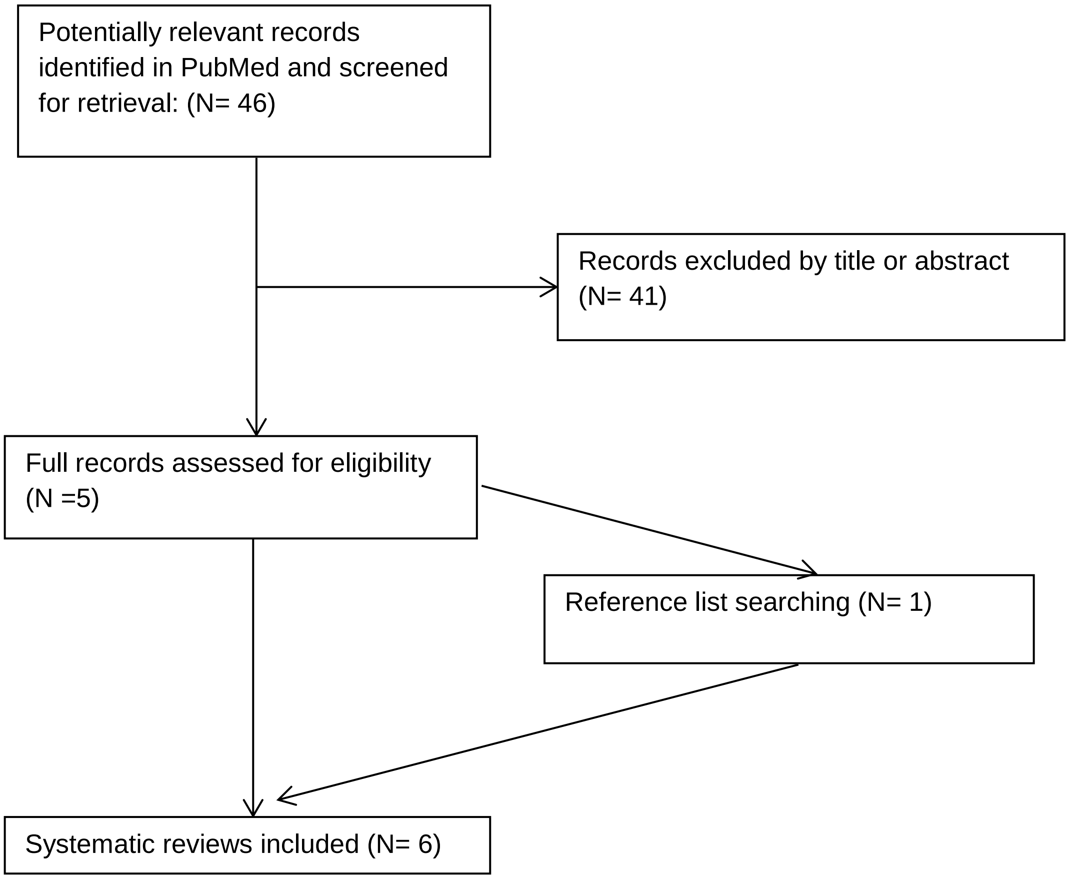

Jardine[177] PubMed search term: ("folic acid"[MeSH Terms] OR ("folic"[All Fields] AND "acid"[All Fields]) OR "folic acid"[All Fields]) AND ("homocysteine"[MeSH Terms] OR "homocysteine"[All Fields]) AND ((systematic[sb] OR Meta-Analysis[ptyp]) AND ("2002/06/13"[PDAT] : "2011/06/13"[PDAT]))

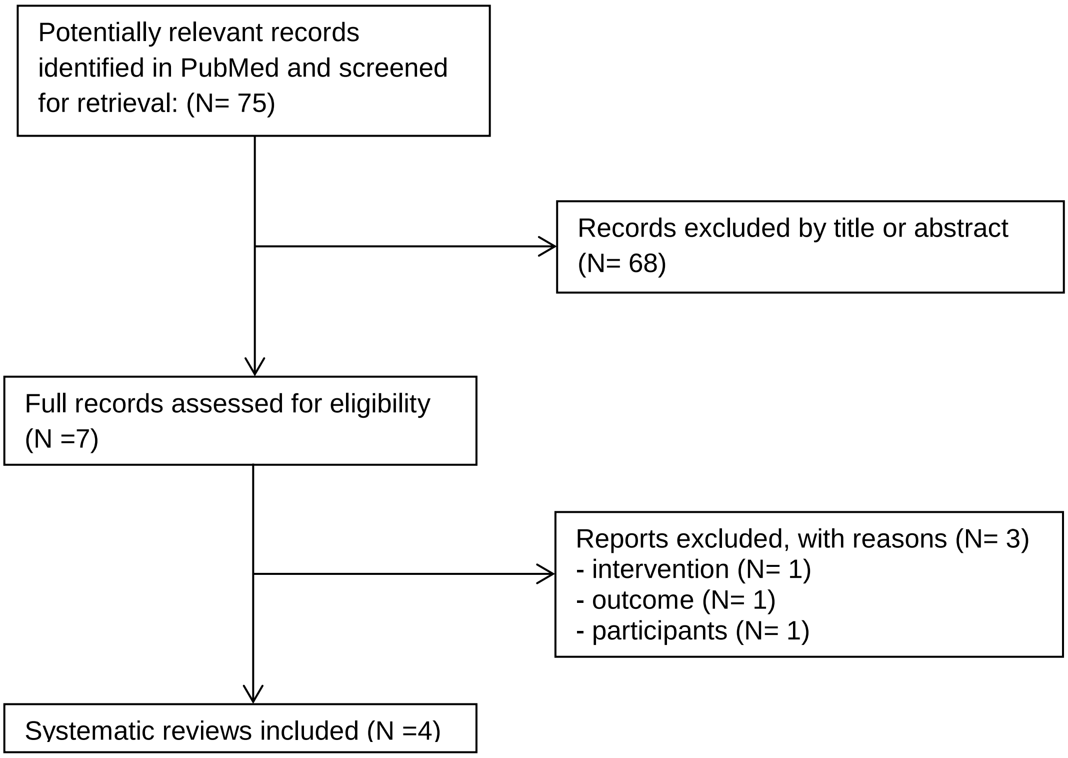

Leucht[182] PubMed search term: (("antipsychotic agents"[Pharmacological Action] OR "antipsychotic agents"[MeSH Terms] OR ("antipsychotic"[All Fields] AND "agents"[All Fields]) OR "antipsychotic agents"[All Fields] OR "antipsychotic"[All Fields]) AND (("recurrence"[MeSH Terms] OR "recurrence"[All Fields] OR "relapse"[All Fields]) AND ("prevention and control"[Subheading] OR ("prevention"[All Fields] AND "control"[All Fields]) OR "prevention and control"[All Fields] OR "prevention"[All Fields]))) AND ("schizophrenia"[MeSH Terms] OR "schizophrenia"[All Fields]) AND ((systematic[sb] OR Meta-Analysis[ptyp]) AND ("2002/06/02"[PDAT] : "2011/06/02"[PDAT]))

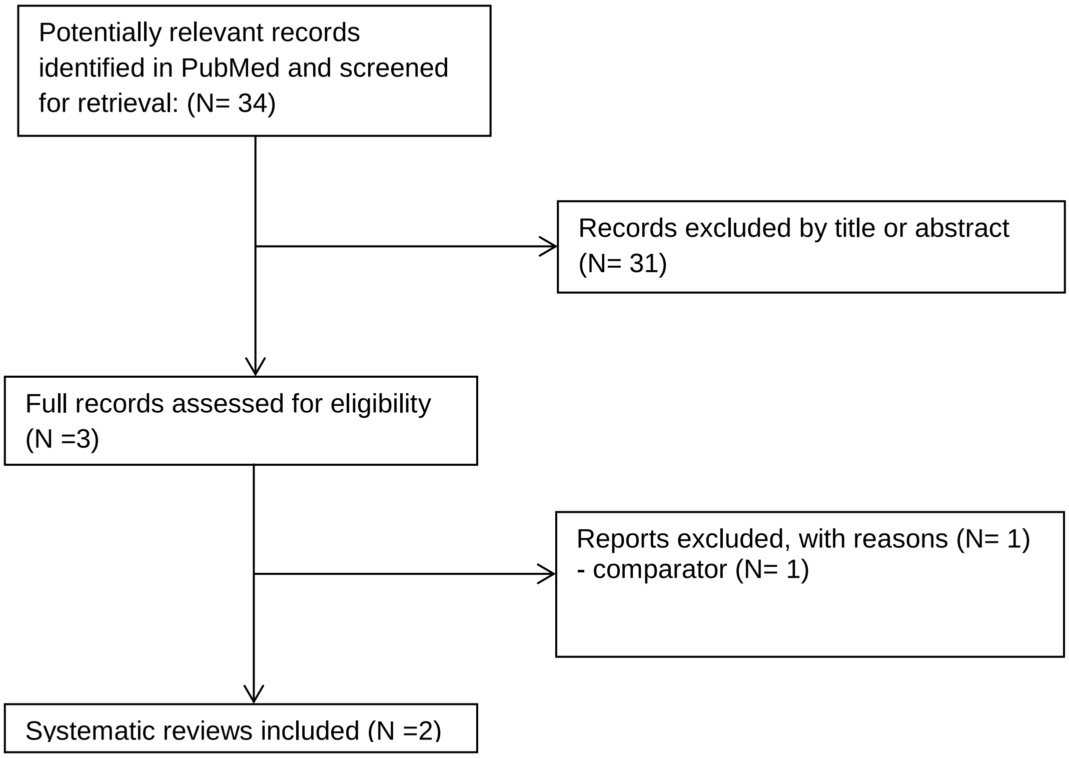

Ker[184] PubMed search term: ("tranexamic acid"[MeSH Terms] OR ("tranexamic"[All Fields] AND "acid"[All Fields]) OR "tranexamic acid"[All Fields]) AND (("surgical procedures, operative"[MeSH Terms] OR ("surgical"[All Fields] AND "procedures"[All Fields] AND "operative"[All Fields]) OR "operative surgical procedures"[All Fields] OR "surgical"[All Fields]) AND ("hemorrhage"[MeSH Terms] OR "hemorrhage"[All Fields] OR "bleeding"[All Fields])) AND ((systematic[sb] OR Meta-Analysis[ptyp]) AND ("2002/05/21"[PDAT] : "2011/05/21"[PDAT]))

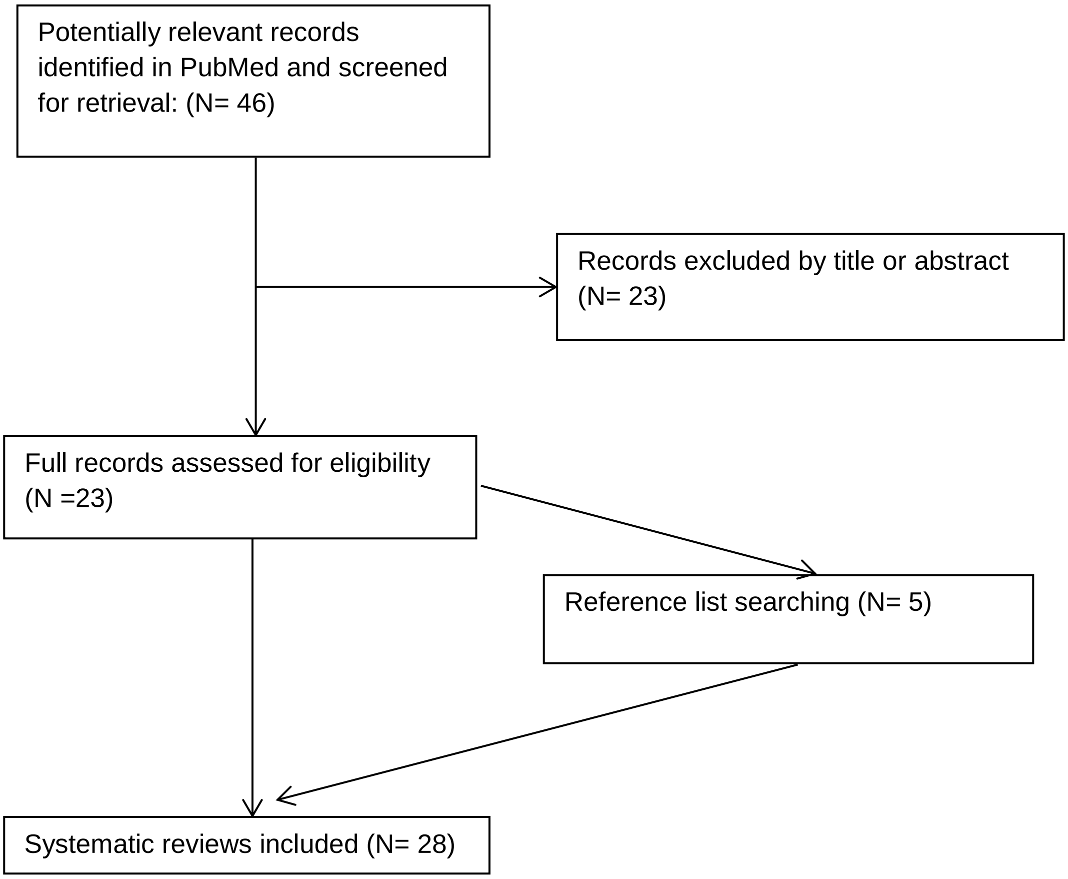

Neumann[209] PubMed search term: (((("dabigatran"[Supplementary Concept] OR "dabigatran"[All Fields]) OR ("rivaroxaban"[Supplementary Concept] OR "rivaroxaban"[All Fields])) OR ("apixaban"[Supplementary Concept] OR "apixaban"[All Fields])) AND ("arthroplasty, replacement, hip"[MeSH Terms] OR ("arthroplasty"[All Fields] AND "replacement"[All Fields] AND "hip"[All Fields]) OR "hip replacement arthroplasty"[All Fields] OR ("hip"[All Fields] AND "replacement"[All Fields]) OR "hip replacement"[All Fields])) OR ("arthroplasty, replacement, knee"[MeSH Terms] OR ("arthroplasty"[All Fields] AND "replacement"[All Fields] AND "knee"[All Fields]) OR "knee replacement arthroplasty"[All Fields] OR ("knee"[All Fields] AND "replacement"[All Fields]) OR "knee replacement"[All Fields]) AND (venous thromboemboli[All Fields] OR venous thromboembolic[All Fields] OR ("venous thromboembolism"[MeSH Terms] OR ("venous"[All Fields] AND "thromboembolism"[All Fields]) OR "venous thromboembolism"[All Fields]) OR venous thromboembolisms[All Fields] OR venous thromboembolus[All Fields]) AND ((systematic[sb] OR Meta-Analysis[ptyp]) AND ("2002/03/13"[PDAT] : "2011/03/13"[PDAT]))

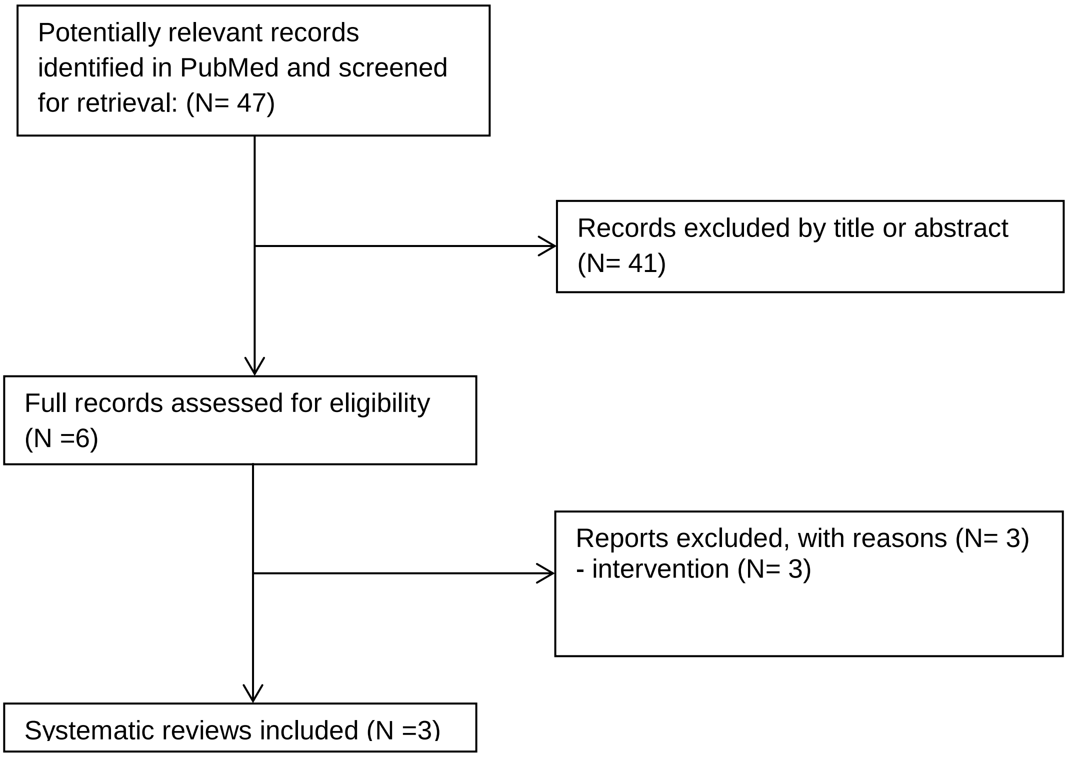

Boekholdt[210] PubMed search term: ("hydroxymethylglutaryl-coa reductase inhibitors"[Pharmacological Action] OR "hydroxymethylglutaryl-coa reductase inhibitors"[MeSH Terms] OR ("hydroxymethylglutaryl-coa"[All Fields] AND "reductase"[All Fields] AND "inhibitors"[All Fields]) OR "hydroxymethylglutaryl-coa reductase inhibitors"[All Fields] OR "statins"[All Fields]) AND (("cardiovascular system"[MeSH Terms] OR ("cardiovascular"[All Fields] AND "system"[All Fields]) OR "cardiovascular system"[All Fields] OR "cardiovascular"[All Fields]) AND events[All Fields]) AND ((Meta-Analysis[ptyp] OR systematic[sb]) AND ("2002/03/29"[PDAT] : "2011/03/29"[PDAT]))

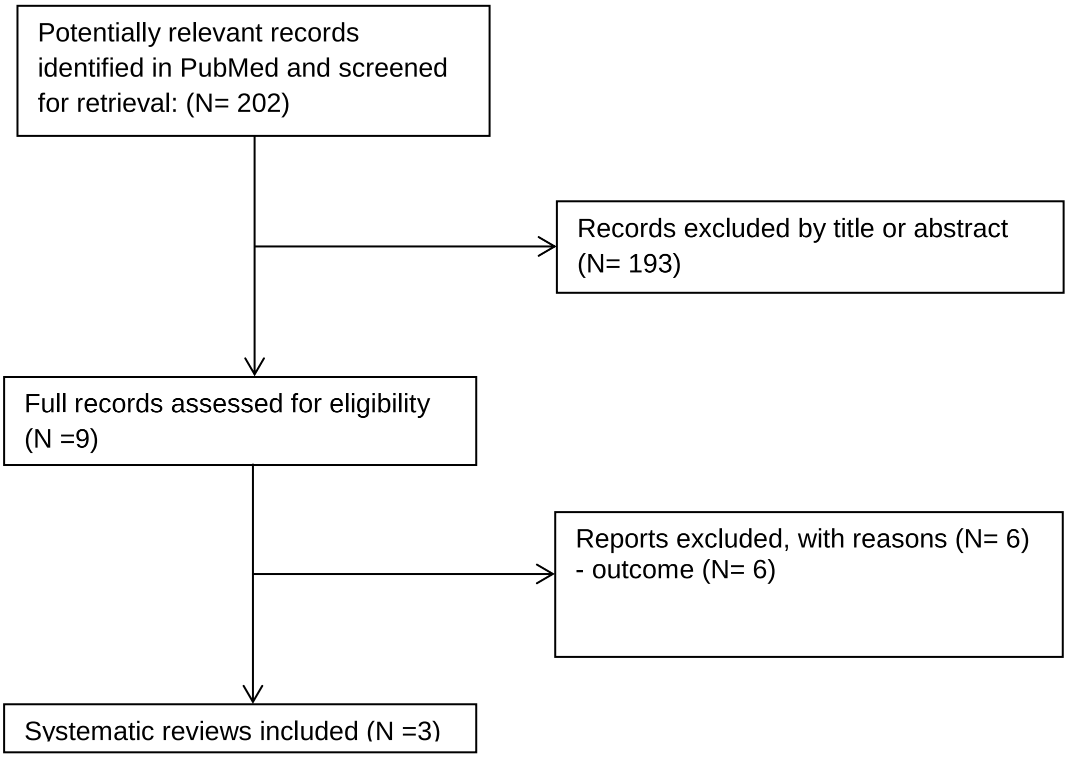

Hempel[31] PubMed search term:("probiotics"[MeSH Terms] OR "probiotics"[All Fields]) AND (("anti-bacterial agents"[Pharmacological Action] OR "anti-bacterial agents"[MeSH Terms] OR ("anti-bacterial"[All Fields] AND "agents"[All Fields]) OR "anti-bacterial agents"[All Fields] OR "antibiotic"[All Fields]) AND associated[All Fields] AND ("diarrhoea"[All Fields] OR "diarrhea"[MeSH Terms] OR "diarrhea"[All Fields])) AND ((Meta-Analysis[ptyp] OR systematic[sb]) AND ("2002/05/09"[PDAT] : "2011/05/09"[PDAT]))

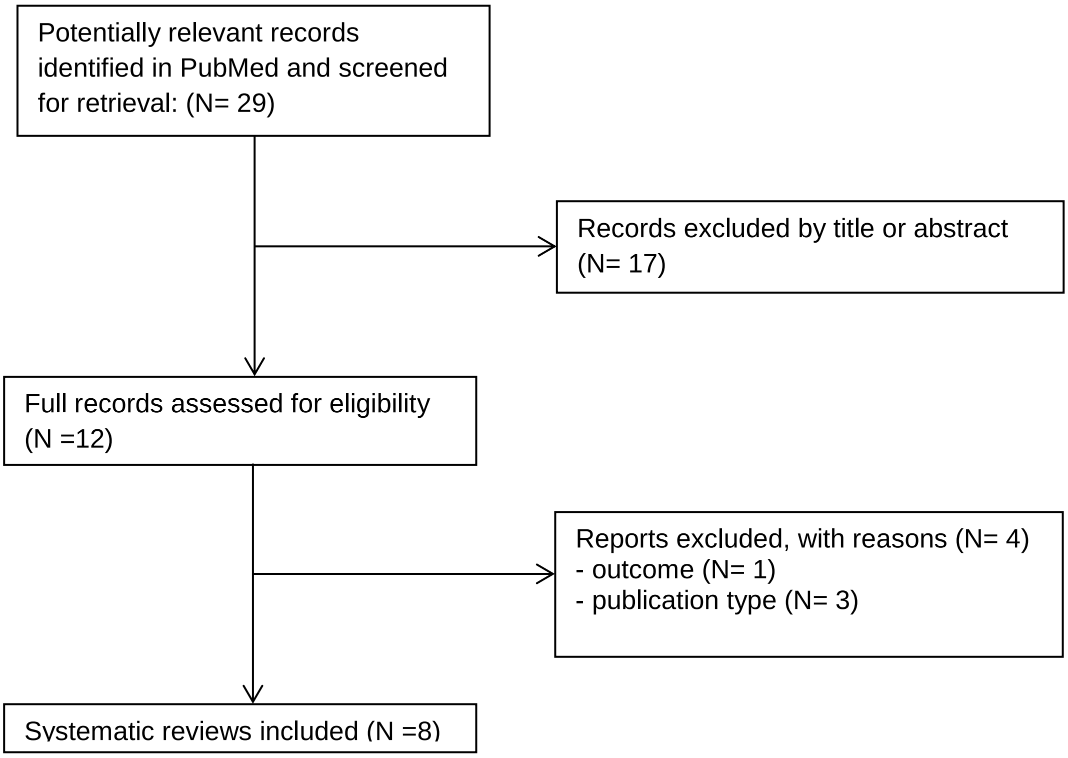

Jackson[212] PubMed search term: (("migraine disorders"[MeSH Terms] OR ("migraine"[All Fields] AND "disorders"[All Fields]) OR "migraine disorders"[All Fields] OR "migraine"[All Fields]) OR ("tension-type headache"[MeSH Terms] OR ("tension-type"[All Fields] AND "headache"[All Fields]) OR "tension-type headache"[All Fields] OR ("tension"[All Fields] AND "headaches"[All Fields]) OR "tension headaches"[All Fields])) AND ("botulinum toxins"[MeSH Terms] OR ("botulinum"[All Fields] AND "toxins"[All Fields]) OR "botulinum toxins"[All Fields] OR ("botulinum"[All Fields] AND "toxin"[All Fields]) OR "botulinum toxin"[All Fields]) AND ((Meta-Analysis[ptyp] OR systematic[sb]) AND ("2002/04/25"[PDAT] : "2011/04/25"[PDAT]))

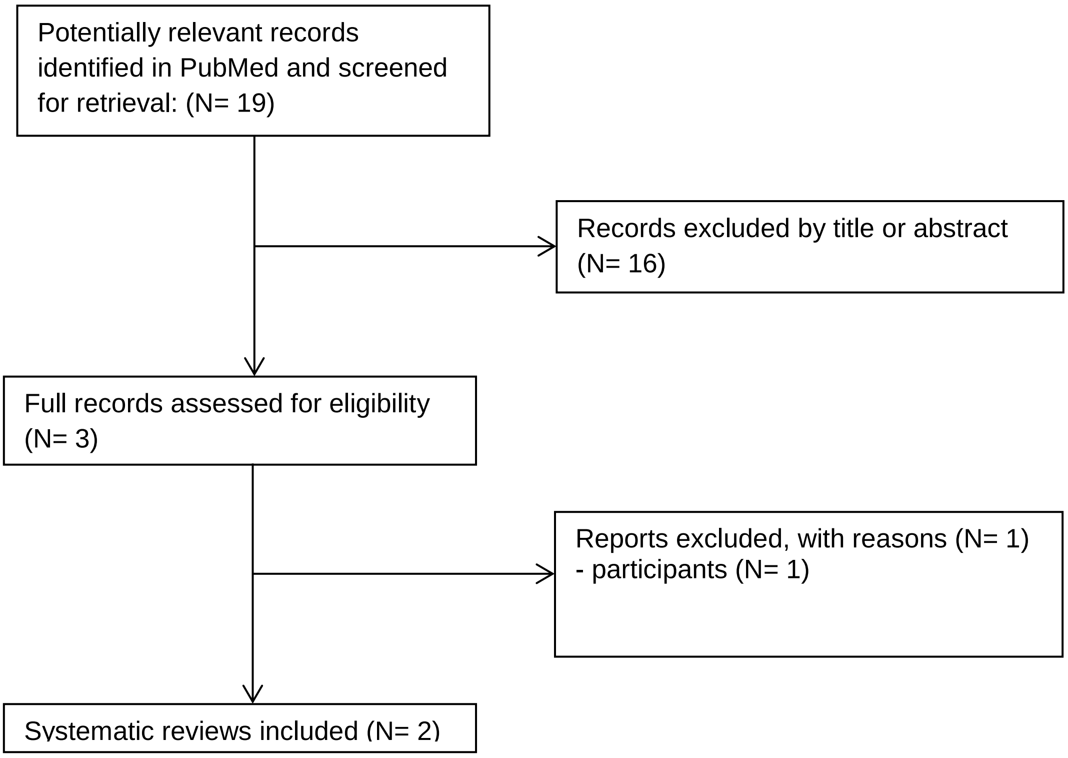

Hemmingsen[215] PubMed search term: (("metformin"[MeSH Terms] OR "metformin"[All Fields]) AND ("insulin"[MeSH Terms] OR "insulin"[All Fields])) AND ("diabetes mellitus, type 2"[MeSH Terms] OR "type 2 diabetes mellitus"[All Fields] OR "type 2 diabetes"[All Fields]) AND ((Meta-Analysis[ptyp] OR systematic[sb]) AND ("2002/04/19"[PDAT] : "2011/04/19"[PDAT]))

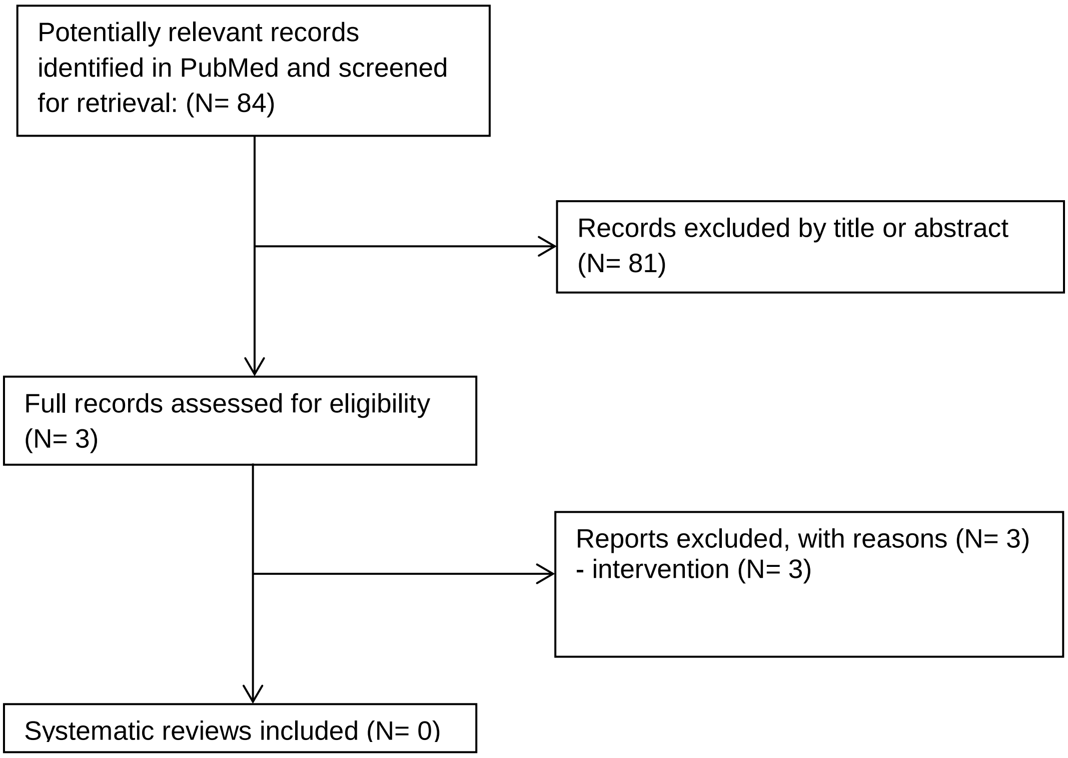

Palmerini[216] PubMed search term: (("thrombosis"[MeSH Terms] OR "thrombosis"[All Fields]) AND drug-eluting[All Fields]) AND bare-metal[All Fields] AND ((Meta-Analysis[ptyp] OR systematic[sb]) AND ("2002/04/14"[PDAT] : "2011/04/14"[PDAT]))

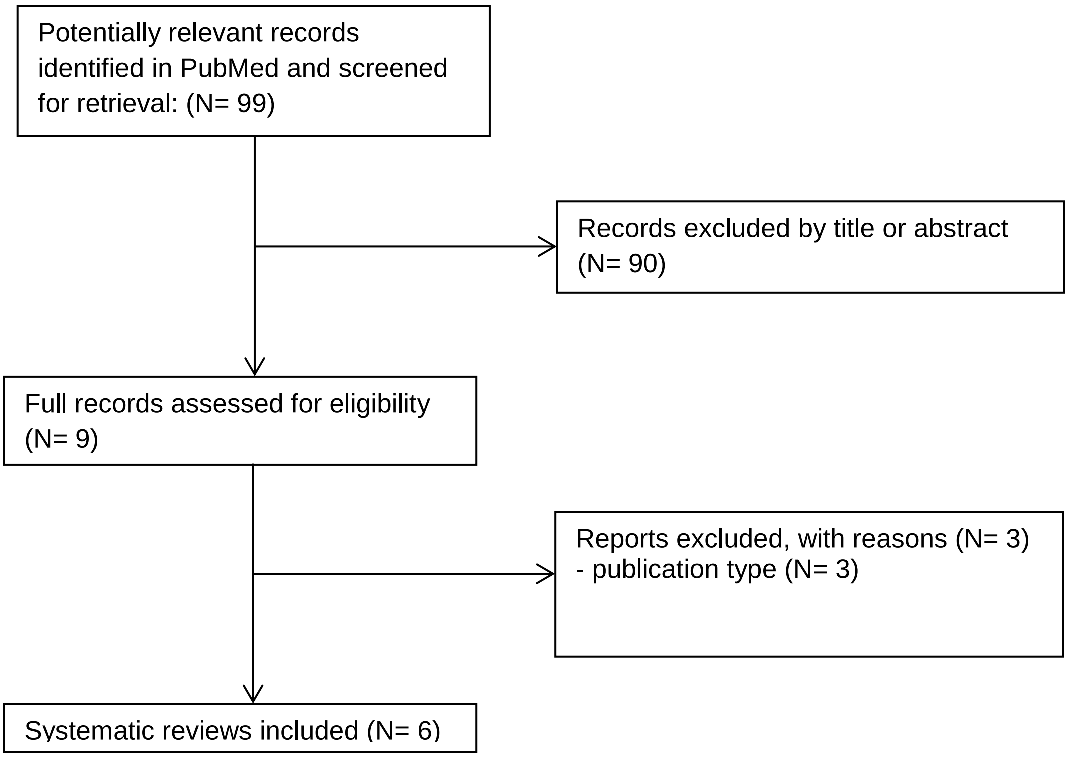

Varadhan[221] PubMed search term: (("appendicitis"[MeSH Terms] OR "appendicitis"[All Fields]) OR ("anti-bacterial agents"[Pharmacological Action] OR "anti-bacterial agents"[MeSH Terms] OR ("anti-bacterial"[All Fields] AND "agents"[All Fields]) OR "anti-bacterial agents"[All Fields] OR "antibiotics"[All Fields])) AND ("appendectomy"[MeSH Terms] OR "appendectomy"[All Fields] OR "appendicectomy"[All Fields]) AND ((Meta-Analysis[ptyp] OR systematic[sb]) AND ("2002/04/05"[PDAT] : "2011/04/05"[PDAT]))

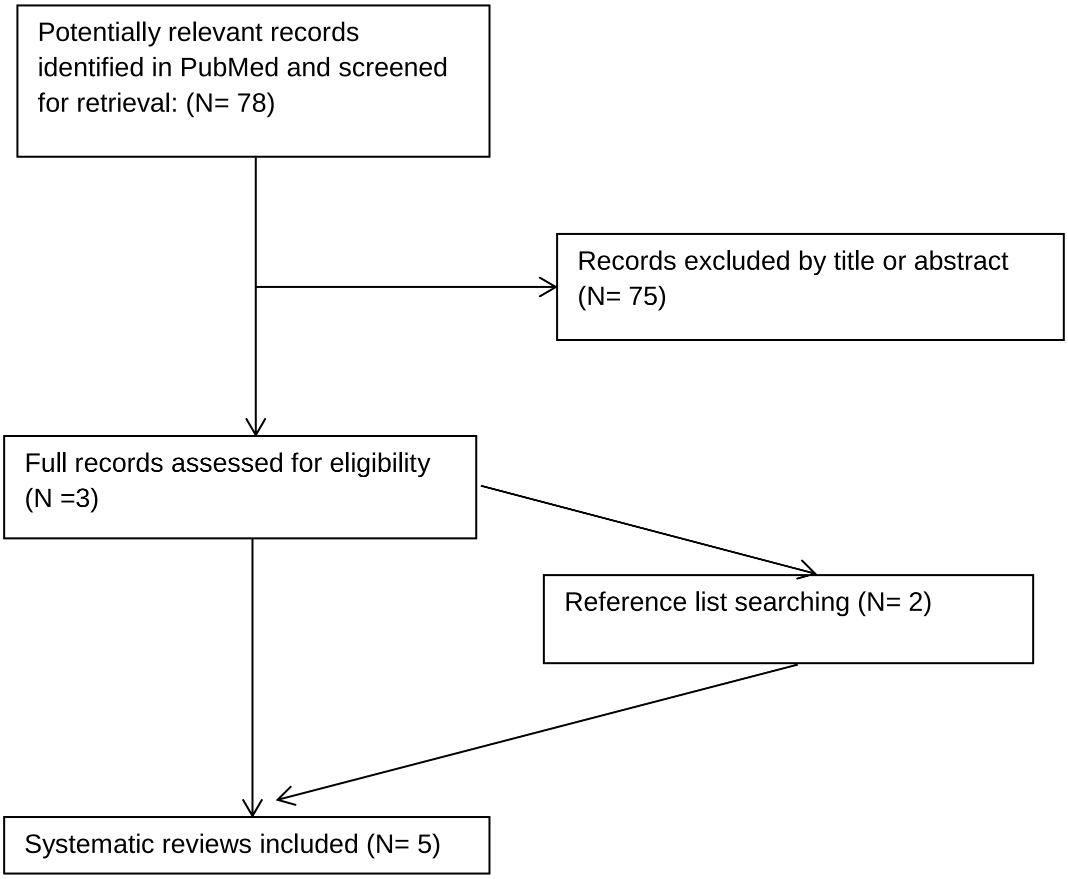

Hsu[227] PubMed search term: (((("oseltamivir"[MeSH Terms] OR "oseltamivir"[All Fields]) OR ("zanamivir"[MeSH Terms] OR "zanamivir"[All Fields])) OR ("amantadine"[MeSH Terms] OR "amantadine"[All Fields])) OR ("rimantadine"[MeSH Terms] OR "rimantadine"[All Fields])) AND ("influenza, human"[MeSH Terms] OR ("influenza"[All Fields] AND "human"[All Fields]) OR "human influenza"[All Fields] OR "influenza"[All Fields]) AND ((Meta-Analysis[ptyp] OR systematic[sb]) AND ("2002/02/28"[PDAT] : "2011/02/28"[PDAT]))

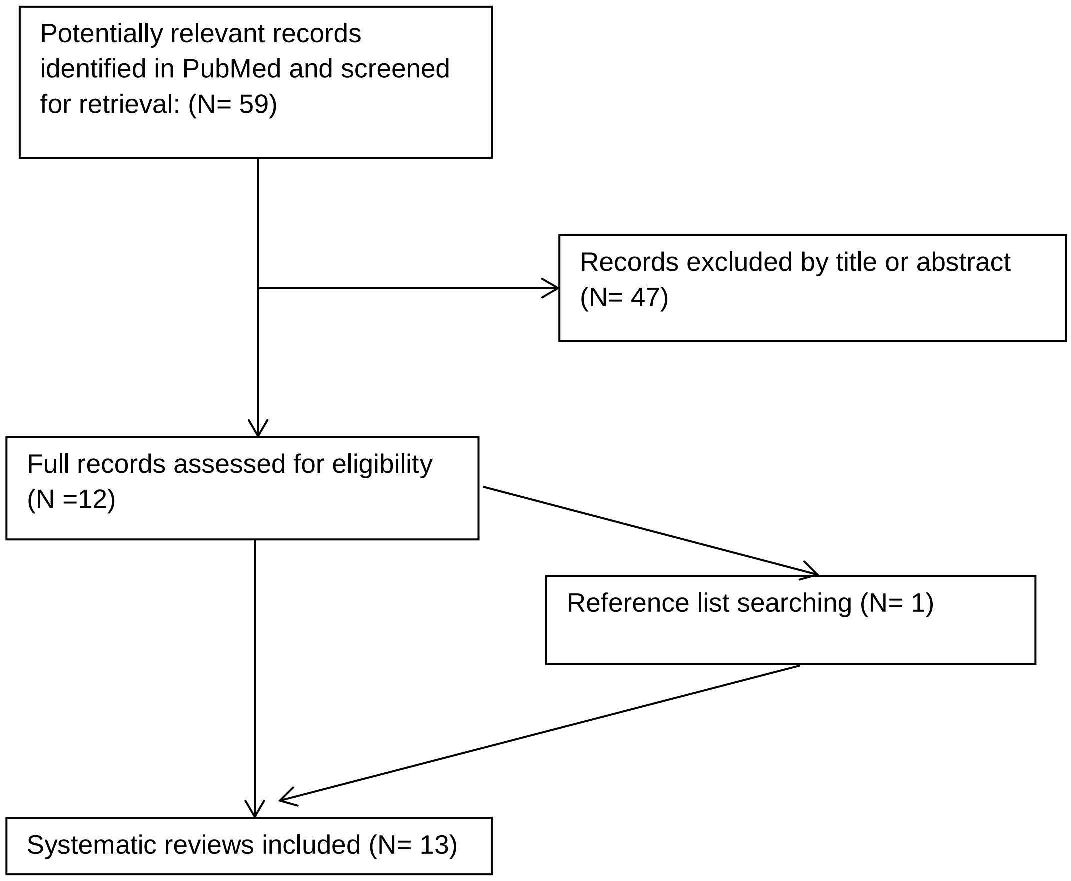

Boussageon[241] PubMed search term: ("metformin"[MeSH Terms] OR "metformin"[All Fields]) AND ("diabetes mellitus, type 2"[MeSH Terms] OR "type 2 diabetes mellitus"[All Fields] OR "type 2 diabetes"[All Fields]) AND ((Meta-Analysis[ptyp] OR systematic[sb]) AND ("2002/04/10"[PDAT] : "2011/04/10"[PDAT]))

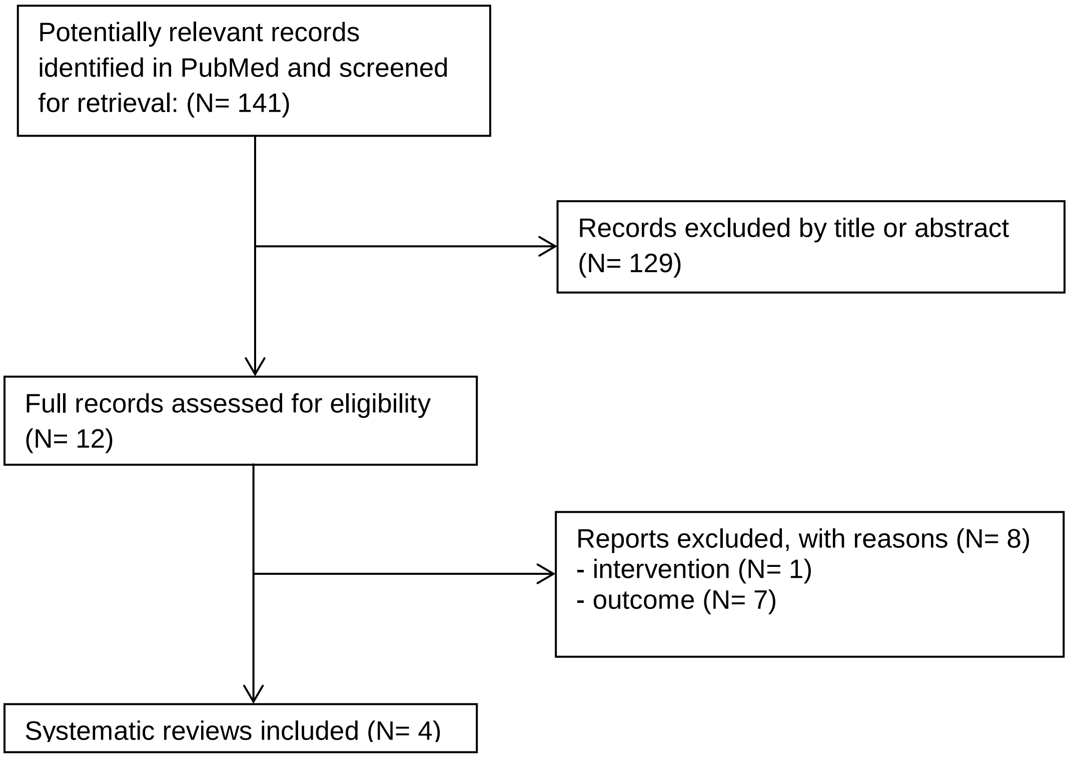

Palmer[246] PubMed search term: (antiplatelet[All Fields] AND ("therapy"[Subheading] OR "therapy"[All Fields] OR "therapeutics"[MeSH Terms] OR "therapeutics"[All Fields])) AND (chronic[All Fields] AND ("kidney diseases"[MeSH Terms] OR ("kidney"[All Fields] AND "diseases"[All Fields]) OR "kidney diseases"[All Fields] OR ("kidney"[All Fields] AND "disease"[All Fields]) OR "kidney disease"[All Fields])) AND ((Meta-Analysis[ptyp] OR systematic[sb]) AND ("2002/03/20"[PDAT] : "2011/03/20"[PDAT]))

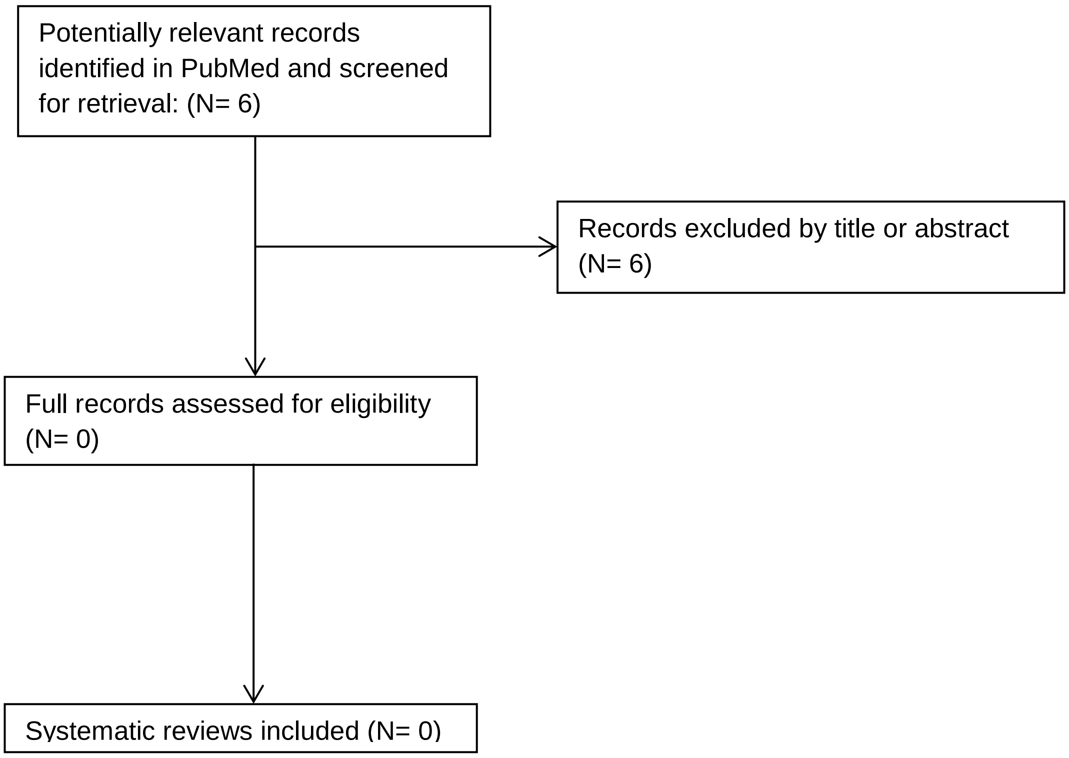

Harel[6] PubMed search term: (("aliskiren"[Supplementary Concept] OR "aliskiren"[All Fields]) OR ("renin-angiotensin system"[MeSH Terms] OR ("renin-angiotensin"[All Fields] AND "system"[All Fields]) OR "renin-angiotensin system"[All Fields] OR ("renin"[All Fields] AND "angiotensin"[All Fields] AND "system"[All Fields]) OR "renin angiotensin system"[All Fields])) AND (("acute kidney injury"[MeSH Terms] OR ("acute"[All Fields] AND "kidney"[All Fields] AND "injury"[All Fields]) OR "acute kidney injury"[All Fields]) OR ("hyperkalaemia"[All Fields] OR "hyperkalemia"[MeSH Terms] OR "hyperkalemia"[All Fields])) AND ((Meta-Analysis[ptyp] OR systematic[sb]) AND ("2002/01/09"[PDAT] : "2011/01/09"[PDAT]))

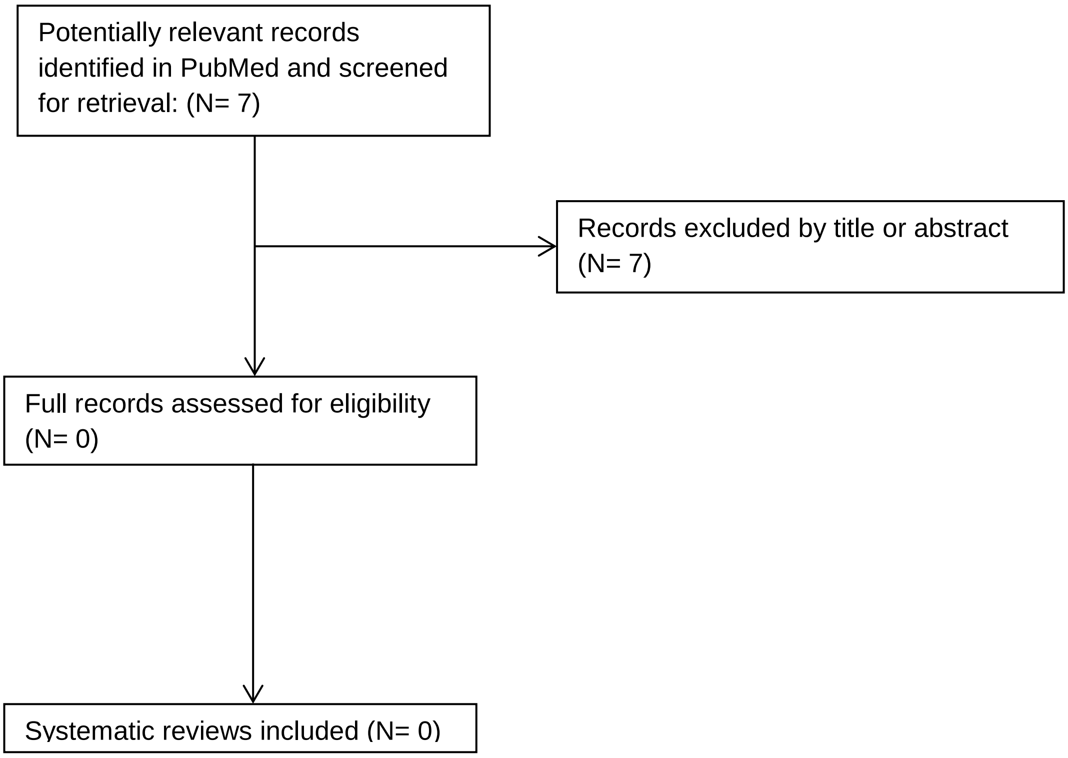

McKnight[247] PubMed search term: (("bipolar disorder"[MeSH Terms] OR ("bipolar"[All Fields] AND "disorder"[All Fields]) OR "bipolar disorder"[All Fields]) OR ("mood disorders"[MeSH Terms] OR ("mood"[All Fields] AND "disorders"[All Fields]) OR "mood disorders"[All Fields] OR ("mood"[All Fields] AND "disorder"[All Fields]) OR "mood disorder"[All Fields])) AND ("lithium"[MeSH Terms] OR "lithium"[All Fields]) AND ((Meta-Analysis[ptyp] OR systematic[sb]) AND ("2002/02/25"[PDAT] : "2011/02/25"[PDAT]))

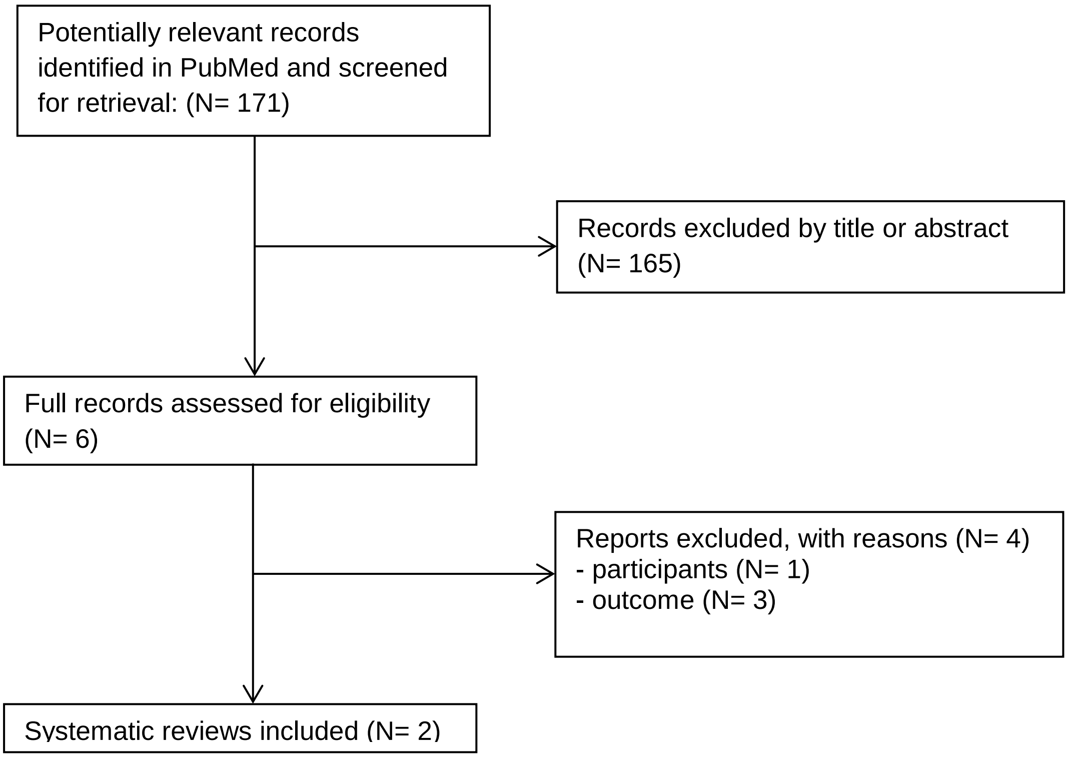

Pinto[250] PubMed search term: ((((((("anti-inflammatory agents, non-steroidal"[Pharmacological Action] OR "anti-inflammatory agents, non-steroidal"[MeSH Terms] OR ("anti-inflammatory"[All Fields] AND "agents"[All Fields] AND "non-steroidal"[All Fields]) OR "non-steroidal anti-inflammatory agents"[All Fields] OR "nsaids"[All Fields]) OR ("adrenal cortex hormones"[Pharmacological Action] OR "adrenal cortex hormones"[MeSH Terms] OR ("adrenal"[All Fields] AND "cortex"[All Fields] AND "hormones"[All Fields]) OR "adrenal cortex hormones"[All Fields] OR "corticosteroids"[All Fields])) OR ("antidepressive agents"[Pharmacological Action] OR "antidepressive agents"[MeSH Terms] OR ("antidepressive"[All Fields] AND "agents"[All Fields]) OR "antidepressive agents"[All Fields] OR "antidepressants"[All Fields])) OR ("anticonvulsants"[Pharmacological Action] OR "anticonvulsants"[MeSH Terms] OR "anticonvulsants"[All Fields])) OR (("muscles"[MeSH Terms] OR "muscles"[All Fields] OR "muscle"[All Fields]) AND relaxants[All Fields])) OR ("analgesics, opioid"[Pharmacological Action] OR "analgesics, opioid"[MeSH Terms] OR ("analgesics"[All Fields] AND "opioid"[All Fields]) OR "opioid analgesics"[All Fields] OR "opioids"[All Fields])) AND ("sciatica"[MeSH Terms] OR "sciatica"[All Fields]) AND ((Meta-Analysis[ptyp] OR systematic[sb]) AND ("2002/02/13"[PDAT] : "2011/02/13"[PDAT]))

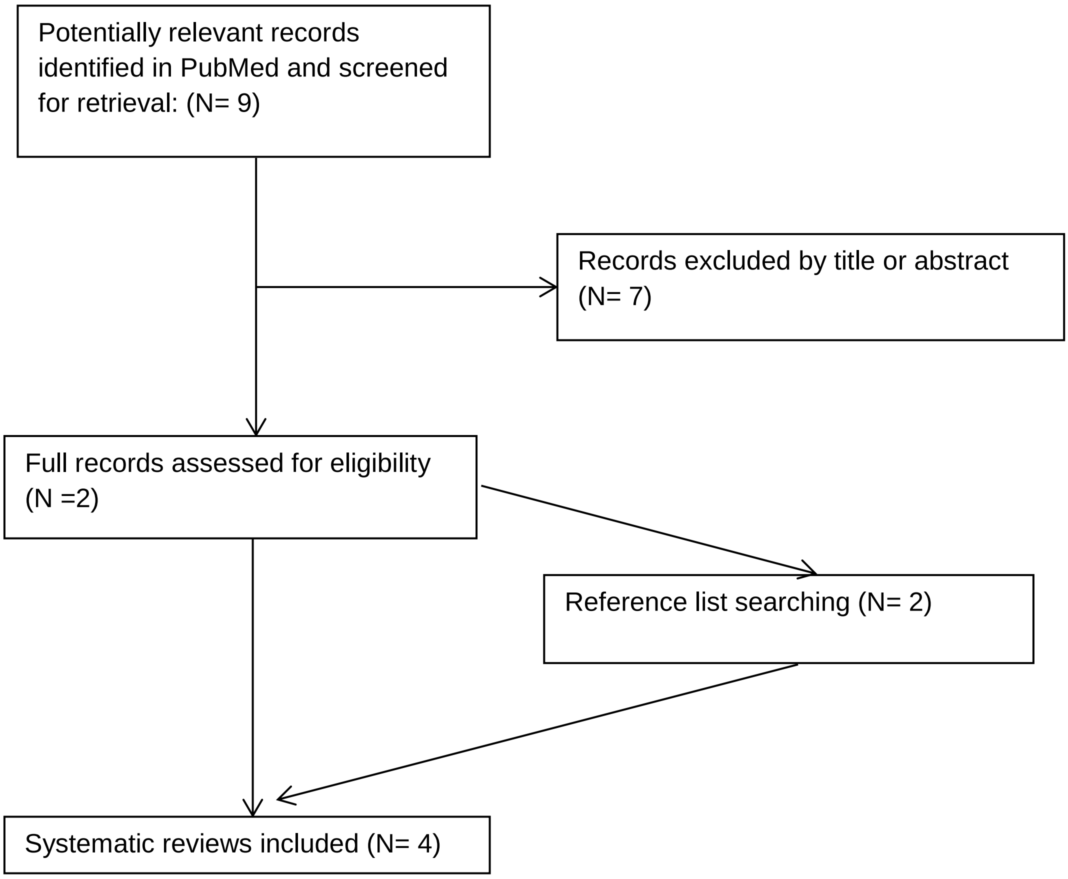

EBCTCG[254] PubMed search term: ("drug therapy, combination"[MeSH Terms] OR ("drug"[All Fields] AND "therapy"[All Fields] AND "combination"[All Fields]) OR "combination drug therapy"[All Fields] OR "polychemotherapy"[All Fields]) AND (early[All Fields] AND ("breast neoplasms"[MeSH Terms] OR ("breast"[All Fields] AND "neoplasms"[All Fields]) OR "breast neoplasms"[All Fields] OR ("breast"[All Fields] AND "cancer"[All Fields]) OR "breast cancer"[All Fields])) AND ((Meta-Analysis[ptyp] OR systematic[sb]) AND ("2002/02/04"[PDAT] : "2011/02/04"[PDAT]))

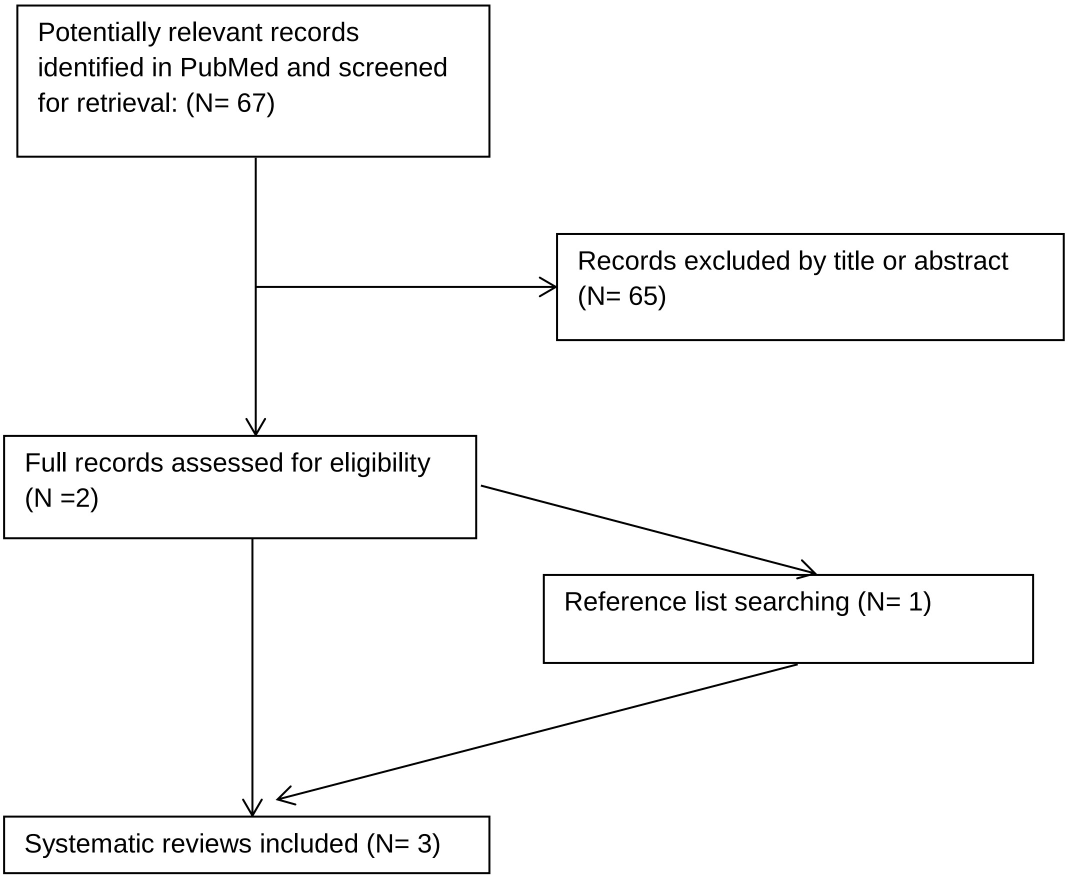

Silvain[258] PubMed search term: ("percutaneous coronary intervention"[MeSH Terms] OR ("percutaneous"[All Fields] AND "coronary"[All Fields] AND "intervention"[All Fields]) OR "percutaneous coronary intervention"[All Fields]) AND (("enoxaparin"[MeSH Terms] OR "enoxaparin"[All Fields]) OR ("heparin"[MeSH Terms] OR "heparin"[All Fields])) AND ((Meta-Analysis[ptyp] OR systematic[sb]) AND ("2002/02/03"[PDAT] : "2011/02/03"[PDAT]))

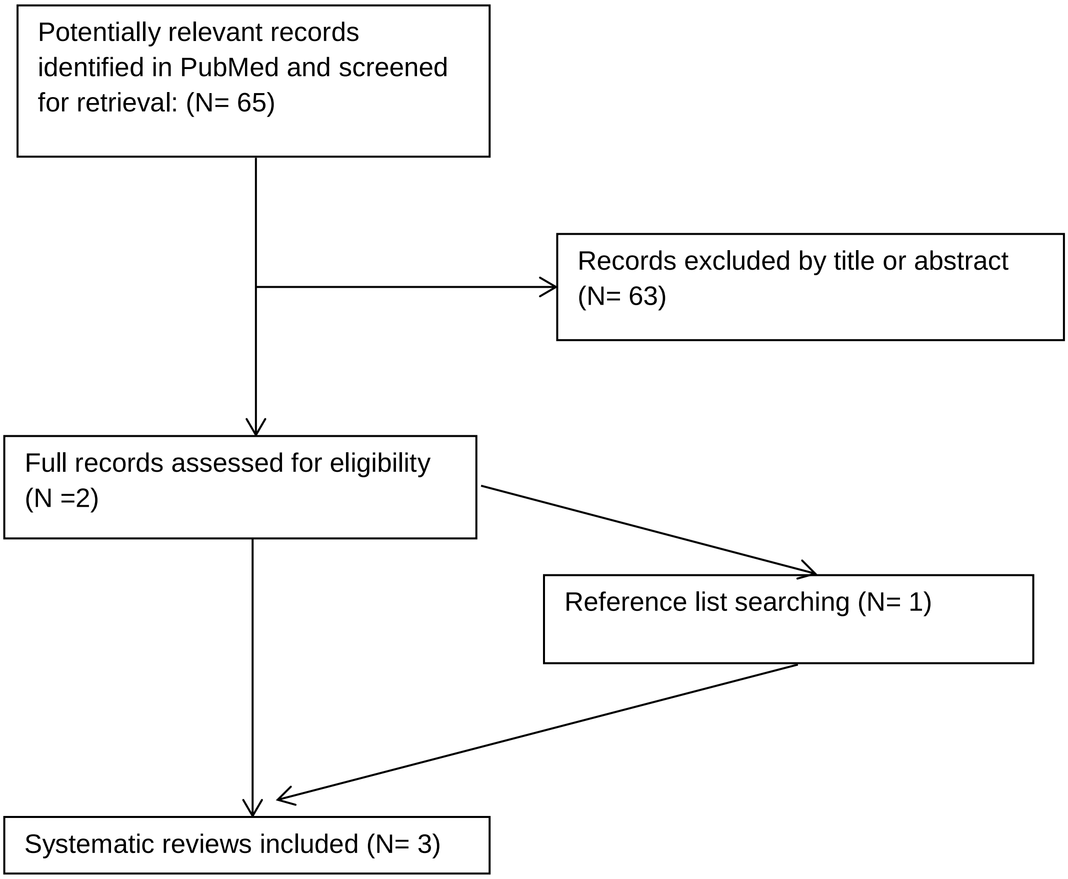

Vilsboll[262] PubMed search term: ((("glucagon-like peptide-1 receptor"[Supplementary Concept] OR "glucagon-like peptide-1 receptor"[All Fields] OR "glucagon like peptide 1 receptor"[All Fields]) AND ("agonists"[Subheading] OR "agonists"[All Fields])) OR ("exenatide"[Supplementary Concept] OR "exenatide"[All Fields])) OR ("liraglutide"[Supplementary Concept] OR "liraglutide"[All Fields]) AND ((Meta-Analysis[ptyp] OR systematic[sb]) AND ("2002/01/10"[PDAT] : "2011/01/10"[PDAT]))

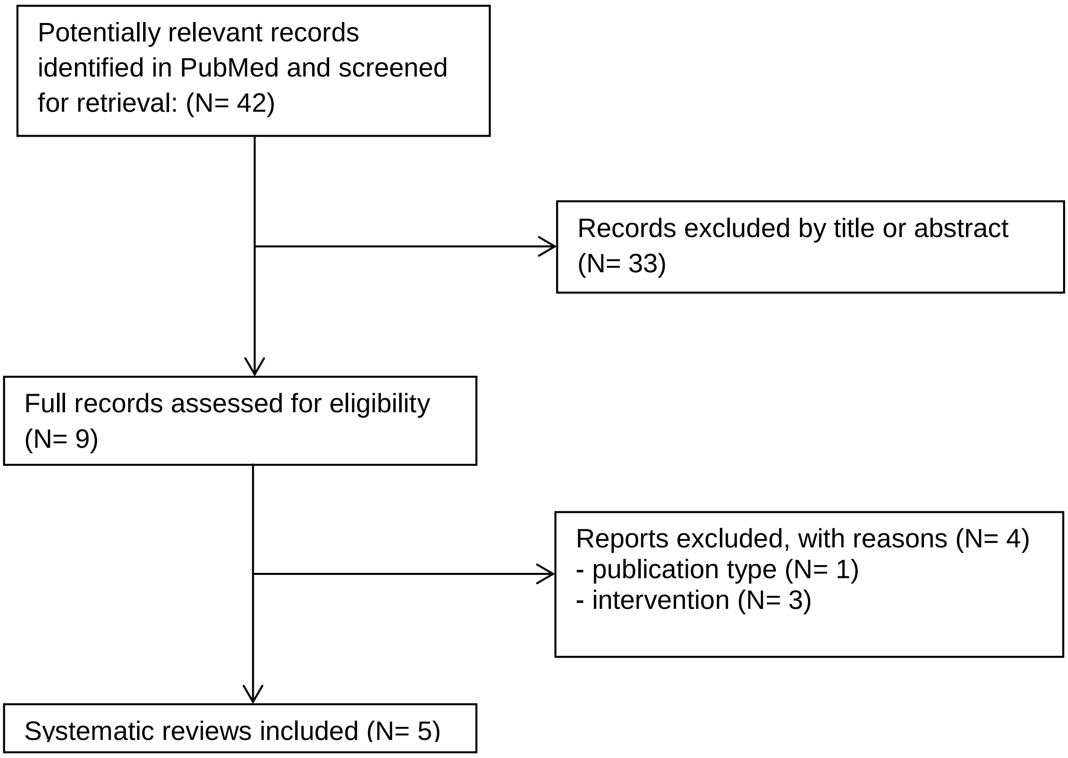

Supplement: Additional file 2: — PRISMA flow diagrams for previous systematic reviews and meta-analyses. [file 12916_2015_317_MOESM2_ESM.pdf]
