# Supplementary material for: Recent meta-analyses neglect previous systematic reviews and meta-analyses about the same topic: a systematic examination
Source: BMC Med. 2015 Apr 14;13:82. doi: 10.1186/s12916-015-0317-4 (PMC4411715; doi:10.1186/s12916-015-0317-4)
Supplement: Additional file 3: — Similarity score of the review question between recent and previous review: definitions and examples. [file 12916_2015_317_MOESM3_ESM.pdf]

Additional file 3

Similarity score of the review question between recent and previous review: definitions and examples

The similarity score was based on a comparison of PICO questions reconstructed for each recent and previous review. PICO questions are used to formulate a clinical question and provide information about participants (P), intervention (I), comparator (C) and outcomes (O) of a given study.

Exclusion criterion:

— Completely different (non-overlapping) PICO questions:

If at least one completely different (non-overlaping) PICO question between recent and previous study was identified, the previous study was excluded.

For example: only outcome-A was presented for the previous and only outcome-B for the recent study.

Scoring:

— Overlapping PICO questions:

When a specific PICO question between a previous and recent article was overlapping, zero points were given.

For example: outcome-A and outcome-B in the previous and only outcome-A in the recent study

— Identical PICO questions:

If a specific PICO question between a previous and recent article was identical, one point was given.

For example: outcome-A in the previous and outcome-A in the recent study.

Specific example:

The recent meta-analysis comparing enoxaparin versus unfractionated heparin during percutaneous coronary intervention by Silvain[258] used mortality and major bleeding as outcomes. A previous meta-analysis by Navarese[261] used exactly the same outcomes so 1 point was given, whereas another previous meta-analysis by Borentain[259] used different but overlapping outcomes (ischemic events: usually a composite of death, myocardial infarction, and urgent revascularization; bleeding: major, minor, or all bleeding), so 0 points were given. Other previous meta-analyses that used different outcomes that were not overlapping and did not include mortality or major bleeding (but for example only "quality of life") would have been excluded.
