# Supplementary material for: Recent meta-analyses neglect previous systematic reviews and meta-analyses about the same topic: a systematic examination
Source: BMC Med. 2015 Apr 14;13:82. doi: 10.1186/s12916-015-0317-4 (PMC4411715; doi:10.1186/s12916-015-0317-4)
Supplement: Additional file 4: — Concordance of results: example of similar and different results. [file 12916_2015_317_MOESM4_ESM.pdf]

Additional file 4

Concordance of results: example of similar and different results

The recent meta-analysis by Jardine[177] on the effect of folic acid based homocysteine lowering on cardiovascular events in people with kidney disease reported that folic acid based homocysteine therapy did not prevent cardiovascular events (relative risk 0.97, 95% confidence interval 0.92 to 1.03, P=0.326).

- Example of similar results:

Similarly, the previous meta-analysis by Clarke[179] found that folic acid allocation had no significant effects on vascular outcomes, with rate ratios (95% confidence intervals) of 1.01 (0.97-1.05) for major vascular events, 1.03 (0.97-1.10) for major coronary events, and 0.96 (0.87-1.06) for stroke as well as no significant effects on vascular outcomes in any of the subgroups studied or on overall vascular mortality.

- Example of different results:

On the other hand, the previous meta-analysis by Qin found that when pooling the seven trials, folic acid therapy significantly reduced the risk of cardiovascular disease by 15% (relative risk, 0.85; 95% confidence interval, 0.76 to 0.96; P=0.009).
