# Supplementary material for: Recent meta-analyses neglect previous systematic reviews and meta-analyses about the same topic: a systematic examination
Source: BMC Med. 2015 Apr 14;13:82. doi: 10.1186/s12916-015-0317-4 (PMC4411715; doi:10.1186/s12916-015-0317-4)
Supplement: Additional file 5: — Summary characteristics of recent and previous studies. [file 12916_2015_317_MOESM5_ESM.pdf]

Additional file 5

Summary characteristics of recent and previous studies.

For columns “cited”, “described”, “discussed” 1 indicates that the previous study was cited/ described/ discussed by the recent meta-analysis and 0 that it was not. For “concordance of results” 0 indicates that the results were different and 1 that they were similar. For “PICO” 1 indicates “identical” and 0 indicates “different” items (see Additional file 3). For “methods” 1 indicates that meta-analytic methods were used and 2 indicates that they were not used. P = participants; I = intervention; C= comparator; O = outcomes

Recent study	Previous study	Publication year	Cited	Described	Discussed	Concordance of results	P	I	C	O	Similarity score	Methods	AMSTAR	Journal	Impact factor	Medical discipline
Chatterjee[12]		2013											8	BMJ	17,22	cardiology
	Abdulla[13]	2006	0	0	0	1	0	1	1	1	3	1		Eur J Heart Fail	5,25	
	Bouzamondo[14]	2003	0	0	0	1	1	1	1	1	4	1		Eur J Heart Fail	5,25	
	McAlister[15]	2009	1	0	0	1	1	1	1	0	3	1		Ann Intern Med	13,98	
Haase[16]		2013											9	BMJ	17,22	anaestesiology
	Bunn[17]	2012	1	1	0	0	0	0	0	1	1	1		Cochrane Database Syst Rev	5,7	& intensive therapy
	Dart[18]	2011	1	1	0	1	0	0	0	1	1	1		Cochrane Database Syst Rev	5,7	
	Gattas[19]	2012	1	1	0	0	0	0	0	1	1	1		Anesth Analg	3,3	
	Groeneveld[20]	2011	0	0	0	1	0	0	0	1	1	0		Ann Surg	6,33	
	Hartog[21]	2011	1	1	0	1	0	0	0	1	1	0		Anesth Analg	3,3	
	Perel[22]	2011	1	1	0	1	0	0	0	1	1	1		Cochrane Database Syst Rev	5,7	
Makani[1]		2013											9	BMJ	17,22	cardiology
	Catapano[2]	2008	0	0	0	0	0	1	1	0	2	1		Am J Kidney Dis	5,29	
	Doulton[4]	2006	0	0	0	1	0	1	1	0	2	1		Hypertension	6,87	
	Harel[6]	2012	1	1	1	1	1	1	1	0	3	1		BMJ	17,22	
	Jennings[5]	2007	0	0	0	0	0	1	1	0	2	1		Diabet Med	3,24	
	Kunz[7]	2008	1	1	0	1	0	1	1	0	2	1		Ann Intern Med	13,98	
	Lakhdar[8]	2008	1	1	1	1	0	1	1	0	2	1		J Card Fail	3,32	
	MacKinnon[3]	2006	0	0	0	0	0	1	1	0	2	1		Am J Kidney Dis	5,29	
Stafford[9]		2013											10	BMJ	17,22	psychiatry
	Marshall[10]	2011	1	1	1	1	1	1	1	1	4	1		Cochrane Database Syst Rev	5,7	
	Preti[11]	2010	0	0	0	1	1	1	1	1	4	1		Schizophr Res	4,59	
Zarychanski[52]		2013											11	JAMA	29,98	anaestesiology
	Gattas[19]	2012	1	1	0	0	0	0	0	0	0	1		Anesth Analg	3,3	

	Perel[22]	2012	1	0	0	1	0	0	0	0	0	1		Cochrane Database Syst Rev	5,7	
	Zarychanski[53]	2009	1	1	0	1	1	1	1	0	3	1		Open Med.	n/a	
Ahuja[149]		2012											n/a	PlosM	15,25	pulmonology
	Johnston[150]	2009	1	0	0	0	1	0	0	0	1	1		PLoS One	3,73	
	Orenstein[151]	2009	1	0	0	0	1	0	0	0	1	1		Lancet Infect Dis	19,97	
Bangalore[127]		2012											8	BMJ	17,22	cardiology
	Boyden[128]	2006	0	0	0	1	1	1	1	0	3	1		Am J Cardiol	3,21	
	de Waha[133]	2011	1	0	0	1	1	1	1	0	3	1		Clin Res Cardiol	3,67	
	Kirtane[129]	2008	0	0	0	1	1	0	1	0	2	1		J Am Coll Cardiol	14,09	
	Mahmud[130]	2008	0	0	0	1	1	1	1	0	3	1		J Am Coll Cardiol	14,09	
	Patti[131]	2008	0	0	0	1	1	1	1	0	3	1		Am J Cardiol	3,21	
	Scheen[135]	2004	1	1	0	1	1	1	1	0	3	1		Eur Heart J	14,1	
	Stettler[132]	2008	1	1	1	1	0	1	1	0	2	1		BMJ	17,22	
	Zhang[134]	2010	0	0	0	1	1	0	0	0	1	1		Am J Cardiol	3,21	
BellemainAppaix[23]		2012											9	JAMA	29,98	cardiology
	Hao[25]	2011	0	0	0	1	1	1	0	1	3	1		J Thromb Haemost	6,08	
	Lotrionte[24]	2007	1	0	0	0	1	1	0	1	3	1		Am J Cardiol	3,21	
	Sabatine[26]	2008	1	1	0	0	1	1	1	1	4	1		Am Heart J	4,5	
	Siller Matula[27]	2010	0	0	0	1	1	1	0	1	3	1		Heart	5,01	
Bischoff-Ferrari[54]		2012											5	NEJM	51,66	orthopedic surgery
	Abrahamsen[63]	2010	1	1	1	0	0	1	1	0	2	1		BMJ	17,22	
	Avenell[62]	2009	1	1	1	0	0	0	0	1	1	1		Cochrane Database Syst Rev	5,7	
	Bergman[55]	2010	0	0	0	1	0	1	0	1	2	1		Curr Med Res Opin	2,26	
	Bischoff-Ferrari[56]	2009	1	1	0	1	1	1	1	1	4	1		JAMA	29,98	
	Boonen[61]	2007	1	1	1	0	0	1	1	0	2	1		J Clin Endocr Metab	6,43	
	Cranney[60]	2007	1	1	1	0	0	0	0	0	0	1		Evid Rep Technol Assess	n/a	
	Izaks[57]	2007	0	0	0	0	0	1	1	1	3	1		BMC Musculoskelet Disord	1,88	
	Lai[58]	2010	0	0	0	0	0	0	1	0	1	1		BMC Public Health	2,08	

	Mosekilde[59]	2007	0	0	0	0	1	1	1	1	4	0		Clin Evid (Online)	n/a	
Boekholdt[210]		2012											7	JAMA	29,98	cardiology
Boussageon[241]		2012											8	PlosM	15,25	diabetology
	Eurich[242]	2007	1	1	0	0	0	0	0	1	1	1		BMJ	17,22	
	Lamanna[243]	2011	1	1	1	0	1	1	0	1	3	1		Diabetes Obes Metab	5,18	
	Saenz Calvo[244]	2005	0	0	0	0	1	1	1	1	4	1		Atención Primaria	0,96	
	Saenz[245]	2005	1	1	1	0	1	1	1	0	3	1		Cochrane Database Syst Rev	5,7	
Caldeira[161]		2012											9	BMJ	17,22	pulmonology
	Rafailidis[162]	2008	1	1	0	1	1	0	1	0	2	0		Eur J Clin Pharmacol	2,74	
	Siempos[163]	2008	1	1	0	0	0	0	1	0	1	1		J Antimicrob Chemoth	5,34	
	Solh[164]	2007	1	1	0	1	0	0	1	0	1	0		Am J Geriatr Pharmac	2,63	
EBCTCG[254]		2012												Lancet	39,06	oncology
	Dowsett[255]	2010	1	0	0	1	0	0	0	0	0	1		J Clin Oncol	18,04	
	EBCTCG[256]	2005	1	0	0	1	1	0	0	0	1	1		Lancet	39,06	
	EBCTCG[257]	2011	1	1	0	0	1	0	0	0	1	1		Lancet	39,06	
Fox[47]		2012											8	BMJ	17,22	cardiology
	Loke[48]	2011	1	1	1	0	1	0	0	0	1	1		J Clin Pharm Ther	2,1	
Gómez -Outes[171]		2012											9	BMJ	17,22	orthopedic surgery
	Cao[172]	2010	0	0	0	0	1	0	1	0	2	1		Eur J Clin Pharmacol	2,74	
	Huang[173]	2011	0	0	0	1	0	0	1	0	1	1		J Thromb Haemost	6,08	
	Loke[48]	2011	1	0	0	0	0	0	1	0	1	1		J Clin Pharm Ther	2,1	
	Maratea[175]	2011	1	1	0	0	0	1	1	0	2	1		J Thromb Haemost	6,08	
	Trkulja[176]	2011	1	0	0	1	1	0	1	0	2	1		Croat Med J	1,25	
	Turun[174]	2011	0	0	0	0	1	0	1	0	2	1		Thromb Res	3,13	
Haas[64]		2012											7	BMJ	17,22	obstetrics & gynecology
	Anotayanonth[72]	2004	1	0	0	1	1	0	0	0	1	1		Cochrane Database Syst Rev	5,7	
	Berkman[65]	2003	0	0	0	0	1	1	1	0	3	1		Am J Obstet Gynecol	3,88	
	Coomarasamy[66]	2002	0	0	0	1	1	0	1	0	2	1		Med Sci Monitor	1,36	

	Crowther[73]	2006	1	0	0	1	0	0	0	0	0	1		Cochrane Database Syst Rev	5,7	
	Haas[67]	2009	1	1	1	1	1	1	1	1	4	1		Obstet Gynecol	4,8	
	King[71]	2005	1	1	1	0	0	0	0	0	0	1		Cochrane Database Syst Rev	5,7	
	King[74]	2003	1	0	0	1	1	0	1	0	2	1		Cochrane Database Syst Rev	5,7	
	Li[68]	2005	0	0	0	0	1	0	0	0	2	1		Indian J Med Res	2,06	
	Papatsonis[75]	2005	1	0	0	0	1	0	0	0	1	1		Cochrane Database Syst Rev	5,7	
	Tan[69]	2006	0	0	0	1	1	1	1	0	3	1		Singap Med J	0,63	
	Yaju[70]	2006	0	0	0	1	1	0	1	0	2	1		Pharmacoepidem Dr S	2,9	
Harel[6]		2012											9	BMJ	17,22	nephrology
Hartling[80]		2012											9	AIM	13,98	psychiatry
	Bhattacharjee[81]	2008	1	0	0	0	0	0	1	0	1	1		Cochrane Database Syst Rev	5,7	
	Crossley[82]	2010	0	0	0	1	0	1	1	0	2	1		Br J Psychiatry	5,95	
	Davis[83]	2003	0	0	0	1	0	0	0	0	0	1		Arch Gen Psychiat	13,77	
	Essali[84]	2009	1	0	0	1	0	0	1	0	1	1		Cochrane Database Syst Rev	5,7	
	Klemp[85]	2011	1	1	1	0	0	0	0	0	0	1		J Clin Psychopharm	3,51	
	Leucht[86]	2003	0	0	0	1	0	1	0	0	1	1		Lancet	39,06	
	Leucht[87]	2009	1	1	1	1	0	0	0	0	0	1		Lancet	39,06	
	Smith[88]	2008	1	0	0	0	0	0	1	0	1	1		Br J Psychiatry	5,95	
Hemmingsen[215]		2012											10	BMJ	17,22	diabetology
Hempel[31]		2012											10	JAMA	29,98	gastroenterology
	Avadhani[29]	2011	0	0	0	1	0	1	1	0	2	1		J Am Acad Nurse Pract	0,71	
	D'Souza[30]	2002	1	0	0	1	1	1	0	1	3	1		BMJ	17,22	
	Johnston[32]	2006	0	0	0	0	0	1	0	1	2	1		CMAJ	6,47	
	Johnston[33]	2011	1	1	0	1	0	1	1	0	2	1		Cochrane Database Syst Rev	5,7	
	McFarland[34]	2006	1	1	0	1	0	1	0	1	2	1		Am J Gastroenterol	7,55	
	Ritchie[35]	2012	0	0	0	1	0	1	0	0	1	1		PLoS One	3,73	
	Sazawal[211]	2006	1	1	0	1	0	1	0	1	2	1		Lancet Infect Dis	19,97	
	Szajewska[37]	2006	1	0	0	1	0	1	0	1	2	1		J Pediatr	4,04	

Hsu[227]		2012											6	AIM	13,98	infectious diseases
	Alves Galvao[237]	2012	1	0	0	1	1	0	1	0	2	1		Cochrane Database Syst Rev	5,7	
	Burch[228]	2009	0	0	0	1	1	1	1	0	3	1		Health Technol Assess	4,03	
	Cooper[229]	2003	0	0	0	1	1	1	1	0	3	1		BMJ	17,22	
	Falagas[230]	2010	0	0	0	1	1	1	0	0	2	1		J Antimicrob Chemoth	5,34	
	Jackson[231]	2011	0	0	0	1	1	1	1	0	3	1		J Infection	4,07	
	Jefferson[232]	2006	0	0	0	0	1	1	1	0	3	1		Lancet	39,06	
	Jefferson[233]	2009	0	0	0	1	1	1	1	0	3	1		BMJ	17,22	
	Jefferson[238]	2010	1	0	0	0	1	1	0	0	2	1		Cochrane Database Syst Rev	5,7	
	Jefferson[239]	2012	1	1	1	0	1	1	0	0	2	1		Cochrane Database Syst Rev	5,7	
	Khazeni[234]	2009	0	0	0	1	0	1	0	0	1	1		JAMA Internal Medicine	11,46	
	Shun-Shin[235]	2009	0	0	0	1	0	1	0	0	1	1		BMJ	17,22	
	Tappenden[236]	2009	0	0	0	1	1	1	1	0	3	1		Health Technol Assess	4,03	
	Wang[240]	2012	1	0	0	1	0	1	1	0	2	1		Cochrane Database Syst Rev	5,7	
Huedo-Medina[42]		2012											8	BMJ	17,22	psychiatry
	Buscemi[43]	2007	1	0	0	1	0	0	1	1	2	1		J Gen Intern Med.	3,28	
	Dundar[44]	2004	1	0	0	1	1	1	0	1	3	1		Health Technol Assess	4,03	
	Dundar[45]	2009	1	0	0	1	1	1	0	1	3	1		Hum Psychopharmacol	2,1	
	Glass[46]	2005	1	1	0	1	0	1	1	1	3	1		BMJ	17,22	
Hutton[89]		2012											6	BMJ	17,22	cardiology
	Brown[93]	2007	1	1	1	1	1	1	0	0	2	1		Circulation	15,2	
	McIlroy[90]	2009	0	0	0	0	0	1	1	0	2	1		Brit J Anaesth	4,24	
	Ngaage[91]	2010	0	0	0	1	1	0	0	0	1	1		Eur J Cardiothorac Surg	2,65	
	Umscheid[92]	2007	0	0	0	0	0	1	1	0	2	1		Curr Opin Hematol	4,11	
Jackson[212]		2012											9	JAMA	29,98	neurology
	Gupta[213]	2006	0	0	0	0	0	1	1	1	3	0		Pain Med.	2,46	
	Shuhendler[214]	2010	0	0	0	0	0	1	0	1	2	1		Pharmacotherapy	2,31	
Jardine[177]		2012											10	BMJ	17,22	nephrology
	Bazzano[178]	2006	1	1	1	1	0	0	0	0	0	1		JAMA	29,98	

	Clarke[179]	2010	1	1	1	1	0	0	0	0	0	1		JAMA Internal Medicine	11,46	
	Qin[188]	2011	1	1	1	0	0	1	1	0	2	1		Clin J Am Soc Nephrol	5,07	
	Wang[181]	2007	1	1	1	0	0	0	0	0	0	1		Lancet	39,06	
Johnston[28]		2012											9	AIM	13,98	gastroenterology
	Avadhani[29]	2011	0	0	0	1	0	1	1	1	3	1		J Am Acad Nurse Pract	0,71	
	Dendukuri[38]	2005	1	0	0	0	1	1	1	1	4	0		CMAJ	6,47	
	D'Souza[30]	2002	0	0	0	0	1	1	1	0	3	1		BMJ	17,22	
	Hempel[31]	2012	1	1	1	1	1	1	0	0	2	1		JAMA	29,98	
	Johnston[32]	2006	0	0	0	0	0	1	1	0	2	1		CMAJ	6,47	
	Johnston[33]	2011	1	0	0	1	0	1	1	0	2	1		Cochrane Database Syst Rev	5,7	
	McFarland[34]	2006	0	0	0	1	1	1	1	0	3	1		Am J Gastroenterol	7,55	
	Ritchie[35]	2009	0	0	0	1	1	1	1	1	3	1		PLoS One	3,73	
	Segarra[36]	2007	0	0	0	0	1	1	1	1	4	1		Ann Pharmacother	2,57	
	Szajewska[37]	2006	0	0	0	1	0	1	1	0	2	1		J Pediatr	4,04	
Karagiannis[155]		2012											10	BMJ	17,22	diabetology
	Esposito[156]	2011	1	0	0	1	1	1	0	0	2	1		Diabetes Obes Metab	5,18	
	Monami[157]	2010	1	1	1	0	1	1	0	0	2	1		Nutr Metab Cardiovasc Dis	3,98	
	Richter[158]	2008	1	1	1	0	1	1	0	0	2	1		Cochrane Database Syst Rev	5,7	
	Richter[159]	2008	0	0	0	0	1	1	0	0	2	1		Vasc Health Risk Manag	1,6	
	Wani[160]	2008	0	0	0	0	1	1	0	0	2	0		Cardiol Clin	1,32	
Ker[184]		2012											9	BMJ	17,22	general surgery
	Abrishami[206]	2009	0	0	0	1	0	0	0	0	0	1		can j anaesth	2,35	
	Adler[185]	2011	1	0	0	1	0	1	1	1	3	1		J Cardiothor Vasc An	1,45	
	Alshryda[186]	2011	1	0	0	1	0	1	1	1	3	1		J Bone Joint Surg Br	2,74	
	Brown[93]	2007	1	0	0	1	0	0	1	0	1	1		Circulation	15,2	
	Carless[207]	2005	0	0	0	1	0	0	0	0	0	1		Bmc Cardiovasc Disor	1,46	
	Cid[187]	2005	1	0	0	1	0	1	0	0	1	1		Transfusion	3,53	
	Elgafy[188]	2010	1	0	0	1	0	0	0	0	0	0		SPINE	2,16	
	Gill[189]	2006	1	0	0	0	0	0	0	0	0	1		J Arthroplasty	2,11	

	Gill[190]	2008	1	0	0	1	0	0	0	0	0	1		J Bone Joint Surg Am	3,23	
	Guay[191]	2006	1	0	0	1	0	0	0	0	0	0		Can J Anaesth	2,35	
	Gurusamy[192]	2009	1	0	0	1	0	0	1	0	1	1		Cochrane Database Syst Rev	5,7	
	Ho[193]	2003	1	0	0	1	0	1	0	0	1	1		Anaesth Intensive Care	1,4	
	Kagoma[194]	2009	1	0	0	1	0	0	1	1	2	1		Thromb Res	3,13	
	Kongnyuy[195]	2009	1	0	0	1	0	0	1	0	1	1		Cochrane Database Syst Rev	5,7	
	Liu[196]	2010	1	0	0	1	0	1	0	1	2	1		Zhonghua Wai Ke Za Zhi	n/a	
	Makwana[197]	2010	1	0	0	1	0	0	1	0	1	0		Indian Journal of Anaesthesia	n/a	
	Martin-Hirsch[198]	2010	1	0	0	1	0	0	1	0	1	1		Cochrane Database Syst Rev	5,7	
	McIlroy[90]	2009	0	0	0	1	0	0	0	0	0	1		Brit J Anaesth	4,24	
	Molenaar[199]	2007	1	0	0	1	0	0	1	0	1	1		Am J Transplant	6,19	
	Ngaage[91]	2010	1	0	0	1	0	1	0	0	1	1		Eur J Cardiothorac Surg	2,65	
	Novikova[200]	2010	1	0	0	1	0	1	1	0	2	1		Cochrane Database Syst Rev	5,7	
	Schouten[201]	2009	1	0	0	1	0	0	0	0	0	1		Pediatr Crit Care Me	2,35	
	Sukeik[202]	2011	1	0	0	1	0	1	1	0	2	1		J Bone Joint Surg Br	2,74	
	Takagi[208]	2009	0	0	0	1	0	1	0	0	1	1		Int Cardiovasc Thorac Surg	2,11	
	Tzortzopoulou[203]	2008	1	0	0	1	0	0	0	0	0	1		Cochrane Database Syst Rev	5,7	
	Umscheid[92]	2007	1	0	0	1	0	0	0	0	0	1		Curr Opin Hematol	4,11	
	Zhang[204]	2011	1	0	0	1	0	1	0	0	1	1		Knee Surg Sports Traum Arthro	2,68	
	Zufferey[205]	2006	1	0	0	1	0	0	0	0	0	1		Anesthesiology	5,16	
Leucht[182]		2012											11	Lancet	39,06	psychiatry
	Álvarez-Jiménez[183]	2011	0	0	0	1	0	0	1	0	1	1		Schizophrenia Bull	8,49	
	Leucht[191]	2003	1	0	0	1	1	0	0	0	1	1		Am J Psychiatry	14,72	
Lopez-Olivo[94]		2012											6	JAMA	29,98	oncology
	Askling[96]	2011	1	1	0	0	0	0	1	1	2	1		Pharmacoepidem Dr S	2,9	
	Bongartz[95]	2006	1	1	1	0	1	0	0	0	1	1		JAMA	29,98	
	Bongartz[97]	2009	1	0	0	0	1	0	1	1	3	1		Ann Rheum Dis	9,11	
	Kaiser[100]	2008	1	0	0	1	1	0	0	0	1	0		Clin Lymphoma Myeloma	1,67	

	Le Blay[101]	2012	1	0	0	1	1	0	0	0	1	1		J Rheumatol	3,26	
	Leombruno[102]	2009	1	0	0	1	1	0	1	0	2	1		Ann Rheum Dis	9,11	
	Nannini[103]	2009	1	1	0	0	0	0	0	1	1	0		Arthritis Rheum	7,48	
	Salliot[98]	2009	0	0	0	1	1	0	1	0	2	0		Ann Rheum Dis	9,11	
	Thompson[99]	2011	0	0	0	1	0	0	1	0	1	1		Arthritis Rheum	7,48	
	Wiens[104]	2009	1	0	0	1	1	0	0	0	1	1		Clin Rheumatol	2,04	
	Wiens[105]	2009	1	0	0	1	1	0	0	0	1	1		Scand J Immunol	2,2	
	Wiens[106]	2010	1	0	0	1	1	0	0	0	1	1		Rheumatol Int	2,21	
	Wiens[107]	2010	1	0	0	1	1	0	0	0	1	1		Pharmacotherapy	2,31	
	Wong[108]	2012	1	0	0	0	1	0	1	0	2	1		Clin Rheumatol	2,04	
Low[49]		2012											9	BMJ	17,22	sports medicine
	Seupaul[50]	2012	1	1	1	1	1	0	1	1	3	0		Ann Emerg Med.	4,29	
	Kayser[51]	2012	1	1	1	1	1	1	1	1	4	1		High Alt Med Biol	2,12	
Lv[152]		2012											9	PlosM	15,25	nephrology
	Arguedas[154]	2009	1	1	1	1	1	0	0	0	1	1		Cochrane Database Syst Rev	5,7	
	Reboldi[153]	2011	1	1	1	1	0	1	1	0	2	1		J Hypertens	3,81	
MacArthur[76]		2012											10	BMJ	17,22	infectious diseases
	Gowing[78]	2011	1	1	0	1	1	1	1	1	4	1		Cochrane Database Syst Rev	5,7	
	Heidrich[77]	2011	1	0	0	1	0	0	1	1	2	0		Addiction	4,58	
	Larney[79]	2010	1	0	0	1	0	1	1	1	3	0		Addiction	4,58	
Mantha[136]		2012											6	BMJ	17,22	obstetrics & gynecology
	Bergendal[137]	2009	1	0	0	0	1	1	1	1	4	1		Acta Obstet Gyn Scan	1,85	
McKnight[247]		2012											8	Lancet	39,06	psychiatry
	Ceron-Litvoc[248]	2009	0	0	0	0	0	1	0	0	1	1		Hum Psychopharmacol	2,1	
	Paul[249]	2010	1	1	0	1	0	1	1	0	2	1		J Psychopharmacol	3,37	
Mihaylova[124]		2012											4	Lancet	39,06	angiology
	CTT[125]	2010	1	1	1	1	0	0	1	0	1	1		Lancet	39,06	
	Taylor[126]	2011	1	1	0	1	1	1	1	0	3	1		Cochrane Database Syst Rev	5,7	

Neumann[209]		2012											10	AIM	13,98	orthopedic surgery
	Cao[172]	2010	1	1	0	0	1	0	0	0	1	1		Eur J Clin Pharmacol	2,74	
	Huang[173]	2011	1	1	1	0	0	0	0	0	0	1		J Thromb Haemost	6,08	
	Turun[174]	2011	1	1	1	0	1	0	0	0	1	1		Thromb Res	3,13	
Palmer[118]		2012											9	AIM	13,98	nephrology
	Navaneethan[119]	2009	1	0	0	1	0	1	1	0	2	1		Cochrane Database Syst Rev	5,7	
	Navaneethan[120]	2009	1	0	0	1	0	1	1	0	2	1		Cochrane Database Syst Rev	5,7	
	Navaneethan[121]	2009	1	0	0	1	0	1	1	0	2	1		Cochrane Database Syst Rev	5,7	
	Strippoli[122]	2008	1	1	0	0	1	1	1	0	3	1		BMJ	17,22	
Palmer[246]		2012											8	AIM	13,98	nephrology
Palmerini[216]		2012											9	Lancet	39,06	cardiology
	Baber[218]	2011	0	0	0	1	1	0	0	0	1	1		J Am Coll Cardiol	14,09	
	de Waha[133]	2011	1	1	1	1	0	0	0	0	0	1		Clin Res Cardiol	3,67	
	Mauri[220]	2007	1	1	0	0	1	0	0	0	1	1		New Engl J Med.	51,66	
	Milic[217]	2010	0	0	0	0	1	1	0	0	2	1		Chin Med J (Engl)	0,9	
	Roukoz[219]	2009	0	0	0	0	1	1	0	0	2	1		Am J Med	4,77	
	Stettler[132]	2007	1	1	1	0	1	0	0	0	1	1		Lancet	39,06	
Pinto[250]		2012											9	BMJ	17,22	neurology
	Chou[252]	2007	1	0	0	0	0	0	1	0	1	0		JAMA Internal Medicine	11,46	
	Deshpande[251]	2010	0	0	0	1	0	0	0	0	0	1		Cochrane Database Syst Rev	5,7	
	Luijsterburg[40]	2007	1	0	0	1	0	0	1	0	1	0		Eur Spine J	2,13	
	Roelofs[253]	2010	1	0	0	0	0	0	0	0	0	1		Cochrane Database Syst Rev	5,7	
Pinto[39]		2012											8	AIM	13,98	neurology
	Luijsterburg[40]	2007	1	1	0	0	1	0	0	0	1	0		Eur Spine J	2,13	
	Quraishi[41]	2012	1	0	0	1	1	0	1	0	2	1		Eur Spine J	2,13	
Plante[113]		2012											9	BMJ	17,22	otolaryngology
	Diakos[115]	2011	0	0	0	1	0	0	1	0	1	1		Clin Otolaryngol	1,87	
	Geva[114]	2011	1	1	1	1	1	0	1	0	2	1		Otolaryngol Head Neck Surg	1,63	

Preiss[116]		2012											9	JAMA	29,98	gastroenterology
	Singh[117]	2006	1	1	0	0	1	0	0	1	2	1		Drug Safety	3,41	
Rahimi[109]		2012											9	PlosM	15,25	cardiology
	Agarwal[110]	2010	0	0	0	0	1	0	0	1	2	1		Int J Clin Pract	2,43	
	Pai[111]	2011	0	0	0	0	1	0	0	1	2	1		Thromb Res	3,13	
	Squizzato[112]	2010	0	0	0	0	1	0	0	1	2	1		Eur Heart J	14,1	
Rutjes[138]		2012											7	AIM	13,98	rheumatology
	Aggarwal[139]	2004	0	0	0	0	1	1	0	0	2	0		Can Fam Physician	1,81	
	Arrich[143]	2005	1	1	1	1	1	1	1	0	3	1		CMAJ	6,47	
	Bannuru[144]	2011	1	1	1	0	1	1	1	0	3	1		Osteoarthr Cartilage	4,26	
	Bellamy[145]	2006	1	1	1	0	1	1	0	0	2	1		Cochrane Database Syst Rev	5,7	
	Conrozier[140]	2005	0	0	0	0	1	0	0	0	1	0		Clin Exp Rheumatol	2,66	
	Divine[141]	2006	0	0	0	0	1	1	1	0	3	0		Clin Orthop Relat R	2,79	
	Lo[142]	2003	1	0	0	1	1	1	1	0	3	1		JAMA	29,98	
	Medina[146]	2006	1	1	1	1	1	1	1	0	3	1		J Fam Pract	0,67	
	Modawal[147]	2005	1	1	1	0	1	1	1	0	3	1		J Fam Pract	0,67	
	Wang[148]	2004	1	1	1	0	1	1	1	0	3	1		J Bone Joint Surg Am	3,23	
Silvain[258]		2012											11	BMJ	17,22	cardiology
	Borentain[259]	2005	0	0	0	0	1	1	1	0	3	1		Catheter Cardio Inte	2,51	
	Dumaine[260]	2007	1	1	0	1	1	0	1	0	2	1		JAMA Internal Medicine	11,46	
	Navarese[261]	2011	1	1	1	1	0	1	1	1	3	1		J Thromb Haemost	6,08	
Suthar[165]		2012											10	PlosM	15,25	infectious diseases
	Lawn[166]	2010	1	1	1	1	1	1	1	1	4	1		Lancet Infect Dis	19,97	
Upadhyay[123]		2012											8	AIM	13,98	nephrology
	Navaneethan[119]	2009	1	1	1	1	0	0	1	0	1	1		Cochrane Database Syst Rev	5,7	
	Navaneethan[120]	2009	1	1	1	1	0	0	1	0	1	1		Cochrane Database Syst Rev	5,7	
	Navaneethan[121]	2009	0	0	0	1	0	0	1	0	1	1		Cochrane Database Syst Rev	5,7	
	Strippoli[122]	2008	1	1	1	1	0	0	1	0	1	1		BMJ	17,22	

Varadhan[221]		2012											9	BMJ	17,22	general surgery
	Ansaloni[222]	2011	1	1	1	0	1	1	1	0	3	1		DIGEST SURG	1,47	
	Fitzmaurice[223]	2011	1	1	0	0	1	1	1	0	3	0		CAN J SURG	1,63	
	Liu[224]	2011	1	1	1	0	1	1	1	0	3	1		Surgery	3,37	
	Varadhan[225]	2010	1	1	0	0	1	1	1	0	3	1		World J Surg	2,23	
	Wilms[226]	2011	1	1	1	0	1	1	1	0	3	1		Cochrane Database Syst Rev	5,7	
Vilsboll[262]		2012											8	BMJ	17,22	internal medicine
	Amori[263]	2007	0	0	0	1	0	0	0	0	0	1		JAMA	29,98	
	Monami[264]	2009	0	0	0	1	0	1	1	0	2	1		Eur J Endocrinol	3,14	
	Norris[265]	2009	0	0	0	1	0	0	1	0	1	1		Diabet Med	3,24	
	Shyangdan[266]	2010	0	0	0	1	0	1	1	0	2	1		Bmc Endocr Disord	2,65	
	Shyangdan[267]	2011	0	0	0	1	0	1	1	0	2	1		Cochrane Database Syst Rev	5,7	
Wardlaw[167]		2012											7	Lancet	39,06	neurology
	Bhatnagar[168]	2011	1	1	0	0	0	0	1	0	1	1		J Neurol Neurosurg Psychiatry	4,92	
	Wardlaw[169]	2003	1	1	0	1	1	1	1	1	4	1		Stroke	6,16	
	Wardlaw[170]	2009	1	1	0	1	0	0	1	0	1	1		Cochrane Database Syst Rev	5,7	
