# Supplementary material for: Recent meta-analyses neglect previous systematic reviews and meta-analyses about the same topic: a systematic examination
Source: BMC Med. 2015 Apr 14;13:82. doi: 10.1186/s12916-015-0317-4 (PMC4411715; doi:10.1186/s12916-015-0317-4)
Supplement: Additional file 6: — Detailed characteristics of recent meta-analyses. [file 12916_2015_317_MOESM6_ESM.pdf]

Additional file 6

Detailed characteristics of recent meta-analyses.

Ahuja[149]
Topic: multidrug resistant pulmonary tuberculosis treatment
People: people with multidrug resistant tuberculosis
Intervention: different drug classes
Comparison: different drug classes
Outcome: treatment success, compared to failure or relapse

Bangalore[127]
Topic: outcomes with various drug eluting or bare metal stents
People: patients with diabetes
Intervention: drug eluting stents
Comparison: drug eluting stents or bare metal stents
Outcome: efficacy (target vessel revascularization) and safety (death, myocardial infarction, stent thrombosis)

Bellemain-Appaix[23]
Topic: clopidogrel pretreatment
People: patients scheduled for percutaneous coronary intervention
Intervention: clopidogrel
Comparison: placebo, no treatment
Outcome: mortality and major bleeding

Bischoff-Ferrari[54]
Topic: vitamin D for fracture prevention
People: 65 or older
Intervention: vitamin D with or without calcium
Comparison: placebo or calcium
Outcome: hip and nonvertebral fractures

Boekholdt[210]
Topic: risk of cardiovascular events and statin treatment
People: patients treated with statin
Intervention: statins
Comparison: LDL, non–HDL cholesterol, and apolipoprotein B levels
Outcome: risk of major cardiovascular events

Boussageon[241]
Topic: metformin efficacy in the treatment of type 2 diabetes
People: people with type 2 diabetes
Intervention: metformin
Comparison: diet alone, placebo, no treatment; metformin as an add-on therapy; and metformin withdrawal
Outcome: cardiovascular morbidity or mortality

Caldeira[161]
Topic: pneumonia associated with use of angiotensin converting enzyme inhibitors and angiotensin receptor blockers
People: all
Intervention: of angiotensin converting enzyme inhibitors and angiotensin receptor blockers
Comparison: control treatment
Outcome: incidence of pneumonia and the secondary outcome was pneumonia related mortality

Chatterjee[12]
Topic: beta blockers in patients with heart failure and reduced ejection fraction
People: patients with heart failure and reduced ejection fraction
Intervention: beta blocker
Comparison: another beta blocker, standard treatment or placebo
Outcome: all cause death

EBCTCG[254]
Topic: polychemotherapy regimens for early breast cancer

	People:	women with early breast cancer
	Intervention:	any taxane-plusanthracycline-based regimen or one anthracyclinebased regimen versus another or polychemotherapy
	Comparison:	any taxane-plusanthracycline-based regimen or one anthracyclinebased regimen versus another or polychemotherapy or non-taxane chemotherapy or versus cyclo phosphamide, methotrexate, and fluorouracil or no chemotherapy
	Outcome:	breast cancer mortality rate ratios
**Fox**[47]	Topic:	oral anticoagulants for treatment of acute venous thromboembolism
	People:	people with acute venous thromboembolism
	Intervention:	oral anticoagulants (rivaroxaban, dabigatran, ximelagatran, and apixaban)
	Comparison:	other drugs and vitamin K antagonists
	Outcome:	recurrent events, major bleeding, and all-cause mortality
**Gómez-Outes**[171]	Topic:	dabigatran, rivaroxaban, apixaban
	People:	people with venous thromboembolism after total hip or knee replacement
	Intervention:	dabigatran, rivaroxaban or apixaban
	Comparison:	enoxaparin
	Outcome:	relative risks of symptomatic venous thromboembolism, clinically relevant bleeding, deaths, and a net clinical endpoint
**Haas**[64]	Topic:	tocolytic therapy for preterm delivery
	People:	women at risk of preterm delivery
	Intervention:	tocolytic agents
	Comparison:	placebo
	Outcome:	48 hour delay in delivery, respiratory distress syndrome, neonatal mortality and all-cause maternal side effects
**Haase**[16]	Topic:	hydroxyethyl starch 130/0.38-0.45 versus crystalloid or albumin in patients with sepsis
	People:	patients with sepsis
	Intervention:	hydroxyethyl starch 130/0.38-0.45
	Comparison:	crystalloid or albumin
	Outcome:	mortality, kidney injury, bleeding, and serious adverse events
**Harel**[6]	Topic:	aliskiren and blockers of the renin-angiotensin system in acute kidney injury
	People:	all
	Intervention:	aliskiren and blockers of the renin-angiotensin system
	Comparison:	monotherapy
	Outcome:	hyperkalaemia and acute kidney injury
**Hartling**[80]	Topic:	antipsychotics in adults with schizophrenia
	People:	adults with schizophrenia 18-64yrs
	Intervention:	first- and second-generation antipsychotics
	Comparison:	first- and second-generation antipsychotics
	Outcome:	illness symptoms, diabetes mellitus, mortality, tardive dyskinesia, and a major metabolic syndrome
**Hemmingsen**[215]	Topic:	metformin and insulin versus insulin alone for type 2 diabetes
	People:	people with type 2 diabetes
	Intervention:	metformin and insulin
	Comparison:	insulin with or without placebo
	Outcome:	all-cause mortality, cardiovascular mortality

Hempel[31]

**Hempel**[31]	Topic:	probiotics for the prevention and treatment of antibiotic associated diarrhea (AAD)
	People:	people with AAD
	Intervention:	probiotics (Lactobacillus, Bifidobacterium, Saccharomyces, Streptococcus, Enterococcus, and/or Bacillus)
	Comparison:	no treatment, placebo, or a different probiotic
	Outcome:	reduction in AAD
**Hsu**[227]	Topic:	antivirals for treatment of influenza
	People:	people with influenza or influenza-like illness
	Intervention:	oseltamivir, zanamivir, amantadine, or rimantadine
	Comparison:	no therapy or other antiviral therapy, or no comparator
	Outcome:	mortality, hospitalization, duration of symptoms
**Huedo-Medina**[42]	Topic:	non-benzodiazepine hypnotics in treatment insomnia
	People:	adults with insomnia
	Intervention:	non-benzodiazepine hypnotics
	Comparison:	placebo
	Outcome:	efficacy (change scores, sleep latency)
**Hutton**[89]	Topic:	antifibrinolytics in cardiac surgery
	People:	patients undergoing cardiac surgery
	Intervention:	antifibrinolytics
	Comparison:	no treatment
	Outcome:	risks of death, myocardial infarction, stroke, and renal failure or dysfunction
**Jackson**[212]	Topic:	botulinum toxin, migraine and tension headaches
	People:	adults with headaches (episodic or chronic) or migraines
	Intervention:	botulinum toxin A
	Comparison:	placebo or other intervention
	Outcome:	incidence of headaches
**Jardine**[177]	Topic:	folic acid based homocysteine lowering and cardiovascular
	People:	people with chronic kidney disease or transplant, without dialysis
	Intervention:	folic acid based homocysteine
	Comparison:	placebo or usual care or lower vs. higher dose
	Outcome:	cardiovascular events (myocardial infarction, stroke, and cardiovascular mortality, access thrombosis, requirement for renal replacement therapy) and reported adverse events, including haematological and neurological events
**Johnston**[28]	Topic:	probiotics for the prevention of clostridium difficile-associated diarrhea (CDAD)
	People:	adult or pediatric patients receiving antibiotics
	Intervention:	probiotics for prevention
	Comparison:	placebo or no treatment
	Outcome:	incidence of CDAD
**Karagiannis**[155]	Topic:	dipeptidyl peptidase-4 inhibitors for treatment of diabetes
	People:	adults with type 2 diabetes mellitus
	Intervention:	dipeptidyl peptidase-4 (DPP-4)
	Comparison:	metformin as monotherapy or other commonly used hypoglycaemic drugs combined with metformin
	Outcome:	change in baseline in glycated haemoglobin, body weight, discontinuation rate because of any adverse event, occurrence of any serious adverse event, all-cause mortality, and incidence of hypoglycaemia, nasopharyngitis, urinary tract

		infection, upper respiratory infection, nausea, vomiting, and diarrhea
**Ker**[184]	Topic:	tranexamic acid and surgical bleeding
	People:	surgical patients
	Intervention:	tranexamic acid
	Comparison:	no tranexamic acid or placebo
	Outcome:	blood transfusion, thromboembolic events
**Leucht**[182]	Topic:	Antipsychotic drugs for relapse prevention in schizophrenia
	People:	people with schizophrenia
	Intervention:	antipsychotic drugs
	Comparison:	placebo
	Outcome:	relapse prevention
**Lopez-Olivo**[94]	Topic:	malignancies in patients with rheumatoid arthritis
	People:	patients with rheumatoid arthritis
	Intervention:	biologic response modifiers
	Comparison:	other disease-modifying antirheumatic drugs or with placebo
	Outcome:	risk of malignancies
**Low**[49]	Topic:	acetazolamide for the prophylaxis of acute mountain sickness
	People:	all
	Intervention:	acetazolamide for prevention
	Comparison:	placebo
	Outcome:	event rates
**Lv**[152]	Topic:	intensive blood pressure (BP) lowering
	People:	all
	Intervention:	intensive BP lowering regimens
	Comparison:	standard regimens
	Outcome:	vascular eye and renal outcomes
**MacArthur**[76]	Topic:	opiate substitution treatment and HIV
	People:	people who inject drugs
	Intervention:	opiate substitution treatment
	Comparison:	no treatment or methadone
	Outcome:	incidence of HIV
**Makani**[1]	Topic:	dual blockade of the renin-angiotensin system
	People:	humans
	Intervention:	dual blockade
	Comparison:	monotherapy
	Outcome:	long term efficacy (all-cause mortality, cardiovascular mortality, and admissions to hospital for heart failure), safety events (hyperkalemia, hypotension, renal failure, and withdrawal owing to drug related adverse events)
**Mantha**[136]	Topic:	risk of venous thromboembolic events
	People:	women taking progestin-only contraception
	Intervention:	progestin-only contraception
	Comparison:	non-hormone comparator group
	Outcome:	risk of venous thromboembolic events
**McKnight**[247]	Topic:	lithium toxicity profile
	People:	people with bipolar or mood disorders
	Intervention:	lithium
	Comparison:	placebo, no treatment or other drugs
	Outcome:	adverse events

Mihaylova[124]
Topic: lowering LDL cholesterol with statin therapy
People: people at low risk of vascular disease
Intervention: statins
Comparison: control treatment or placebo
Outcome: coronary vascular stroke cancer mortality

Neumann[209]
Topic: oral direct factor xa inhibitors vs. low-molecular-weight heparin
People: patients undergoing total hip or knee replacement
Intervention: oral direct factor Xa inhibitor
Comparison: low-molecular-weight heparin
Outcome: mortality at the end of prophylaxis, mortality during the follow-up period, symptomatic DVT, nonfatal pulmonary embolism, major bleeding (as defined by the investigators on each trial), intracranial bleeding

Palmer[118]
Topic: statin therapy for persons with chronic kidney disease
People: adults with chronic kidney disease
Intervention: statins
Comparison: placebo, no treatment, or another statin
Outcome: mortality and cardiovascular outcomes

Palmer[246]
Topic: antiplatelet therapy in chronic kidney disease
People: people with chronic kidney disease
Intervention: antiplatelet therapy
Comparison: standard care, placebo, or no treatment
Outcome: mortality and cardiovascular and bleeding outcomes

Palmerini[216]
Topic: stent thrombosis with drug-eluting and bare-metal stents
People: all
Intervention: drug-eluting stents
Comparison: drug eluting stents or bare-metal stents
Outcome: risk of thrombosis

Pinto[39]
Topic: epidural corticosteroid injections in the management of sciatica
People: people with sciatica
Intervention: epidural corticosteroid injection
Comparison: placebo
Outcome: pain reduction

Pinto[250]
Topic: drugs for relief of pain in sciatica
People: patients with sciatica
Intervention: single, combination or adjuvant: non-steroidal anti-inflammatory drugs (NSAIDs), corticosteroids, antidepressants, anticonvulsants, muscle relaxants, and opioid analgesics
Comparison: placebo or other treatment
Outcome: pain intensity, disability status, work status, and adverse events

Plante[113]
Topic: effect of systemic steroids on post-tonsillectomy
People: patients undergoing tonsillectomy
Intervention: systemic steroids
Comparison: any other drug
Outcome: risk of postoperative bleeding and reintervention

Preiss[116]
Topic: lipid-modifying therapies and risk of pancreatitis
People: adult patients
Intervention: statin or fibrate therapy

	Comparison:	placebo or treatment as usual
	Outcome:	incidents of pancreatitis
**Rahimi**[109]	Topic:	statins and venous thromboembolic events
	People:	all
	Intervention:	statin or high dose
	Comparison:	no satin or standard dose
	Outcome:	risk of venous thromboembolic events
**Rutjes**[138]	Topic:	viscosupplementation for osteoarthritis of the knee
	People:	adults with symptomatic knee osteoarthritis
	Intervention:	viscosupplementation
	Comparison:	sham or nonintervention control
	Outcome:	pain intensity and flare-ups adverse events
**Silvain**[258]	Topic:	enoxaparin versus unfractionated heparin during percutaneous coronary intervention
	People:	patients having percutaneous coronary intervention
	Intervention:	enoxaparin
	Comparison:	unfractionated heparin
	Outcome:	mortality and major bleeding
**Stafford**[9]	Topic:	early interventions to prevent psychosis
	People:	people at high risk of psychosis
	Intervention:	any psychological, pharmacological, nutritional or combined
	Comparison:	usual services or another treatment
	Outcome:	transition to psychosis, symptoms of psychosis, depression, and mania; quality of life; weight; and discontinuation of treatment
**Suthar**[165]	Topic:	antiretroviral therapy for prevention of tuberculosis
	People:	adults with HIV
	Intervention:	antiretroviral therapy
	Comparison:	no antiretroviral drug
	Outcome:	incidence of tuberculosis
**Upadhyay**[123]	Topic:	lipid-lowering therapy
	People:	persons with chronic kidney disease
	Intervention:	lipid-lowering therapy
	Comparison:	control treatment or placebo
	Outcome:	cardiovascular and kidney outcomes, and adverse events
**Varadhan**[221]	Topic:	antibiotics compared with appendicectomy for treatment of uncomplicated acute appendicitis
	People:	people with uncomplicated acute appendicitis
	Intervention:	antibiotics
	Comparison:	appendicectomy
	Outcome:	complications, efficacy of treatment, length of stay, and incidence of complicated appendicitis and readmissions
**Vilsboll**[262]	Topic:	glucagon-like peptide-1 receptor agonists and weight loss
	People:	overweight or obese patients (body mass index of 25 or higher) with or without type 2 diabetes mellitus.
	Intervention:	glucagon-like peptide-1 receptor agonists (exenatide, liraglutide)
	Comparison:	placebo, oral antidiabetic drugs, or insulin
	Outcome:	weight loss
**Wardlaw**[167]	Topic:	recombinant tissue plasminogen activator (rt-PA)
	People:	people within 6 h of onset of acute ischaemic stroke

	Intervention:	intravenous rt-PA
	Comparison:	control
	Outcome:	odds of being alive, benefit
**Zarychanski**[52]	Topic:	hydroxyethyl starch in critically ill requiring volume resuscitation
	People:	critically ill
	Intervention:	hydroxyethyl starch
	Comparison:	crystalloids, albumin, or gelatin
	Outcome:	mortality and acute kidney injury
