# Supplementary material for: Recent meta-analyses neglect previous systematic reviews and meta-analyses about the same topic: a systematic examination
Source: BMC Med. 2015 Apr 14;13:82. doi: 10.1186/s12916-015-0317-4 (PMC4411715; doi:10.1186/s12916-015-0317-4)
Supplement: Additional file 7: — References to all included meta-analyses and systematic reviews. [file 12916_2015_317_MOESM7_ESM.pdf]

Additional file 7

References to all included meta-analyses and systematic reviews

1. Makani H, Bangalore S, Desouza KA, Shah A, Messerli FH. Efficacy and safety of dual blockade of the renin-angiotensin system: meta-analysis of randomised trials. BMJ. 2013 Jan 28;346:f360.

2. Catapano F, Chiodini P, De Nicola L, et al. Antiproteinuric response to dual blockade of the renin-angiotensin system in primary glomerulonephritis: meta-analysis and metaregression. Am J Kidney Dis. 2008 Sep;52(3):475-85.

3. MacKinnon M, Shurraw S, Akbari A, Knoll GA, Jaffey J, Clark HD. Combination therapy with an angiotensin receptor blocker and an ACE inhibitor in proteinuric renal disease: a systematic review of the efficacy and safety data. Am J Kidney Dis. 2006 Jul;48(1):8-20.

4. Doulton TW, He FJ, MacGregor GA. Systematic review of combined angiotensin-converting enzyme inhibition and angiotensin receptor blockade in hypertension. Hypertension. 2005 May;45(5):880-6. Epub 2005 Apr 4.

5. Jennings DL, Kalus JS, Coleman CI, Manierski C, Yee J. Combination therapy with an ACE inhibitor and an angiotensin receptor blocker for diabetic nephropathy: a metaanalysis. Diabet Med. 2007 May;24(5):486-93. Epub 2007 Mar 15.

6. Harel Z, Gilbert C, Wald R, et al. The effect of combination treatment with aliskiren and blockers of the renin-angiotensin system on hyperkalaemia and acute kidney injury: systematic review and meta-analysis. BMJ. 2012 Jan 9;344:e42. doi: 10.1136/bmj.e42.

7. Kunz R, Friedrich C, Wolbers M, Mann JF. Meta-analysis: effect of monotherapy and combination therapy with inhibitors of the renin angiotensin system on proteinuria in renal disease. Ann Intern Med. 2008 Jan 1;148(1):30-48. Epub 2007 Nov 5.

8. Lakhdar R, Al-Mallah MH, Lanfear DE. Safety and tolerability of angiotensin-converting enzyme inhibitor versus the combination of angiotensin-converting enzyme inhibitor and angiotensin receptor blocker in patients with left ventricular dysfunction: a systematic review and meta-analysis of randomized controlled trials. J Card Fail. 2008 Apr;14(3):181-8. doi: 10.1016/j.cardfail.2007.11.008.

9. Stafford MR, Jackson H, Mayo-Wilson E, Morrison AP, Kendall T. Early interventions to prevent psychosis: systematic review and meta-analysis. BMJ. 2013 Jan 18;346:f185. doi: 10.1136/bmj.f185.

10. Marshall M, Rathbone J. Early intervention for psychosis. Cochrane Database Syst Rev. 2011 Jun 15;(6):CD004718. doi: 10.1002/14651858.CD004718.pub3.

11. Preti A, Cella M. Randomized-controlled trials in people at ultra-high risk of psychosis: a review of treatment effectiveness. Schizophr Res. 2010 Oct;123(1):30-6. doi: 10.1016/j.schres.2010.07.026. Epub 2010 Aug 21.

12. Chatterjee S, Biondi-Zoccai G, Abbate A, et al.. Benefits of β blockers in patients with heart failure and reduced ejection fraction: network meta-analysis. BMJ. 2013 Jan 16;346:f55. doi:10.1136/bmj.f55.

13. Abdulla J, Køber L, Christensen E, Torp-Pedersen C. Effect of beta-blocker therapy on functional status in patients with heart failure--a meta-analysis. Eur J Heart Fail. 2006 Aug;8(5):522-31. Epub 2005 Dec 22.

14. Bouzamondo A, Hulot JS, Sanchez P, Lechat P. Beta-blocker benefit according to severity of heart failure. Eur J Heart Fail. 2003 Jun;5(3):281-9.

15. McAlister FA, Wiebe N, Ezekowitz JA, Leung AA, Armstrong PW. Meta-analysis: beta blocker dose, heart rate reduction, and death in patients with heart failure. Ann Intern Med. 2009 Jun 2;150(11):784-94.

16. Haase N, Perner A, Hennings LI, et al. Hydroxyethyl starch 130/0.38-0.45 versus crystalloid or albumin in patients with sepsis: systematic review with meta-analysis and trial sequential analysis. BMJ. 2013 Feb 15;346:f839. doi: 10.1136/bmj.f839.

17. Bunn F, Trivedi D. Colloid solutions for fluid resuscitation. Cochrane Database Syst Rev. 2012 Jul 11;7:CD001319. doi: 10.1002/14651858.CD001319.pub5.

18. Dart AB, Mutter TC, Ruth CA, Taback SP. Hydroxyethyl starch (HES) versus other fluid therapies: effects on kidney function. Cochrane Database Syst Rev. 2010 Jan 20;(1):CD007594. doi: 10.1002/14651858.CD007594.pub2.

19. Gattas DJ, Dan A, Myburgh J, Billot L, Lo S, Finfer S; CHEST Management Committee. Fluid resuscitation with 6% hydroxyethyl starch (130/0.4) in acutely ill patients: an updated systematic review and meta-analysis. Anesth Analg. 2012 Jan;114(1):159-69. doi: 10.1213/ANE.0b013e318236b4d6.

20. Groeneveld AB, Navickis RJ, Wilkes MM. Update on the comparative safety of colloids: a systematic review of clinical studies. Ann Surg. 2011 Mar;253(3):470-83. doi:10.1097/SLA.0b013e318202ff00.

21. Hartog CS, Kohl M, Reinhart K. A systematic review of third-generation hydroxyethyl starch (HES 130/0.4) in resuscitation: safety not adequately addressed. Anesth Analg. 2011 Mar;112(3):635-45. doi: 10.1213/ANE.0b013e31820ad607. Epub 2011 Feb 8.

22. Perel P, Roberts I. Colloids versus crystalloids for fluid resuscitation in critically ill patients. Cochrane Database Syst Rev. 2011 Mar 16;(3):CD000567. doi:10.1002/14651858.CD000567.pub4.

23. Bellemain-Appaix A, O'Connor SA, Silvain J, et al. ACTION Group. Association of clopidogrel pretreatment with mortality, cardiovascular events, and major bleeding among patients undergoing percutaneous coronary intervention: a systematic review and meta-analysis. JAMA. 2012 Dec 19;308(23):2507-16. doi: 10.1001/jama.2012.50788.

24. Lotrionte M, Biondi-Zoccai GG, Agostoni P, et al. I. Meta-analysis appraising high clopidogrel loading in patients undergoing percutaneous coronary intervention. Am J Cardiol. 2007 Oct 15;100(8):1199-206. Epub 2007 Jul 30.

25. Hao PP, Zhang MX, Li RJ, et al. Clopidogrel 150 vs. 75 mg day(-1) in patients undergoing percutaneous coronary intervention: a metaanalysis. J Thromb Haemost. 2011 Apr;9(4):627-37. doi: 10.1111/j.15387836.2011.04216.x.

26. Sabatine MS, Hamdalla HN, Mehta SR, et al. Efficacy and safety of clopidogrel pretreatment before percutaneous coronaryintervention with and without glycoprotein IIb/IIIa inhibitor use. Am Heart J. 2008 May;155(5):910-7. doi: 10.1016/j.ahj.2007.11.020. Epub 2008 Feb 21.

27. Siller-Matula JM, Huber K, Christ G, et al. Impact of clopidogrel loading dose on clinical outcome in patients undergoing percutaneous coronary intervention: a systematic review and meta-analysis. Heart. 2011 Jan;97(2):98-105. doi: 10.1136/hrt.2010.195438. Epub 2010 Aug 23.

28. Johnston BC, Ma SS, Goldenberg JZ, et al. Probiotics for the prevention of Clostridium difficile-associated diarrhea: a systematic review and meta-analysis. Ann Intern Med. 2012 Dec 18;157(12):878-88.

29. Avadhani A, Miley H. Probiotics for prevention of antibiotic-associated diarrhea and Clostridium difficile-associated disease in hospitalized adults--a meta-analysis. J Am Acad Nurse Pract. 2011 Jun;23(6):269-74. doi: 10.1111/j.1745-7599.2011.00617.x. Epub 2011 Apr 27.

30. D'Souza AL, Rajkumar C, Cooke J, Bulpitt CJ. Probiotics in prevention of antibiotic associated diarrhoea: meta-analysis. BMJ. 2002 Jun 8;324(7350):1361.

31. Hempel S, Newberry SJ, Maher AR, et al. Probiotics for the prevention and treatment of antibiotic-associated diarrhea: a systematic review and meta-analysis. JAMA. 2012 May 9;307(18):1959-69. doi: 10.1001/jama.2012.3507.

32. Johnston BC, Supina AL, Vohra S. Probiotics for pediatric antibiotic-associated diarrhea: a meta-analysis of randomized placebo-controlled trials. CMAJ. 2006 Aug 15;175(4):377-83.

33. Johnston BC, Goldenberg JZ, Vandvik PO, Sun X, Guyatt GH. Probiotics for the prevention of pediatric antibiotic-associated diarrhea. Cochrane Database Syst Rev. 2011 Nov 9;(11):CD004827. doi: 10.1002/14651858.CD004827.pub3.

34. McFarland LV. Meta-analysis of probiotics for the prevention of antibiotic associated diarrhea and the treatment of Clostridium difficile disease. Am J Gastroenterol. 2006 Apr;101(4):812-22.

35. Ritchie ML, Romanuk TN. A meta-analysis of probiotic efficacy for gastrointestinal diseases. PLoS One. 2012;7(4):e34938. doi: 10.1371/journal.pone.0034938. Epub 2012 Apr 18.

36. Segarra-Newnham M. Probiotics for Clostridium difficile-associated diarrhea: focus on Lactobacillus rhamnosus GG and Saccharomyces boulardii. Ann Pharmacother. 2007 Jul;41(7):1212-21. Epub 2007 Jun 26.

37. Szajewska H, Ruszczyński M, Radzikowski A. Probiotics in the prevention of antibiotic associated diarrhea in children: a meta-analysis of randomized controlled trials. J Pediatr. 2006 Sep;149(3):367-372.

38. Dendukuri N1, Costa V, McGregor M, Brophy JM. Probiotic therapy for the prevention and treatment of Clostridium difficile-associated diarrhea: a systematic review. CMAJ. 2005 Jul 19;173(2):167-70.

39. Pinto RZ, Maher CG, Ferreira ML, et al. Epidural corticosteroid injections in the management of sciatica: a systematic review and meta-analysis. Ann Intern Med. 2012 Dec 18;157(12):865-77.

40. Luijsterburg PA, Verhagen AP, Ostelo RW, van Os TA, Peul WC, Koes BW. Effectiveness of conservative treatments for the lumbosacral radicular syndrome: a systematic review. Eur Spine J. 2007 Jul;16(7):881-99. Epub 2007 Apr 6.

41. Quraishi NA. Transforaminal injection of corticosteroids for lumbar radiculopathy: systematic review and meta-analysis. Eur Spine J. 2012 Feb;21(2):214-9. doi:10.1007/s00586-011-2008-y. Epub 2011 Sep 4.

42. Huedo-Medina TB, Kirsch I, Middlemass J, Klonizakis M, Siriwardena AN. Effectiveness of non-benzodiazepine hypnotics in treatment of adult insomnia: meta-analysis of data submitted to the Food and Drug Administration. BMJ. 2012 Dec 17;345:e8343. doi:10.1136/bmj.e8343.

43. Buscemi N, Vandermeer B, Friesen C, et al. The efficacy and safety of drug treatments for chronic insomnia in adults: a meta-analysis of RCTs. J Gen Intern Med. 2007 Sep;22(9):1335-50. Epub 2007 Jul 10.

44. Dündar Y, Boland A, Strobl J, et al. Newer hypnotic drugs for the short-term management of insomnia: a systematic review and economic evaluation. Health Technol Assess. 2004 Jun;8(24):iii-x, 1-125.

45. Dündar Y, Dodd S, Strobl J, Boland A, Dickson R, Walley T. Comparative efficacy of newer hypnotic drugs for the short-term management of insomnia: a systematic review and meta-analysis. Hum Psychopharmacol. 2004 Jul;19(5):305-22.

46. Glass J, Lanctôt KL, Herrmann N, Sproule BA, Busto UE. Sedative hypnotics in older people with insomnia: meta-analysis of risks and benefits. BMJ. 2005 Nov 19;331(7526):1169. Epub 2005 Nov 11.

47. Fox BD, Kahn SR, Langleben D, Eisenberg MJ, Shimony A. Efficacy and safety of novel oral anticoagulants for treatment of acute venous thromboembolism: direct and adjusted indirect meta-analysis of randomised controlled trials. BMJ. 2012 Nov 13;345:e7498. doi: 10.1136/bmj.e7498.

48. Loke YK, Kwok CS. Dabigatran and rivaroxaban for prevention of venous hromboembolism--systematic review and adjusted indirect comparison. J Clin Pharm Ther. 2011 Feb;36(1):111-24. doi: 10.1111/j.1365-2710.2010.01162.x.

49. Low EV, Avery AJ, Gupta V, Schedlbauer A, Grocott MP. Identifying the lowest effective dose of acetazolamide for the prophylaxis of acute mountain sickness: systematic review and meta-analysis. BMJ. 2012 Oct 18;345:e6779. doi: 10.1136/bmj.e6779.

50. Seupaul RA, Welch JL, Malka ST, Emmett TW. Pharmacologic prophylaxis for acute mountain sickness: a systematic shortcut review. Ann Emerg Med. 2012 Apr;59(4):307-317.e1. doi: 10.1016/j.annemergmed.2011.10.015. Epub 2011 Dec 7.

51. Kayser B, Dumont L, Lysakowski C, Combescure C, Haller G, Tramèr MR. Reappraisal of acetazolamide for the prevention of acute mountain sickness: a systematic review and meta-analysis. High Alt Med Biol. 2012 Jun;13(2):82-92. doi: 10.1089/ham.2011.1084.

52. Zarychanski R, Abou-Setta AM, Turgeon AF, et al. Association of hydroxyethyl starch administration with mortality and acute kidney injury in critically ill patients requiring volume resuscitation: a systematic review and meta-analysis. JAMA. 2013 Feb 20;309(7):678-88. doi:10.1001/jama.2013.430.

53. Zarychanski R, Turgeon AF, Fergusson DA, et al. Renal outcomes and mortality following hydroxyethyl starch resuscitation of critically ill patients: systematic review and meta-analysis of randomized trials. Open Med. 2009;3(4):e196-209. Epub 2009 Oct 27.

54. Bischoff-Ferrari HA, Willett WC, Orav EJ, et al. A pooled analysis of vitamin D dose requirements for fracture prevention. N Engl J Med. 2012 Jul 5;367(1):40-9. doi: 10.1056/NEJMoa1109617.

55. Bergman GJ, Fan T, McFetridge JT, Sen SS. Efficacy of vitamin D3 supplementation in preventing fractures in elderly women: a meta-analysis. Curr Med Res Opin. 2010 May;26(5):1193-201. doi: 10.1185/03007991003659814.

56. Bischoff-Ferrari HA, Willett WC, Wong JB, et al. Prevention of nonvertebral fractures with oral vitamin D and dose dependency: a meta-analysis of randomized controlled trials. Arch Intern Med. 2009 Mar 23;169(6):551-61. doi: 10.1001/archinternmed.2008.600.

57. Izaks GJ. Fracture prevention with vitamin D supplementation: considering the inconsistent results. BMC Musculoskelet Disord. 2007 Mar 9;8:26.

58. Lai JK, Lucas RM, Clements MS, Roddam AW, Banks E. Hip fracture risk in relation to vitamin D supplementation and serum 25-hydroxyvitamin D levels: a systematic review and meta-analysis of randomised controlled trials and observational studies. BMC Public Health. 2010 Jun 11;10:331. doi: 10.1186/1471-2458-10-331.

59. Mosekilde L, Vestergaard P, Langdahl B. Fracture prevention in postmenopausal women. Clin Evid (Online). 2007 Sep 1;2007. pii: 1109.

60. Cranney A, Horsley T, O'Donnell S, et al. Effectiveness and safety of vitamin D in relation to bone health. Evid Rep Technol Assess (Full Rep). 2007 Aug;(158):1-235.

61. Boonen S, Lips P, Bouillon R, Bischoff-Ferrari HA, Vanderschueren D, Haentjens P. Need for additional calcium to reduce the risk of hip fracture with vitamin d supplementation: evidence from a comparative metaanalysis of randomized controlled trials. J Clin Endocrinol Metab. 2007 Apr;92(4):1415-23. Epub 2007 Jan 30.

62. Avenell A, Gillespie WJ, Gillespie LD, O'Connell D. Vitamin D and vitamin D analogues for preventing fractures associated with involutional and post-menopausal osteoporosis. Cochrane Database Syst Rev. 2009 Apr 15;(2):CD000227. doi:10.1002/14651858.CD000227.pub3.

63. Abrahamsen B, DIPART (Vitamin D Individual Patient Analysis of Randomized Trials) Group. Patient level pooled analysis of 68 500 patients from seven major vitamin D fracture trials in US and Europe. BMJ. 2010 Jan 12;340:b5463. doi:10.1136/bmj.b5463.

64. Haas DM, Caldwell DM, Kirkpatrick P, McIntosh JJ, Welton NJ. Tocolytic therapy for preterm delivery: systematic review and network meta-analysis. BMJ. 2012 Oct 9;345:e6226. doi: 10.1136/bmj.e6226.

65. Berkman ND, Thorp JM Jr, Lohr KN, et al. Tocolytic treatment for the management of preterm labor: a review of the evidence. Am J Obstet Gynecol. 2003 Jun;188(6):1648-59.

66. Coomarasamy A, Knox EM, Gee H, Khan KS. Oxytocin antagonists for tocolysis in preterm labour -- a systematic review. Med Sci Monit. 2002 Nov;8(11):RA268-73.

67. Haas DM, Imperiale TF, Kirkpatrick PR, Klein RW, Zollinger TW, Golichowski AM. Tocolytic therapy: a meta-analysis and decision analysis. Obstet Gynecol. 2009 Mar;113(3):585-94. doi: 10.1097/AOG.0b013e318199924a.

68. Li X, Zhang Y, Shi Z. Ritodrine in the treatment of preterm labour: a meta-analysis. Indian J Med Res. 2005 Feb;121(2):120-7.

69. Tan TC, Devendra K, Tan LK, Tan HK. Tocolytic treatment for the management of preterm labour: a systematic review. Singapore Med J. 2006 May;47(5):361-6.

70. Yaju Y, Nakayama T. Effectiveness and safety of ritodrine hydrochloride for the treatment of preterm labour: a systematic review. Pharmacoepidemiol Drug Saf. 2006 Nov;15(11):813-22.

71. King J, Flenady V, Cole S, Thornton S. Cyclo-oxygenase (COX) inhibitors for treating preterm labour. Cochrane Database Syst Rev. 2005 Apr 18;(2):CD001992.

72. Anotayanonth S, Subhedar NV, Garner P, Neilson JP, Harigopal S. Betamimetics for inhibiting preterm labour. Cochrane Database Syst Rev. 2004 Oct 18;(4):CD004352.

73. Crowther CA, Hiller JE, Doyle LW. Magnesium sulphate for preventing preterm birth in threatened preterm labour. Cochrane Database Syst Rev. 2006;(4):CD001060.

74. King JF, Flenady VJ, Papatsonis DN, Dekker GA, Carbonne B. Calcium channel blockers for inhibiting preterm labour. Cochrane Database Syst Rev. 2003;(1):CD002255.

75. Papatsonis D, Flenady V, Cole S, Liley H. Oxytocin receptor antagonists for inhibiting preterm labour. Cochrane Database Syst Rev. 2005 Jul 20;(3):CD004452.

76. MacArthur GJ, Minozzi S, Martin N, et al. Opiate substitution treatment and HIV transmission in people who inject drugs: systematic review and meta-analysis. BMJ. 2012 Oct 3;345:e5945. doi:10.1136/bmj.e5945.

77. Hedrich D, Alves P, Farrell M, Stöver H, Møller L, Mayet S. The effectiveness of opioid maintenance treatment in prison settings: a systematic review. Addiction. 2012 Mar;107(3):501-17. doi: 10.1111/j.1360-0443.2011.03676.x.

78. Gowing L, Farrell MF, Bornemann R, Sullivan LE, Ali R. Oral substitution treatment of injecting opioid users for prevention of HIV infection. Cochrane Database Syst Rev. 2011 Aug 10;(8):CD004145. doi: 10.1002/14651858.CD004145.pub4.

79. Larney S. Does opioid substitution treatment in prisons reduce injecting-related HIV risk behaviours? A systematic review. Addiction. 2010 Feb;105(2):216-23. doi:10.1111/j.1360-0443.2009.02826.x.

80. Hartling L, Abou-Setta AM, Dursun S, Mousavi SS, Pasichnyk D, Newton AS. Antipsychotics in adults with schizophrenia: comparative effectiveness of first generation versus second-generation medications: a systematic review and metaanalysis. Ann Intern Med. 2012 Oct 2;157(7):498-511.

81. Bhattacharjee J, El-Sayeh HG. Aripiprazole versus typical antipsychotic drugs for schizophrenia. Cochrane Database Syst Rev. 2008 Jul 16;(3):CD006617. doi:10.1002/14651858.CD006617.pub3.

82. Crossley NA, Constante M, McGuire P, Power P. Efficacy of atypical v. typical antipsychotics in the treatment of early psychosis: meta-analysis. Br J Psychiatry. 2010 Jun;196(6):434-9. doi: 10.1192/bjp.bp.109.066217.

83. Davis JM, Chen N, Glick ID. A meta-analysis of the efficacy of second-generation antipsychotics. Arch Gen Psychiatry. 2003 Jun;60(6):553-64.

84. Essali A, Al-Haj Haasan N, Li C, Rathbone J. Clozapine versus typical neurolepticmedication for schizophrenia. Cochrane Database Syst Rev. 2009 Jan 21;(1):CD000059. doi: 10.1002/14651858.CD000059.pub2.

85. Klemp M, Tvete IF, Skomedal T, Gaasemyr J, Natvig B, Aursnes I. A review and Bayesian meta-analysis of clinical efficacy and adverse effects of 4 atypical neuroleptic drugs compared with haloperidol and placebo. J Clin Psychopharmacol. 2011 Dec;31(6):698-704. doi: 10.1097/JCP.0b013e31823657d9.

86. Leucht S, Barnes TR, Kissling W, Engel RR, Correll C, Kane JM. Relapse prevention in schizophrenia with new-generation antipsychotics: a systematic review and exploratory meta-analysis of randomized, controlled trials. Am J Psychiatry. 2003 Jul;160(7):1209-22.

87. Leucht S, Corves C, Arbter D, Engel RR, Li C, Davis JM. Second-generation versus first generation antipsychotic drugs for schizophrenia: a meta-analysis. Lancet. 2009 Jan 3;373(9657):31-41. doi: 10.1016/S0140-6736(08)61764-X. Epub 2008 Dec 6.

88. Smith M, Hopkins D, Peveler RC, Holt RI, Woodward M, Ismail K. First- v. second generation antipsychotics and risk for diabetes in schizophrenia: systematic review and meta-analysis. Br J Psychiatry. 2008 Jun;192(6):406-11. doi: 10.1192/bjp.bp.107.037184.

89. Hutton B, Joseph L, Fergusson D, Mazer CD, Shapiro S, Tinmouth A. Risks of harms using antifibrinolytics in cardiac surgery: systematic review and network meta-analysis of randomised and observational studies. BMJ. 2012 Sep 11;345:e5798. doi:10.1136/bmj.e5798.

90. McIlroy DR, Myles PS, Phillips LE, Smith JA. Antifibrinolytics in cardiac surgical patients receiving aspirin: a systematic review and meta-analysis. Br J Anaesth. 2009 Feb;102(2):168-78. doi: 10.1093/bja/aen377.

91. Ngaage DL, Bland JM. Lessons from aprotinin: is the routine use and inconsistent dosing of tranexamic acid prudent? Meta-analysis of randomised and large matched observational studies. Eur J Cardiothorac Surg. 2010 Jun;37(6):1375-83. doi:10.1016/j.ejcts.2009.11.055. Epub 2010 Feb 1..

92. Umscheid CA, Kohl BA, Williams K. Antifibrinolytic use in adult cardiac surgery. Curr Opin Hematol. 2007 Sep;14(5):455-67..

93. Brown JR, Birkmeyer NJ, O'Connor GT. Meta-analysis comparing the effectiveness and adverse outcomes of antifibrinolytic agents in cardiac surgery. Circulation. 2007 Jun 5;115(22):2801-13. Epub 2007 May 28.

94. Lopez-Olivo MA, Tayar JH, Martinez-Lopez JA, et al. Risk of malignancies in patients with rheumatoid arthritis treated with biologic therapy: a meta-analysis. JAMA. 2012 Sep 5;308(9):898-908. doi: 10.1001/2012.jama.10857.

95. Bongartz T, Sutton AJ, Sweeting MJ, Buchan I, Matteson EL, Montori V. Anti-TNF antibody therapy in rheumatoid arthritis and the risk of serious infections and malignancies: systematic review and meta-analysis of rare harmful effects in randomized controlled trials. JAMA. 2006 May 17;295(19):2275-85.

96. Askling J, Fahrbach K, Nordstrom B, Ross S, Schmid CH, Symmons D. Cancer risk with tumor necrosis factor alpha (TNF) inhibitors: meta-analysis of randomized controlled trials of adalimumab, etanercept, and infliximab using patient level data. Pharmacoepidemiol Drug Saf. 2011 Feb;20(2):119-30. doi: 10.1002/pds.2046. Epub 2010 Dec 7.

97. Bongartz T, Warren FC, Mines D, Matteson EL, Abrams KR, Sutton AJ. Etanercept therapy in rheumatoid arthritis and the risk of malignancies: a systematic review and individual patient data meta-analysis of randomised controlled trials. Ann Rheum Dis. 2009 Jul;68(7):1177-83. doi: 10.1136/ard.2008.094904. Epub 2008 Nov 19.

98. Salliot C, van der Heijde D. Long-term safety of methotrexate monotherapy in patients with rheumatoid arthritis: a systematic literature research. Ann Rheum Dis. 2009Jul;68(7):1100-4. doi: 10.1136/ard.2008.093690. Epub 2008 Dec 5.

99. Thompson AE, Rieder SW, Pope JE. Tumor necrosis factor therapy and the risk of serious infection and malignancy in patients with early rheumatoid arthritis: a meta-analysis of randomized controlled trials. Arthritis Rheum. 2011 Jun;63(6):1479-85. doi:10.1002/art.30310.

100. Kaiser R. Incidence of lymphoma in patients with rheumatoid arthritis: a systematic review of the literature. Clin Lymphoma Myeloma. 2008 Apr;8(2):87-93. doi:10.3816/CLM.2008.n.009.

101. LE Blay P, Mouterde G, Barnetche T, Morel J, Combe B. Short-term risk of total malignancy and nonmelanoma skin cancers with certolizumab and golimumab in

patients with rheumatoid arthritis: metaanalysis of randomized controlled trials. J Rheumatol. 2012 Apr;39(4):712-5. doi: 10.3899/jrheum.110982. Epub 2012 Mar 1.

102. Leombruno JP, Einarson TR, Keystone EC. The safety of anti-tumour necrosis factor treatments in rheumatoid arthritis: meta and exposure-adjusted pooled analyses of serious adverse events. Ann Rheum Dis. 2009 Jul;68(7):1136-45. doi:10.1136/ard.2008.091025. Epub 2008 Aug 27.

103. Nannini C, Cantini F, Niccoli L, et al. Single-center series and systematic review of randomized controlled trials of malignancies in patients with rheumatoid arthritis, psoriatic arthritis, and ankylosing spondylitis receiving anti-tumor necrosis factor alpha therapy: is there a need for more comprehensive screening procedures? Arthritis Rheum. 2009 Jun 15;61(6):801-12. doi: 10.1002/art.24506.

104. Wiens A, Correr CJ, Venson R, Grochocki MC, Otuki MF, Pontarolo R. A meta-analysis of the efficacy and safety of using infliximab for the treatment of rheumatoid arthritis. Clin Rheumatol. 2009a Dec;28(12):1365-73. doi: 10.1007/s10067-009-1233-9. Epub 2009 Sep 22.

105. Wiens A, Correr CJ, Pontarolo R, Venson R, Quinalha JV, Otuki MF. A systematic review and meta-analysis of the efficacy and safety of etanercept for treating rheumatoid arthritis. Scand J Immunol. 2009b Oct;70(4):337-44. doi: 10.1111/j.1365 3083.2009.02296.x.

106. Wiens A, Correr CJ, Venson R, Otuki MF, Pontarolo R. A systematic review and meta-analysis of the efficacy and safety of adalimumab for treating rheumatoid arthritis. Rheumatol Int. 2010a Jun;30(8):1063-70. doi: 10.1007/s00296-009-1111-4. Epub 2009 Aug 26.

107. Wiens A, Venson R, Correr CJ, Otuki MF, Pontarolo R. Meta-analysis of the efficacy and safety of adalimumab, etanercept, and infliximab for the treatment of rheumatoid arthritis. Pharmacotherapy. 2010b Apr;30(4):339-53. doi: 10.1592/phco.30.4.339.

108. Wong AK, Kerkoutian S, Said J, Rashidi H, Pullarkat ST. Risk of lymphoma in patients receiving antitumor necrosis factor therapy: a meta-analysis of published randomized controlled studies. Clin Rheumatol. 2012 Apr;31(4):631-6. doi: 10.1007/s10067-011 1895-y. Epub 2011 Nov 18.

109. Rahimi K, Bhala N, Kamphuisen P, et al. Effect of statins on venous thromboembolic events: a metaanalysis of published and unpublished evidence from randomised controlled trials. PLoS Med. 2012;9(9):e1001310. doi:10.1371/journal.pmed.1001310. Epub 2012 Sep 18

110. Agarwal V, Phung OJ, Tongbram V, Bhardwaj A, Coleman CI. Statin use and the prevention of venous thromboembolism: a meta-analysis. Int J Clin Pract. 2010 Sep;64(10):1375 83. doi: 10.1111/j.1742-1241.2010.02439.x.

111. Pai M, Evans NS, Shah SJ, Green D, Cook D, Crowther MA. Statins in the prevention of venous thromboembolism: a meta-analysis of observational studies. Thromb Res. 2011 Nov;128(5):422-30. doi: 10.1016/j.thromres.2011.05.012.

112. Squizzato A, Galli M, Romualdi E, et al. Statins, fibrates, and venous thromboembolism: a meta-analysis. Eur Heart J. 2010 May;31(10):1248-56. doi: 10.1093/eurheartj/ehp556. Epub 2009 Dec 22.

113. Plante J, Turgeon AF, Zarychanski R, et al. Effect of systemic steroids on post-tonsillectomy bleeding and reinterventions: systematic review and meta-analysis of randomised controlled trials. BMJ. 2012 Aug 28;345:e5389. doi: 10.1136/bmj.e5389.

114. Geva A, Brigger MT. Dexamethasone and tonsillectomy bleeding: a meta-analysis. Otolaryngol Head Neck Surg. 2011 Jun;144(6):838-43. doi: 10.1177/0194599811399538. Epub 2011 Mar 31.

115. Diakos EA, Gallos ID, El-Shunnar S, Clarke M, Kazi R, Mehanna H. Dexamethasone reduces pain, vomiting and overall complications following tonsillectomy in adults: a systematic review and meta-analysis of randomised controlled trials. Clin Otolaryngol. 2011 Dec;36(6):531-42. doi: 10.1111/j.1749-4486.2011.02373.x.

116. Preiss D, Tikkanen MJ, Welsh P, et al. Lipid-modifying therapies and risk of pancreatitis: a meta-analysis. JAMA. 2012 Aug 22;308(8):804-11. doi:10.1001/jama.2012.8439.

117. Singh S, Loke YK. Statins and pancreatitis: a systematic review of observational studies and spontaneous case reports. Drug Saf. 2006;29(12):1123-32.

118. Palmer SC, Craig JC, Navaneethan SD, Tonelli M, Pellegrini F, Strippoli GF. Benefits and harms of statin therapy for persons with chronic kidney disease: a systematic review and meta-analysis. Ann Intern Med. 2012 Aug 21;157(4):263-75. doi: 10.7326/0003 4819-157-4-201208210-00007.

119. Navaneethan SD, Nigwekar SU, Perkovic V, Johnson DW, Craig JC, Strippoli GF. HMG CoA reductase inhibitors (statins) for dialysis patients. Cochrane Database Syst Rev. 2009a Jul 8;(3):CD004289. doi: 10.1002/14651858.CD004289.pub4.

120. Navaneethan SD, Perkovic V, Johnson DW, Nigwekar SU, Craig JC, Strippoli GF. HMG CoA reductase inhibitors (statins) for kidney transplant recipients. Cochrane Database Syst Rev. 2009b Apr 15;(2):CD005019. doi: 10.1002/14651858.CD005019.pub3.

121. Navaneethan SD, Pansini F, Perkovic V, et al. HMG CoA reductase inhibitors (statins) for people with chronic kidney disease not requiring dialysis. Cochrane Database Syst Rev. 2009 Apr 15;(2):CD007784. doi: 10.1002/14651858.CD007784.

122. Strippoli GF, Navaneethan SD, Johnson DW, et al. Effects of statins in patients with chronic kidney disease: meta-analysis and meta regression of randomised controlled trials. BMJ. 2008 Mar 22;336(7645):645-51. doi:10.1136/bmj.39472.580984.AE. Epub 2008 Feb 25

123. Upadhyay A, Earley A, Lamont JL, Haynes S, Wanner C, Balk EM. Lipid-lowering therapy in persons with chronic kidney disease: a systematic review and meta-analysis. Ann Intern Med. 2012 Aug 21;157(4):251-62. doi: 10.7326/0003-4819-157-4-201208210 00005.

124. Mihaylova B, Emberson J, Blackwell L, et al., Cholesterol Treatment Trialists' (CTT) Collaborators. The effects of lowering LDL cholesterol with statin therapy in people at low risk of vascular disease: meta-analysis of individual data from 27 randomised trials. Lancet. 2012 Aug 11;380(9841):581-90. doi: 10.1016/S0140-6736(12)60367-5. Epub 2012 May 17.

125. CTT (Cholesterol Treatment Trialists' Collaboration), Baigent C, Blackwell L, Emberson J, et al. Efficacy and safety of more intensive lowering of LDL cholesterol: a meta-analysis of data from 170,000 participants in 26 randomised trials. Lancet. 2010 Nov 13;376(9753):1670-81. doi: 10.1016/S0140-6736(10)61350-5. Epub 2010 Nov 8.

126. Taylor F, Ward K, Moore TH, et al. Statins for the primary prevention of cardiovascular disease. Cochrane Database Syst Rev. 2011 Jan 19;(1):CD004816. doi: 10.1002/14651858.CD004816.pub4.

127. Bangalore S, Kumar S, Fusaro M, et al. Outcomes with various drug eluting or bare metal stents in patients with diabetes mellitus: mixed treatment comparison analysis of 22,844 patient years of follow-up from randomised trials. BMJ. 2012 Aug 10;345:e5170. doi:10.1136/bmj.e5170.

128. Boyden TF, Nallamothu BK, Moscucci M, et al. Meta-analysis of randomized trials of drug-eluting stents versus bare metal stents in patients with diabetes mellitus. Am J Cardiol. 2007 May 15;99(10):1399-402. Epub 2007 Apr 4.

129. Kirtane AJ, Ellis SG, Dawkins KD, et al. Paclitaxel-eluting coronary stents in patients with diabetes mellitus: pooled analysis from 5 randomized trials. J Am Coll Cardiol. 2008 Feb 19;51(7):708-15. doi: 10.1016/j.jacc.2007.10.035.

130. Mahmud E, Bromberg-Marin G, Palakodeti V, Ang L, Creanga D, Demaria AN. Clinical efficacy of drug-eluting stents in diabetic patients: a meta-analysis. J Am Coll Cardiol. 2008 Jun 24;51(25):2385-95. doi: 10.1016/j.jacc.2008.03.028.

131. Patti G, Nusca A, Di Sciascio G. Meta-analysis comparison (nine trials) of outcomes with drug-eluting stents versus bare metal stents in patients with diabetes mellitus. Am J Cardiol. 2008 Nov 15;102(10):1328-34. doi: 10.1016/j.amjcard.2008.07.012. Epub 2008 Sep 6.

132. Stettler C, Allemann S, Wandel S, et al. Drug eluting and bare metal stents in people with and without diabetes: collaborative network meta-analysis. BMJ. 2008 Aug 29;337:a1331. doi: 10.1136/bmj.a1331.

133. de Waha A, Dibra A, Kufner S, et al. Long-term outcome after sirolimus-eluting stents versus bare metal stents in patients with diabetes mellitus: a patient-level meta-analysis of randomized trials. Clin Res Cardiol. 2011 Jul;100(7):561-70. doi: 10.1007/s00392-010-0278-8. Epub 2011 Jan 8

134. Zhang F, Dong L, Ge J. Meta-analysis of five randomized clinical trials comparing sirolimus versus paclitaxel-eluting stents in patients with diabetes mellitus. Am J Cardiol. 2010 Jan 1;105(1):64-8. doi: 10.1016/j.amjcard.2009.08.652. Epub 2009 Nov 14.

135. Scheen AJ, Warzée F, Legrand VM. Drug-eluting stents: meta-analysis in diabetic patients. Eur Heart J. 2004 Dec;25(23):2167-8; author reply 2168-9.

136. Mantha S, Karp R, Raghavan V, Terrin N, Bauer KA, Zwicker JI. Assessing the risk of venous thromboembolic events in women taking progestin-only contraception: a meta-analysis. BMJ. 2012 Aug 7;345:e4944. doi: 10.1136/bmj.e4944.

137. Bergendal A, Odlind V, Persson I, Kieler H. Limited knowledge on progestogen-only contraception and risk of venous thromboembolism. Acta Obstet Gynecol Scand. 2009;88(3):261-6. doi: 10.1080/00016340902730375.

138. Rutjes AW, Jüni P, da Costa BR, Trelle S, Nüesch E, Reichenbach S. Viscosupplementation for osteoarthritis of the knee: a systematic review and meta-analysis. Ann Intern Med. 2012 Aug 7;157(3):180-91. doi: 10.7326/0003-4819-157-3-201208070-00473.

139. Aggarwal A, Sempowski IP. Hyaluronic acid injections for knee osteoarthritis. Systematic review of the literature. Can Fam Physician. 2004 Feb;50:249-56.

140. Conrozier T, Vignon E. Is there evidence to support the inclusion of viscosupplementation in the treatment paradigm for patients with hip osteoarthritis? Clin Exp Rheumatol. 2005 Sep-Oct;23(5):711-6.

141. Divine JG, Zazulak BT, Hewett TE. Viscosupplementation for knee osteoarthritis: a systematic review. Clin Orthop Relat Res. 2007 Feb;455:113-22.

142. Lo GH, LaValley M, McAlindon T, Felson DT. Intra-articular hyaluronic acid in treatment of knee osteoarthritis: a meta-analysis. JAMA. 2003 Dec 17;290(23):3115-21.

143. Arrich J, Piribauer F, Mad P, Schmid D, Klaushofer K, Müllner M. Intra-articular hyaluronic acid for the treatment of osteoarthritis of the knee: systematic review and meta-analysis. CMAJ. 2005 Apr 12;172(8):1039-43.

144. Bannuru RR, Natov NS, Dasi UR, Schmid CH, McAlindon TE. Therapeutic trajectory following intra-articular hyaluronic acid injection in knee osteoarthritis—meta analysis. Osteoarthritis Cartilage. 2011 Jun;19(6):611-9. doi:10.1016/j.joca.2010.09.014. Epub 2011 Apr 9.

145. Bellamy N, Campbell J, Robinson V, Gee T, Bourne R, Wells G. Viscosupplementation for the treatment of osteoarthritis of the knee. Cochrane Database Syst Rev. 2006 Apr 19;(2):CD005321.

146. Medina JM, Thomas A, Denegar CR. Knee osteoarthritis: should your patient opt for hyaluronic acid injection? J Fam Pract. 2006 Aug;55(8):669-75.

147. Modawal A, Ferrer M, Choi HK, Castle JA. Hyaluronic acid injections relieve knee pain. J Fam Pract. 2005 Sep;54(9):758-67.

148. Wang CT, Lin J, Chang CJ, Lin YT, Hou SM. Therapeutic effects of hyaluronic acid on osteoarthritis of the knee. A meta-analysis of randomized controlled trials. J Bone Joint Surg Am. 2004 Mar;86-A(3):538-45.

149. Ahuja SD, Ashkin D, Avendano M, et al. Collaborative Group for Meta-Analysis of Individual Patient Data in MDR-TB. Multidrug resistant pulmonary tuberculosis treatment regimens and patient outcomes: an individual patient data meta-analysis of 9,153 patients. PLoS Med. 2012;9(8):e1001300. Epub 2012 Aug 28.

150. Johnston JC, Shahidi NC, Sadatsafavi M, Fitzgerald JM. Treatment outcomes of multidrug resistant tuberculosis: a systematic review and meta-analysis. PLoS One. 2009 Sep 9;4(9):e6914. doi: 10.1371/journal.pone.0006914.

151. Orenstein EW, Basu S, Shah NS, Andrews JR, Friedland GH, Moll AP, Gandhi NR, Galvani AP. Treatment outcomes among patients with multidrug-resistant tuberculosis: systematic review and meta-analysis. Lancet Infect Dis. 2009 Mar;9(3):153-61. doi:10.1016/S1473-3099(09)70041-6.

152. Lv J, Neal B, Ehteshami P, Ninomiya T, et al. Effects of intensive blood pressure lowering on cardiovascular and renal outcomes: a systematic review and meta-analysis. PLoS Med. 2012;9(8):e1001293. doi: 10.1371/journal.pmed.1001293. Epub 2012 Aug 21.

153. Reboldi G, Gentile G, Angeli F, Ambrosio G, Mancia G, Verdecchia P. Effects of intensive blood pressure reduction on myocardial infarction and stroke in diabetes: a meta-analysis in 73,913 patients. J Hypertens. 2011 Jul;29(7):1253-69. doi:10.1097/HJH.0b013e3283469976.

154. Arguedas JA, Perez MI, Wright JM. Treatment blood pressure targets for hypertension. Cochrane Database Syst Rev. 2009 Jul 8;(3):CD004349. doi:10.1002/14651858.CD004349.pub2.

155. Karagiannis T, Paschos P, Paletas K, Matthews DR, Tsapas A. Dipeptidyl peptidase-4 inhibitors for treatment of type 2 diabetes mellitus in the clinical setting: systematic review and meta-analysis. BMJ. 2012 Mar 12;344:e1369. doi: 10.1136/bmj.e1369.

156. Esposito K, Cozzolino D, Bellastella et al. Dipeptidyl peptidase-4 inhibitors and HbA1c target of <7% in type 2 diabetes: meta-analysis of randomized controlled trials. Diabetes Obes Metab. 2011 Jul;13(7):594 603. doi: 10.1111/j.1463-1326.2011.01380.x.

157. Monami M, Iacomelli I, Marchionni N, Mannucci E. Dipeptydil peptidase-4 inhibitors in type 2 diabetes: a meta-analysis of randomized clinical trials. Nutr Metab Cardiovasc Dis. 2010 May;20(4):224-35. doi: 10.1016/j.numecd.2009.03.015. Epub 2009 Jun 9.

158. Richter B, Bandeira-Echtler E, Bergerhoff K, Lerch CL. Dipeptidyl peptidase-4 (DPP-4) inhibitors for type 2 diabetes mellitus. Cochrane Database Syst Rev. 2008 Apr 16;(2):CD006739. doi: 10.1002/14651858.CD006739.pub2.

159. Richter B, Bandeira-Echtler E, Bergerhoff K, Lerch C. Emerging role of dipeptidyl peptidase-4 inhibitors in the management of type 2 diabetes. Vasc Health Risk Manag. 2008;4(4):753-68.

160. Wani JH, John-Kalarickal J, Fonseca VA. Dipeptidyl peptidase-4 as a new target of action for type 2 diabetes mellitus: a systematic review. Cardiol Clin. 2008 Nov;26(4):639-48. doi: 10.1016/j.ccl.2008.06.008.

161. Caldeira D, Alarcão J, Vaz-Carneiro A, Costa J. Risk of pneumonia associated with use of angiotensin converting enzyme inhibitors and angiotensin receptor blockers: systematic review and meta-analysis. BMJ. 2012 Jul 11;345:e4260. doi:10.1136/bmj.e4260.

162. Rafailidis PI, Matthaiou DK, Varbobitis I, Falagas ME. Use of ACE inhibitors and risk of community-acquired pneumonia: a review. Eur J Clin Pharmacol. 2008 Jun;64(6):565 73. doi: 10.1007/s00228-008-0467-9.

163. Siempos II, Vardakas KZ, Kopterides P, Falagas ME. Adjunctive therapies for community acquired pneumonia: a systematic review. J Antimicrob Chemother. 2008 Oct;62(4):661-8. doi: 10.1093/jac/dkn283. Epub 2008 Jul 18.

164. El Solh AA, Saliba R. Pharmacologic prevention of aspiration pneumonia: a systematic review. Am J Geriatr Pharmacother. 2007 Dec;5(4):352-62. doi:10.1016/j.amjopharm.2007.12.005.

165. Suthar AB, Lawn SD, del Amo J, et al. Antiretroviral therapy for prevention of tuberculosis in adults with HIV: a systematic review and meta-analysis. PLoS Med. 2012;9(7):e1001270. doi: 10.1371/journal.pmed.1001270. Epub 2012 Jul 24.

166. Lawn SD, Wood R, De Cock KM, Kranzer K, Lewis JJ, Churchyard GJ. Antiretrovirals and isoniazid preventive therapy in the prevention of HIV-associated tuberculosis in settings with limited health-care resources. Lancet Infect Dis. 2010 Jul;10(7):489-98. doi: 10.1016/S1473-3099(10)70078-5.

167. Wardlaw JM, Murray V, Berge E, et al. Recombinant tissue plasminogen activator for acute ischaemic stroke: an updated systematic review and meta-analysis. Lancet. 2012 Jun 23;379(9834):2364-72. doi:10.1016/S0140-6736(12)60738-7. Epub 2012 May 23.

168. Bhatnagar P, Sinha D, Parker RA, Guyler P, O'Brien A. Intravenous thrombolysis in acute ischaemic stroke: a systematic review and meta-analysis to aid decision making in patients over 80 years of age. J Neurol Neurosurg Psychiatry. 2011 Jul;82(7):712-7. doi: 10.1136/jnnp.2010.223149. Epub 2011 Feb 3.

169. Wardlaw JM, Sandercock PA, Berge E. Thrombolytic therapy with recombinant tissue plasminogen activator for acute ischemic stroke: where do we go from here? A cumulative meta-analysis. Stroke. 2003 Jun;34(6):1437-42. Epub 2003 May 1.

170. Wardlaw JM, Murray V, Berge E, Del Zoppo GJ. Thrombolysis for acute ischaemic stroke. Cochrane Database Syst Rev. 2009 Oct 7;(4):CD000213. doi:10.1002/14651858.CD000213.pub2.

171. Gómez-Outes A, Terleira-Fernández AI, Suárez-Gea ML, Vargas-Castrillón E. Dabigatran, rivaroxaban, or apixaban versus enoxaparin for thromboprophylaxis after total hip or knee replacement: systematic review, meta-analysis, and indirect treatment comparisons. BMJ. 2012 Jun 14;344:e3675. doi: 10.1136/bmj.e3675.

172. Cao YB, Zhang JD, Shen H, Jiang YY. Rivaroxaban versus enoxaparin for thromboprophylaxis after total hip or knee arthroplasty: a meta-analysis of randomized controlled trials. Eur J Clin Pharmacol. 2010 Nov;66(11):1099-108. doi:10.1007/s00228-010-0889-z. Epub 2010 Sep 2.

173. Huang J, Cao Y, Liao C, Wu L, Gao F. Apixaban versus enoxaparin in patients with total knee arthroplasty. A meta-analysis of randomised trials. Thromb Haemost. 2011 Feb;105(2):245-53. doi: 10.1160/TH10-08-0552. Epub 2010 Oct 12.

174. Turun S, Banghua L, Yuan Y, Zhenhui L, Ying N, Jin C. A systematic review of rivaroxaban versus enoxaparin in the prevention of venous thromboembolism after hip or knee replacement. Thromb Res. 2011 Jun;127(6):525-34. doi:10.1016/j.thromres.2011.01.016. Epub 2011 Mar 12.

175. Maratea D, Fadda V, Trippoli S, Messori A. Prevention of venous thromboembolism after major orthopedic surgery: indirect comparison of three new oral anticoagulants. J Thromb Haemost. 2011 Sep;9(9):1868-70. doi: 10.1111/j.1538-7836.2011.04421.x.

176. Trkulja V, Kolundzic R. Rivaroxaban vs dabigatran for thromboprophylaxis after joint replacement surgery: exploratory indirect comparison based on meta-analysis of pivotal clinical trials. Croat Med J. 2010 Apr;51(2):113-23.

177. Jardine MJ, Kang A, Zoungas S, et al. The effect of folic acid based homocysteine lowering on cardiovascular events in people with kidney disease: systematic review and meta-analysis. BMJ. 2012 Jun 13;344:e3533. doi: 10.1136/bmj.e3533.

178. Bazzano LA, Reynolds K, Holder KN, He J. Effect of folic acid supplementation on risk of cardiovascular diseases: a meta-analysis of randomized controlled trials. JAMA. 2006 Dec 13;296(22):2720-6.

179. Clarke R, Halsey J, Lewington S, et al., B-Vitamin Treatment Trialists' Collaboration. Effects of lowering homocysteine levels with B vitamins on cardiovascular disease, cancer, and cause-specific mortality: Meta-analysis of 8 randomized trials involving 37 485 individuals. Arch Intern Med. 2010 Oct 11;170(18):1622-31. doi: 10.1001/archinternmed.2010.348.

180. Qin X, Huo Y, Langman CB, et al. Folic acid therapy and cardiovascular disease in ESRD or advanced chronic kidney disease: a meta-analysis. Clin J Am Soc Nephrol. 2011 Mar;6(3):482-8. doi:10.2215/CJN.05310610. Epub 2010 Nov 18.

181. Wang X, Qin X, Demirtas H, et al. Efficacy of folic acid supplementation in stroke prevention: a meta-analysis. Lancet. 2007 Jun 2;369(9576):1876-82.

182. Leucht S, Tardy M, Komossa K, et al. Antipsychotic drugs versus placebo for relapse prevention in schizophrenia: a systematic review and meta-analysis. Lancet. 2012 Jun 2;379(9831):2063-71. doi: 10.1016/S0140 6736(12)60239-6. Epub 2012 May 3.

183. Alvarez-Jiménez M1, Parker AG, Hetrick SE, McGorry PD, Gleeson JF. Preventing the second episode: a systematic review and meta-analysis of psychosocial and pharmacological trials in first-episode psychosis. Schizophr Bull. 2011 May;37(3):619-30. doi: 10.1093/schbul/sbp129. Epub 2009 Nov 9.

184. Ker K, Edwards P, Perel P, Shakur H, Roberts I. Effect of tranexamic acid on surgical bleeding: systematic review and cumulative meta-analysis. BMJ. 2012 May 17;344:e3054. doi: 10.1136/bmj.e3054.

185. Adler Ma SC, Brindle W, Burton G, et al. Tranexamic acid is associated with less blood transfusion in off-pump coronary artery bypass graft surgery: a systematic review and meta-analysis. J Cardiothorac Vasc Anesth. 2011 Feb;25(1):26-35. doi:10.1053/j.jvca.2010.08.012. Epub 2010 Nov 5.

186. Alshryda S, Sarda P, Sukeik M, Nargol A, Blenkinsopp J, Mason JM. Tranexamic acid in total knee replacement: a systematic review and meta-analysis. J

Bone Joint Surg Br. 2011 Dec;93(12):1577-85. doi: 10.1302/0301-620X.93B12.26989.

187. Cid J, Lozano M. Tranexamic acid reduces allogeneic red cell transfusions in patients undergoing total knee arthroplasty: results of a meta-analysis of randomized controlled trials. Transfusion. 2005 Aug;45(8):1302-7.

188. Elgafy H, Bransford RJ, McGuire RA, Dettori JR, Fischer D. Blood loss in major spine surgery: are there effective measures to decrease massive hemorrhage in major spine fusion surgery? Spine (Phila Pa 1976). 2010 Apr 20;35(9 Suppl):S47-56. doi:10.1097/BRS.0b013e3181d833f6.

189. Gill JB, Rosenstein A. The use of antifibrinolytic agents in total hip arthroplasty: a meta-analysis. J Arthroplasty. 2006 Sep;21(6):869-73.

190. Gill JB, Chin Y, Levin A, Feng D. The use of antifibrinolytic agents in spine surgery. A meta-analysis. J Bone Joint Surg Am. 2008 Nov;90(11):2399-407. doi:10.2106/JBJS.G.01179.

191. Guay J, de Moerloose P, Lasne D. Minimizing perioperative blood loss and transfusions in children. Can J Anaesth. 2006 Jun;53(6 Suppl):S59-67.

192. Gurusamy KS, Li J, Sharma D, Davidson BR. Pharmacological interventions to decrease blood loss and blood transfusion requirements for liver resection. Cochrane Database Syst Rev. 2009 Oct 7;(4):CD008085. doi: 10.1002/14651858.CD008085.

193. Ho KM, Ismail H. Use of intravenous tranexamic acid to reduce allogeneic blood transfusion in total hip and knee arthroplasty: a meta-analysis. Anaesth Intensive Care. 2003 Oct;31(5):529-37.

194. Kagoma YK, Crowther MA, Douketis J, Bhandari M, Eikelboom J, Lim W. Use of antifibrinolytic therapy to reduce transfusion in patients undergoing orthopedic surgery: a systematic review of randomized trials. Thromb Res. 2009 Mar;123(5):687 96. doi: 10.1016/j.thromres.2008.09.015. Epub 2008 Nov 12.

195. Kongnyuy EJ, Wiysonge CS. Interventions to reduce haemorrhage during myomectomy for fibroids. Cochrane Database Syst Rev. 2009 Jul 8;(3):CD005355. doi:10.1002/14651858.CD005355.pub3.

196. Liu JM, Peng HM, Shen JX, Qiu GX. A meta-analysis of the effectiveness and safety of using tranexamic acid in spine surgery. Zhonghua Wai Ke Za Zhi. 2010 Jun 15;48(12):937 42.

197. Makwana J, Paranjape S, Goswami J. Antifibrinolytics in liver surgery. Indian J Anaesth. 2010 Nov;54(6):489-95. doi: 10.4103/0019-5049.72636.

198. Martin-Hirsch PP, Keep SL, Bryant A. Interventions for preventing blood loss during the treatment of cervical intraepithelial neoplasia. Cochrane Database Syst Rev. 2010 Jun 16;(6):CD001421. doi: 10.1002/14651858.CD001421.pub2.

199. Molenaar IQ, Warnaar N, Groen H, Tenvergert EM, Slooff MJ, Porte RJ. Efficacy and safety of antifibrinolytic drugs in liver transplantation: a systematic review and meta-analysis. Am J Transplant. 2007 Jan;7(1):185-94.

200. Novikova N, Hofmeyr GJ. Tranexamic acid for preventing postpartum haemorrhage. Cochrane Database Syst Rev. 2010 Jul 7;(7):CD007872. doi:10.1002/14651858.CD007872.pub2.

201. Schouten ES, van de Pol AC, Schouten AN, Turner NM, Jansen NJ, Bollen CW. The effect of aprotinin, tranexamic acid, and aminocaproic acid on blood loss and use of blood products in major pediatric surgery: a meta-analysis. Pediatr Crit Care Med. 2009 Mar;10(2):182-90. doi: 10.1097/PCC.0b013e3181956d61.

202. Sukeik M, Alshryda S, Haddad FS, Mason JM. Systematic review and meta-analysis of the use of tranexamic acid in total hip replacement. J Bone Joint Surg Br. 2011 Jan;93(1):39-46. doi: 10.1302/0301-620X.93B1.24984.

203. Tzortzopoulou A, Cepeda MS, Schumann R, Carr DB. Antifibrinolytic agents for reducing blood loss in scoliosis surgery in children. Cochrane Database Syst Rev. 2008 Jul 16;(3):CD006883. doi: 10.1002/14651858.CD006883.pub2.

204. Zhang H, Chen J, Chen F, Que W. The effect of tranexamic acid on blood loss and use of blood products in total knee arthroplasty: a meta-analysis. Knee Surg Sports Traumatol Arthrosc. 2012 Sep;20(9):1742-52. doi: 10.1007/s00167-011-1754-z. Epub 2011 Nov 8.

205. Zufferey P, Merquiol F, Laporte S, et al. Do antifibrinolytics reduce allogeneic blood transfusion in orthopedic surgery? Anesthesiology. 2006 Nov;105(5):1034-46.

206. Abrishami A, Chung F, Wong J.Topical application of antifibrinolytic drugs for on-pump cardiac surgery: a systematic review and meta-analysis. Can J Anaesth. 2009 Mar;56(3):202-12. doi: 10.1007/s12630-008-9038-x. Epub 2009 Feb 12.

207. Carless PA, Moxey AJ, Stokes BJ, Henry DA. Are antifibrinolytic drugs equivalent in reducing blood loss and transfusion in cardiac surgery? A meta-analysis of randomized head-to-head trials. BMC Cardiovasc Disord. 2005 Jul 4;5:19.

208. Takagi H, Manabe H, Kawai N, Goto SN, Umemoto T. Aprotinin increases mortality as compared with tranexamic acid in cardiac surgery: a meta-analysis of randomized head-to-head trials. Interact Cardiovasc Thorac Surg. 2009 Jul;9(1):98-101. doi:10.1510/icvts.2008.198325. Epub 2009 Apr 20.

209. Neumann I, Rada G, Claro JC, et al. Oral direct Factor Xa inhibitors versus low-molecular-weight heparin to prevent venous thromboembolism in patients undergoing total hip or knee replacement: a systematic review and meta-analysis. Ann Intern Med. 2012 May 15;156(10):710-9. doi: 10.7326/0003-4819-156-10-201205150-00421. Epub 2012 Mar 12.

210. Boekholdt SM, Arsenault BJ, Mora S, et al. Association of LDL cholesterol, non-HDL cholesterol, and apolipoprotein B levels with risk of cardiovascular events among patients treated with statins: a meta-analysis. JAMA. 2012 Mar 28;307(12):1302-9. doi: 10.1001/jama.2012.366.

211. Sazawal S, Hiremath G, Dhingra U, Malik P, Deb S, Black RE. Efficacy of probiotics in prevention of acute diarrhoea: a meta-analysis of masked, randomised, placebo controlled trials. Lancet Infect Dis. 2006 Jun;6(6):374-82.

212. Jackson JL, Kuriyama A, Hayashino Y. Botulinum toxin A for prophylactic treatment of migraine and tension headaches in adults: a meta-analysis. JAMA. 2012 Apr 25;307(16):1736-45. doi: 10.1001/jama.2012.505.

213. Gupta VK. Botulinum toxin--a treatment for migraine? A systematic review. Pain Med. 2006 Sep-Oct;7(5):386-94.

214. Shuhendler AJ, Lee S, Siu M, et al. Efficacy of botulinum toxin type A for the prophylaxis of episodic migraine headaches: a meta-analysis of randomized, double-blind, placebo-controlled trials. Pharmacotherapy. 2009 Jul;29(7):784-91. doi: 10.1592/phco.29.7.784.

215. Hemmingsen B, Christensen LL, Wetterslev J, et al. Comparison of metformin and insulin versus insulin alone for type 2 diabetes: systematic review of randomised clinical trials with meta-analyses and trial sequential analyses. BMJ. 2012 Apr 19;344:e1771. doi: 10.1136/bmj.e1771.

216. Palmerini T, Biondi-Zoccai G, Della Riva D, et al. Stent thrombosis with drug-eluting and bare-metal stents: evidence from a comprehensive network meta-analysis. Lancet. 2012 Apr 14;379(9824):1393-402. doi: 10.1016/S0140-6736(12)60324-9. Epub 2012 Mar 23.

217. Milić NM, Parapid BJ, Ostojić MC, Nedeljković MA, Marinković JM. Meta-analysis of stent thrombosis after drug-eluting stent implantation: 4-year follow-up. Chin Med J (Engl). 2010 Dec;123(24):3689-96.

218. Baber U, Mehran R, Sharma SK, et al. Impact of the everolimus-eluting stent on stent thrombosis: a meta-analysis of 13 randomized trials. J Am Coll Cardiol. 2011 Oct 4;58(15):1569-77. doi: 10.1016/j.jacc.2011.06.049. Epub 2011 Sep 14.

219. Roukoz H, Bavry AA, Sarkees ML, Mood GR, Kumbhani DJ, Rabbat MG, Bhatt DL. Comprehensive meta-analysis on drug-eluting stents versus bare-metal stents during extended follow-up. Am J Med. 2009 Jun;122(6):581.e1-10. doi:10.1016/j.amjmed.2008.12.019.

220. Mauri L, Hsieh WH, Massaro JM, Ho KK, D'Agostino R, Cutlip DE. Stent thrombosis in randomized clinical trials of drug-eluting stents. N Engl J Med. 2007 Mar 8;356(10):1020-9. Epub 2007 Feb 12.

221. Varadhan KK, Neal KR, Lobo DN. Safety and efficacy of antibiotics compared with appendicectomy for treatment of uncomplicated acute appendicitis: meta-analysis of randomised controlled trials. BMJ. 2012 Apr 5;344:e2156. doi: 10.1136/bmj.e2156.

222. Ansaloni L, Catena F, Coccolini F, et al. Surgery versus conservative antibiotic treatment in acute appendicitis: a systematic review and meta-analysis of randomized controlled trials. Dig Surg. 2011;28(3):210-21. doi:10.1159/000324595. Epub 2011 May 3.

223. Fitzmaurice GJ, McWilliams B, Hurreiz H, Epanomeritakis E. Antibiotics versus appendectomy in the management of acute appendicitis: a review of the current evidence. Can J Surg. 2011 Oct;54(5):307-14. doi: 10.1503/cjs.006610.

224. Liu K, Fogg L. Use of antibiotics alone for treatment of uncomplicated acute appendicitis: a systematic review and meta-analysis. Surgery. 2011 Oct;150(4):673-83. doi:10.1016/j.surg.2011.08.018.

225. Varadhan KK, Humes DJ, Neal KR, Lobo DN. Antibiotic therapy versus appendectomy for acute appendicitis: a meta-analysis. World J Surg. 2010 Feb;34(2):199-209. doi:10.1007/s00268-009-0343-5.

226. Wilms IM, de Hoog DE, de Visser DC, Janzing HM. Appendectomy versus antibiotic treatment for acute appendicitis. Cochrane Database Syst Rev. 2011 Nov 9;(11):CD008359. doi: 10.1002/14651858.CD008359.pub2.

227. Hsu J, Santesso N, Mustafa R, et al. Antivirals for treatment of influenza: a systematic review and meta-analysis of observational studies. Ann Intern Med. 2012 Apr 3;156(7):512-24. doi: 10.7326/0003-4819-156-7-201204030-00411. Epub 2012 Feb 27.

228. Burch J, Paulden M, Conti S, et al. Antiviral drugs for the treatment of influenza: a systematic review and economic evaluation. Health Technol Assess. 2009 Nov;13(58):1-265, iii-iv. doi:10.3310/hta13580.

229. Cooper NJ, Sutton AJ, Abrams KR, Wailoo A, Turner D, Nicholson KG. Effectiveness of neuraminidase inhibitors in treatment and prevention of influenza A and B: systematic review and meta-analyses of randomised controlled trials. BMJ. 2003 Jun 7;326(7401):1235.

230. Falagas ME, Koletsi PK, Vouloumanou EK, Rafailidis PI, Kapaskelis AM, Rello J. Effectiveness and safety of neuraminidase inhibitors in reducing influenza complications: a meta-analysis of randomized controlled trials. J Antimicrob Chemother. 2010 Jul;65(7):1330-46. doi: 10.1093/jac/dkq158. Epub 2010 May 20.

231. Jackson RJ, Cooper KL, Tappenden P, Rees A, Simpson EL, Read RC, Nicholson KG. Oseltamivir, zanamivir and amantadine in the prevention of influenza:

a systematic review. J Infect. 2011 Jan;62(1):14-25. doi: 10.1016/j.jinf.2010.10.003. Epub 2010 Oct 13.

232. Jefferson T, Demicheli V, Rivetti D, Jones M, Di Pietrantonj C, Rivetti A. Antivirals for influenza in healthy adults: systematic review. Lancet. 2006 Jan 28;367(9507):303-13.

233. Jefferson T, Jones M, Doshi P, Del Mar C. Neuraminidase inhibitors for preventing and treating influenza in healthy adults: systematic review and meta-analysis. BMJ. 2009 Dec 8;339:b5106. doi: 10.1136/bmj.b5106.

234. Khazeni N, Bravata DM, Holty JE, Uyeki TM, Stave CD, Gould MK. Systematic review: safety and efficacy of extended-duration antiviral chemoprophylaxis against pandemic and seasonal influenza. Ann Intern Med. 2009 Oct 6;151(7):464-73. Epub 2009 Aug 3.

235. Shun-Shin M, Thompson M, Heneghan C, Perera R, Harnden A, Mant D. Neuraminidase inhibitors for treatment and prophylaxis of influenza in children: systematic review and meta-analysis of randomised controlled trials. BMJ. 2009 Aug 10;339:b3172. doi: 10.1136/bmj.b3172.

236. Tappenden P, Jackson R, Cooper K, et al. Amantadine, oseltamivir and zanamivir for the prophylaxis of influenza (including a review of existing guidance no. 67): a systematic review and economic evaluation. Health Technol Assess. 2009 Feb;13(11):iii, ix-xii, 1-246. doi: 10.3310/hta13110.

237. Alves Galvão MG1, Rocha Crispino Santos MA, Alves da Cunha AJ. Amantadine and rimantadine for influenza A in children and the elderly. Cochrane Database Syst Rev. 2012 Jan 18;1:CD002745. doi: 10.1002/14651858.CD002745.pub3.

238. Jefferson T, Jones M, Doshi P, Del Mar C, Dooley L, Foxlee R. Neuraminidase inhibitors for preventing and treating influenza in healthy adults. Cochrane Database Syst Rev. 2010 Feb 17;(2):CD001265. doi: 10.1002/14651858.CD001265.pub3.

239. Jefferson T, Jones MA, Doshi P, et al. Neuraminidase inhibitors for preventing and treating influenza in healthy adults and children. Cochrane Database Syst Rev. 2012 Jan 18;1:CD008965. doi: 10.1002/14651858.CD008965.pub3.

240. Wang K, Shun-Shin M, Gill P, Perera R, Harnden A. Neuraminidase inhibitors for preventing and treating influenza in children (published trials only). Cochrane Database Syst Rev. 2012 Apr 18;4:CD002744. doi: 10.1002/14651858.CD002744.pub4.

241. Boussageon R, Supper I, Bejan-Angoulvant T, et al. Reappraisal of metformin efficacy in the treatment of type 2 diabetes: a meta-analysis of randomised controlled trials. PLoS Med. 2012;9(4):e1001204. doi: 10.1371/journal.pmed.1001204. Epub 2012 Apr 10.

242. Eurich DT, McAlister FA, Blackburn DF, et al. Benefits and harms of antidiabetic agents in patients with diabetes and heart failure: systematic review. BMJ. 2007 Sep 8;335(7618):497. Epub 2007 Aug 30.

243. Lamanna C, Monami M, Marchionni N, Mannucci E. Effect of metformin on cardiovascular events and mortality: a meta-analysis of randomized clinical trials. Diabetes Obes Metab. 2011 Mar;13(3):221-8. doi: 10.1111/j.1463-1326.2010.01349.x.

244. Sáenz Calvo A, Fernández Esteban I, Mataix Sanjuán A, et al. [Metformin for type-2 diabetes mellitus. Systematic review and meta-analysis]. Aten Primaria. 2005 Sep 15;36(4):183-91.

245. Saenz A, Fernandez-Esteban I, Mataix A, Ausejo M, Roque M, Moher D. Metformin monotherapy for type 2 diabetes mellitus. Cochrane Database Syst Rev. 2005 Jul 20;(3):CD002966.

246. Palmer SC, Di Micco L, Razavian M, et al. Effects of antiplatelet therapy on mortality and cardiovascular and bleeding outcomes in persons with chronic kidney disease: a systematic review and meta-analysis. Ann Intern Med. 2012 Mar 20;156(6):445-59. doi: 10.7326/0003-4819-156-6-201203200000007.

247. McKnight RF, Adida M, Budge K, Stockton S, Goodwin GM, Geddes JR. Lithium toxicity profile: a systematic review and meta-analysis. Lancet. 2012 Feb 25;379(9817):721-8. doi: 10.1016/S0140-6736(11)61516-X. Epub 2012 Jan 20.

248. Ceron-Litvoc D, Soares BG, Geddes J, Litvoc J, de Lima MS. Comparison of carbamazepine and lithium in treatment of bipolar disorder: a systematic review of randomized controlled trials. Hum Psychopharmacol. 2009 Jan;24(1):19-28. doi: 10.1002/hup.990.

249. Paul R, Minay J, Cardwell C, Fogarty D, Kelly C. Meta-analysis of the effects of lithium usage on serum creatinine levels. J Psychopharmacol. 2010 Oct;24(10):1425-31. doi:10.1177/0269881109104930. Epub 2009 Apr 24.

250. Pinto RZ, Maher CG, Ferreira ML, et al. Drugs for relief of pain in patients with sciatica: systematic review and meta-analysis. BMJ. 2012 Feb 13;344:e497. doi: 10.1136/bmj.e497

251. Deshpande A, Furlan A, Mailis-Gagnon A, Atlas S, Turk D. Opioids for chronic low-back pain. Cochrane Database Syst Rev. 2007 Jul 18;(3):CD004959.

252. Chou R, Huffman LH; American Pain Society; American College of Physicians. Medications for acute and chronic low back pain: a review of the evidence for an American Pain Society/American College of Physicians clinical practice guideline. Ann Intern Med. 2007 Oct 2;147(7):505-14.

253. Roelofs PD, Deyo RA, Koes BW, Scholten RJ, van Tulder MW. Non-steroidal antiinflammatory drugs for low back pain. Cochrane Database Syst Rev. 2008 Jan 23;(1):CD000396. doi: 10.1002/14651858.CD000396.pub3.

254. EBCTCG (Early Breast Cancer Trialists' Collaborative Group), Peto R, Davies C, Godwin J, et al. Comparisons between different polychemotherapy regimens for early breast cancer: meta-analyses of long term outcome among 100,000 women in 123 randomised trials. Lancet. 2012 Feb 4;379(9814):432-44. doi: 10.1016/S0140-6736(11)61625-5. Epub 2011 Dec 5

255. Dowsett M, Cuzick J, Ingle J, et al. Meta-analysis of breast cancer outcomes in adjuvant trials of aromatase inhibitors versus tamoxifen. J Clin Oncol. 2010 Jan 20;28(3):509 18. doi: 10.1200/JCO.2009.23.1274. Epub 2009 Nov 30.

256. EBCTCG (Early Breast Cancer Trialists' Collaborative Group). Effects of chemotherapy and hormonal therapy for early breast cancer on recurrence and 15-year survival: an overview of the randomised trials. Lancet. 2005 May 14-20;365(9472):1687-717.

257. EBCTCG (Early Breast Cancer Trialists' Collaborative Group), Davies C, Godwin J, Gray R, et al. Relevance of breast cancer hormone receptors and other factors to the efficacy of adjuvant tamoxifen: patient-level meta-analysis of randomised trials. Lancet. 2011 Aug 27;378(9793):771-84. doi: 10.1016/S0140-6736(11)60993-8. Epub 2011 Jul 28.

258. Silvain J, Beygui F, Barthélémy O, et al. Efficacy and safety of enoxaparin versus unfractionated heparin during percutaneous coronary intervention: systematic review and meta-analysis. BMJ. 2012 Feb 3;344:e553. doi: 10.1136/bmj.e553.

259. Borentain M, Montalescot G, Bouzamondo A, Choussat R, Hulot JS, Lechat P. Low molecular-weight heparin vs. unfractionated heparin in percutaneous coronary intervention: a combined analysis. Catheter Cardiovasc Interv. 2005 Jun;65(2):212-21.

260. Dumaine R, Borentain M, Bertel O, et al. Intravenous low-molecular-weight heparins compared with unfractionated heparin in percutaneous coronary intervention: quantitative review of randomized trials. Arch Intern Med. 2007 Dec 10;167(22):2423-30.

261. Navarese EP, De Luca G, Castriota F, et al. Low-molecular weight heparins vs. unfractionated heparin in the setting of percutaneous coronary intervention for ST-elevation myocardial infarction: a meta-analysis. J Thromb Haemost. 2011 Oct;9(10):1902-15. doi: 10.1111/j.1538-7836.2011.04445.x.

262. Vilsbøll T, Christensen M, Junker AE, Knop FK, Gluud LL. Effects of glucagon-like peptide 1 receptor agonists on weight loss: systematic review and meta-analyses of randomised controlled trials. BMJ. 2012 Jan 10;344:d7771. doi: 10.1136/bmj.d7771.

263. Amori RE, Lau J, Pittas AG. Efficacy and safety of incretin therapy in type 2 diabetes: systematic review and meta-analysis. JAMA. 2007 Jul 11;298(2):194-206.

264. Monami M, Marchionni N, Mannucci E. Glucagon-like peptide-1 receptor agonists in type 2 diabetes: a meta-analysis of randomized clinical trials. Eur J Endocrinol. 2009 Jun;160(6):909-17. doi: 10.1530/EJE-09-0101. Epub 2009 Mar 24

265. Norris SL, Lee N, Thakurta S, Chan BK. Exenatide efficacy and safety: a systematic review. Diabet Med. 2009 Sep;26(9):837-46. doi: 10.1111/j.1464-5491.2009.02790.x.

266. Shyangdan DS, Royle PL, Clar C, Sharma P, Waugh NR. Glucagon-like peptide analogues for type 2 diabetes mellitus: systematic review and meta-analysis. BMC Endocr Disord. 2010 Dec 9;10:20. doi: 10.1186/1472-6823-10-20.

267. Shyangdan DS, Royle P, Clar C, Sharma P, Waugh N, Snaith A. Glucagon-like peptide analogues for type 2 diabetes mellitus. Cochrane Database Syst Rev. 2011 Oct 5;(10):CD006423. doi: 10.1002/14651858.CD006423.pub2.
